# Multivalent ion binding site identification with structure-based deep learning

**DOI:** 10.1038/s42003-026-10659-1

**Published:** 2026-07-27

**Authors:** Igor Kozlovskii, Petr Popov

**Affiliations:** 1 Constructor University Bremen gGmbH, Bremen, Germany; 2 Constructor Labs, Bremen, Germany

**Keywords:** Machine learning, Molecular biology

## Abstract

Protein-ion interactions are essential for many cellular processes, including enzymatic catalysis, signaling, and allosteric regulation. However, mapping ion-binding sites experimentally remains labor-intensive and expensive. Here, we present BiteNet_I_, a structure-based deep learning model that uses 3D convolutional neural networks to simultaneously localize ion-binding centers and predict binding residues for 14 biologically relevant ions. Trained on a carefully curated dataset of over 10,000 high-resolution protein–ion complexes, in which near-identical binding sites are consistently annotated by transferring ions between homologous structures, BiteNet_I_ shows strong generalization ability across diverse ions within a unified multitask architecture. On two different test benchmarks, BiteNet_I_ achieves state-of-the-art performance compared to existing ion-binding predictors as well as to a more general method, AlphaFold3, when used to predict the entire structure of protein bound to ions. Finally, for physiologically relevant ions such as Ca^2+^, Na^+^ and K^+^, BiteNet_I_ achieves two- to three-fold improvement in accuracy.

## Abstract (web summary)

Structure-based deep learning enables accurate prediction of protein-ion binding sites across 14 biologically relevant ions, supporting comprehensive and large-scale annotation of protein-ion interactions.

## Introduction

Ions play fundamental roles in protein function, mediating enzymatic catalysis, signal transduction, structural stabilization, and allosteric regulation^1^. Ions can bind to protein active sites^2^, stabilize or induce conformational changes in protein structure^3,4^, modulate the activity of DNA/RNA polymerases^5^, or influence the rate of concentration-dependent protein aggregation^6^. Furthermore, ions could play a role as allosteric modulators, for example, the sodium ion in G protein-coupled receptors^7^; in the calcium-sensing receptor (CaSR) Ca^2+^ and Mg^2+^ act as activators, Cl^−^ as a positive allosteric modulator, and ${{{{\rm{SO}}}}}_{4}^{2-}$/${{{{\rm{PO}}}}}_{4}^{3-}$ as negative allosteric modulators^8^; Cl^−^ as allosteric modulator in metabotropic glutamate receptors (mGluRs)^9^; or Ca^2+^ in nicotinic acetylcholine receptors (nAChRs)^10^. Therefore, protein-ion interactions, and particularly protein-ion binding sites, are one of the key elements in understanding protein functioning. Importantly, ion binding sites differ from traditional small-molecule or peptide binding pockets in their size, geometry, and coordination chemistry. Moreover, ions are typically less selective compared to ligands, and can bind to different binding sites in terms of amino acid composition and geometry^11^. On the other hand, there are well-known ion binding sites with a very specific coordination pattern: for instance, Zn^2+^ typically coordinates with Cys, His, Asp, or Glu residues by 4 or 5 atoms with a distorted tetrahedral or trigonal bipyramidal geometry^12^. Despite their significance, ion-binding sites remain challenging to characterize experimentally, requiring high-resolution crystallography or spectroscopy. Computational methods offer a scalable alternative, yet current predictors are often limited to single ion types, rely on residue-level features, or fail to generalize across structurally diverse proteins^13^. Recently, Cheng et al.^14^ introduced a co-evolution-based machine-learning framework (MetalNet) that predicts metal-binding residue networks directly from multiple sequence alignments at proteome scale, further illustrating the potential of data-driven approaches for large-scale metalloproteome annotation. It is important to note that predicting ion-binding sites requires atomic-level precision. For example, the sodium binding site in G protein-coupled receptors is conformation-specific; it can be observed in the inactive-like conformation but collapsed in the active-like conformation^7^. Therefore, a robust prediction of protein-ion binding sites must take into account the atomic-level information of protein structures. Moreover, most existing tools either train separate models per ion type or lack the scalability for multi-ion prediction^13^. To address these challenges, we developed BiteNet_I_, a multitask deep learning framework based on 3D convolutional neural networks (3D-CNNs) that jointly predicts binding centers and residue-level scores for 14 biologically relevant ion types. Unlike existing approaches, BiteNet_I_ processes full atomic representations of protein structures and learns shared features across ion classes, enhancing generalization and robustness. BiteNet_I_ also benefits from a manually curated dataset of ~10,000 protein-ion complex structures and a carefully designed train-validation-test split that accounts for sequence-, structure-, and binding-site-based similarities. Extensive benchmarking demonstrates that BiteNet_I_ outperforms existing state-of-the-art methods for most ion types. In particular, it achieves up to threefold improvements in the Matthews correlation coefficient (MCC) over traditional models for Ca^2+^, Na^+^ and K^+^ prediction. Notably, BiteNet_I_ also surpasses the recently released AlphaFold3 on these benchmarks, while offering much faster inference times. This highlights its practical value for large-scale annotation of ion-binding sites in both native and predicted protein structures. Finally, we addressed the importance of structural resolution and of water molecules coordinating ion binding for protein-ion binding site prediction.

## Results and discussion

### Model architecture and performance

**Fig. 1:**
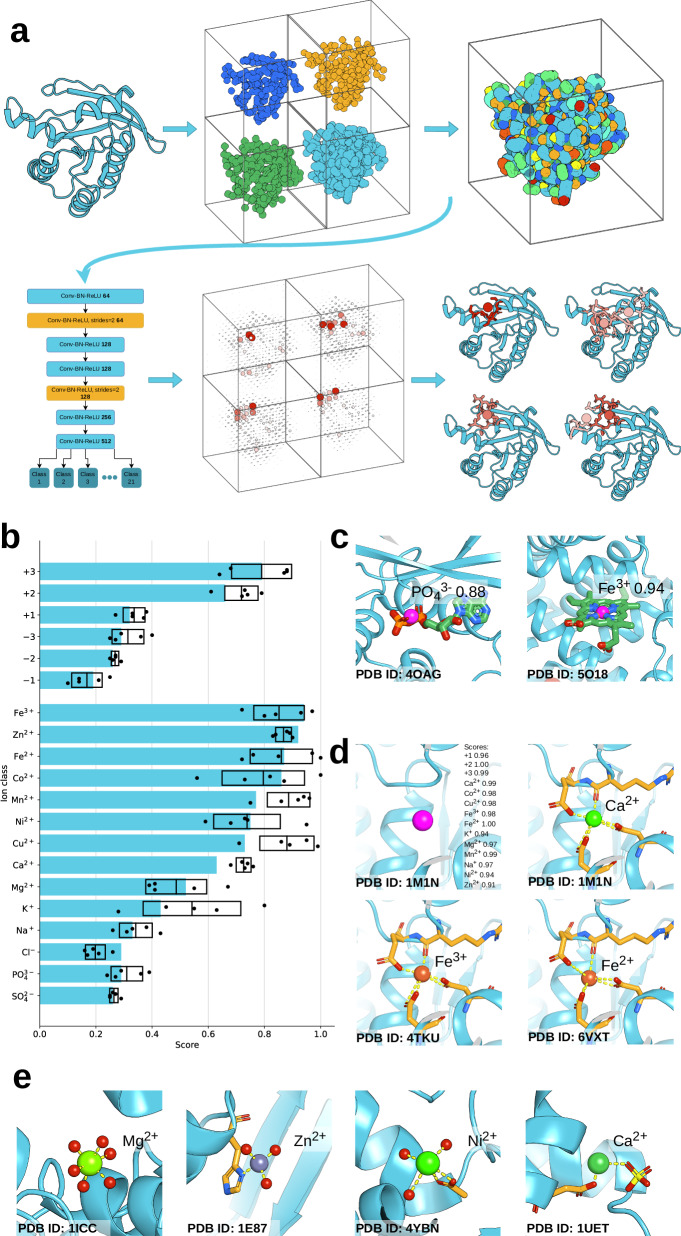
BiteNet_I_ architecture, model performance and representative prediction examples. **a** Overview of the BiteNet_I_ pipeline. Input structure is voxelized into a 64 × 64 × 64 grids with 11 channels. Then a 3D-CNN processes the grid into a 16 × 16 × 16 feature map; each cell predicts binding probability and center coordinates for 20 ion classes that are mapped into the input structure to obtain the final predictions. Binding site center and residues predictions are represented with spheres and sticks, respectively. Spheres and residues are colored with red according to predicted scores. **b** Weighted average precision (wAP) scores for each ion class. Black points show scores from individual cross-validation folds; boxplots summarize the distribution across *n* = 5 folds. Cyan bars show single aggregate scores on the independent test set. **c** Examples of “false” positive predictions, that correspond to non-ion molecules in the structures. The ${{{{\rm{PO}}}}}_{4}^{3-}$ prediction corresponds to the phosphate part of ADP (PDB ID: 4OAG). The Fe^3+^ prediction corresponds to the HEM center containing Fe^3+^ ion (PDB ID: 5O18). Ligand molecules are shown with sticks, predictions are represented with magenta spheres, and labels show the prediction scores. **d** Examples of different structures of the Nitrogenase MoFe protein with various ions in the same binding site. Expectedly, BiteNet_I_ predicts high scores for different ions in such binding sites. Binding amino acid residues are represented with yellow sticks. **e** Examples of false-negative (zero-recall) cases of BiteNet_I_ on the test set. Ions are shown as spheres; nearby coordinating atoms within 3.0 Å are shown as sticks/spheres; dashed lines indicate coordination distances. Mg^2+^ (PDB ID: 1ICC; chain A, MG 88) is coordinated exclusively by six water molecules (no protein atoms within 3.0 Å involved in coordination). Zn^2+^ (PDB ID: 1E87; chain A, ZN 1001) is coordinated by His116 (ND1) and three water molecules. Ni^2+^ (PDB ID: 4YBN; chain A, NI 301) was refined with partial occupancy (0.20) and coordinated by Asp125, a sulfate oxygen, and a water molecule. Ca^2+^ (PDB ID: 1UET; chain A, CA 501) is primarily coordinated by acetate (ACT 601) and water molecules (no protein atoms within 3.0 Å involved in coordination).

Inspired by the spatiotemporal approach for identification of small molecule and peptide binding sites^15,16^, in this work, we have developed a multi-task deep learning approach, dubbed BiteNet_I_, for protein-ion binding site detection of various ion types. BiteNet_I_ encodes each protein structure as a voxelized 3D grid (64 × 64 × 64 voxels at 1 Å resolution) with 11 atom-type channels, where voxel intensities reflect summed Gaussian densities of nearby atoms of the corresponding type (Eq. (1)). The backbone is a 3D convolutional neural network (3D-CNN) composed of repeated Conv–BN–ReLU blocks, which downsample the input grid to a 16 × 16 × 16 feature map. For every cell in this map, the network jointly predicts a binding-site probability and the center coordinates for each of the 20 ion classes. Figure 1a demonstrates a schematic overview of the BiteNet_I_ model pipeline. To enhance robustness, we used class- and ion-cluster-stratified train-validation splits along with the cluster-balanced sampling during training (see Section Training). BiteNet_I_ achieved a mean weighted average precision (mwAP) of 0.54 ± 0.01 (mean ± s.d. over *n* = 5 cross-validation folds), with consistent performance across the splits, indicating no apparent overfitting with respect to the training data. Figure 1b and Supplementary Tables 1 and 2 demonstrate the performances for each ion class on each set in more detail. Last but not least, computational speed is another important characteristic of the model. We observed that running a single multi-task model is  ~10 times faster compared to running several single task models (see Supplementary Fig. 1), while performance is almost the same (0.545 ± 0.016 against 0.541 ± 0.025; mean ± s.d. over *n* = 5 cross-validation folds). On average, it takes just 16.0 ± 0.5s to run a multi-task model for a single structure.

#### Error analysis

Consistent with chemical intuition, we observed that ion types with well-defined, strongly chelated coordination geometries, such as Zn^2+^, Fe^3+^/Fe^2+^, Mn^2+^, Co^2+^, and Cu^2+^ achieve the highest average precision (AP) and Matthews correlation coefficient (MCC) scores, whereas monovalent cations and anions with more diffuse and context-dependent coordination patterns (Na^+^, K^+^, Cl^−^, ${{{{\rm{SO}}}}}_{4}^{2-}$, ${{{{\rm{PO}}}}}_{4}^{3-}$) remain more challenging (Supplementary Table 2). Interestingly, when analyzing false-positive predictions, we observed that many of them correspond to the sites bound to charged small molecules, such as phosphate or heme cofactors. In the test set, approximately  ~ 7% of all predictions were located within 1.0 Å distance from a co-crystallized small molecule, compared to  ~ 20% for ions (see Supplementary Table 3 for more details). Figure 1c demonstrates two examples of such cases, including the prediction of ${{{{\rm{PO}}}}}_{4}^{3-}$ corresponding to the polar phosphate group in the ATP binding site and the prediction of Fe^3+^ corresponding to HEM. These findings suggest that BiteNet_I_ may recognize atomic coordination patterns common to both ions and polar functional groups in small molecules. On the other hand, we observed structurally conserved regions that bind different ions in different Protein Data Bank (PDB) structures: Figure 1d illustrates cases when similar binding sites hold different ions (also see Supplementary Fig. 2 for a case, when ions are present in one structure, but absent in the other). These observations suggest that ion-based profiling of protein structures may be useful for pre-screening or prioritizing regions of interest for small molecules bearing charged chemical groups. As for the false negative examples, we inspected test-set structures for which BiteNet_I_ shows zero recall for a given ion class, i.e., no predicted sites of that class fall within the distance threshold (4 Å) of any annotated ion. Across the inspected cases, the missed ions frequently exhibit weak or ambiguous coordination patterns: many are predominantly solvent-coordinated (e.g., hexahydrated Mg2+) or stabilized by crystallization additives, such as acetate or sulfate, rather than by protein side chains. We also observed ions refined with partial occupancies (e.g., Ni^2+^ in PDB ID: 4YBN with occupancy 0.20), indicating weak support for the ion assignment and/or low site population. Figure 1e shows representative examples of such atomic environments. Together with the resolution-dependent analysis in Section Impact of structure resolution, these observations indicate that false predictions are enriched in structurally ambiguous regions (low-resolution or partially resolved coordination shells) and in sites that are chemically compatible with ion binding but are occupied by other charged cofactors in a given structure.

#### Ablation study

Note that we more than doubled the dataset by adding superimposed ions to highly similar binding sites (see Section Dataset construction) and used cluster-balanced sampling during training. To quantify the effect of this ion-transfer augmentation and the sampling strategy on the models’ performance, we conducted an ablation study (see Supplementary Table 4 for detailed results). We observed that training without transferred ions and with cluster-based sampling yields the highest mwAP on the original, non-augmented structures for the multi-task model (0.576 ± 0.023; mean ± s.d. over *n* = 5 cross-validation folds). Adding transferred ions and training with the same sampling scheme leads to a slightly lower mwAP on the original structures (0.545 ± 0.016; mean  ± s.d. over *n* = 5 cross-validation folds), and across all tested configurations (multi-task vs. single-task heads, cluster-based vs. random sampling) the differences between the performance of the models trained with and without ion-transfer augmentation are within  ≈ 0.03. This behavior is expected because the augmentation does not introduce novel binding sites but adds near-duplicate binding environments with slightly perturbed conformations, so it acts primarily as a label-consistency and regularization mechanism rather than as a source of new information (Supplementary Fig. 3 shows a modest but favorable trend for data augmentation). Importantly, the collected dataset is imbalanced with respect to both ion types and the binding sites for a given ion type. For example, there are 5203 Zn^2+^ binding sites in our dataset corresponding to 517 clusters, and the largest cluster has 480 binding sites. Similar long-tailed cluster-size distributions are observed for all 14 specific ion types considered in this work (see Supplementary Table 5, Supplementary Fig. 4). If complexes are sampled uniformly during training, the largest cluster would likely dominate over the small clusters resulting in potential overfitting towards the representative binding pattern of the largest cluster. To mitigate this, we employed a hierarchical cluster-balanced sampling scheme that equalizes the number of updates per ion class and per binding site cluster during training (Section Training). In five-fold cross-validation, this sampling strategy yields a slight improvement in mean weighted AP compared to the uniform sampling (see Supplementary Table 4 e.g., 0.545 ± 0.016 versus 0.537 ± 0.022 (mean  ± s.d. over *n* = 5 cross-validation folds) for the multi-task model trained with augmentation). We therefore used the model trained using a cluster-based sampling strategy and an augmented dataset as the final BiteNet_I_ model.

### Benchmarking against existing methods

**Fig. 2:**
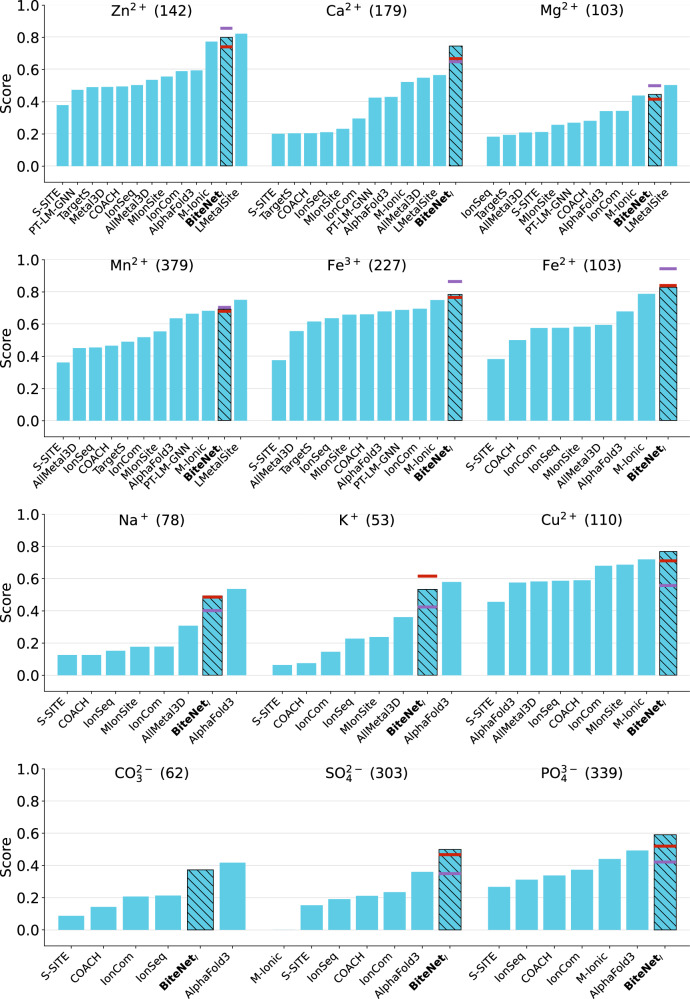
Comparison of different ion binding site prediction methods on the IonCom benchmark. Values in parentheses above each panel indicate the number of benchmark structures for the corresponding ion class (*n*). Scores (the MCC performance metric) are provided only for ions with more than 50 representative structures in the benchmark. Red horizontal lines represent scores on the IonCom validation set of the BiteNet_I_ model, trained on the BiteNet_I_ train set. The violet horizontal line represents the weighted MCC score of the BiteNet_I_ model on the test set.

We evaluated BiteNet_I_ against state-of-the-art ion-binding predictors using two widely adopted benchmarks: MIonSite^17^ and IonCom/IonSeq^18^. To ensure fair comparisons, we trained separate BiteNet_I_ models on each benchmark’s respective training data. For the MIonSite benchmark, we trained a model on the training set, calculated optimal parameters for residue scoring (see Equation (10)) with respect to MCC, and used these to compute residue scores on the validation set. For the IonCom benchmark, we performed 5-fold cross-validation; similarly, we obtained optimal parameters for residue scoring on each training set in the splits with respect to MCC and computed residue scores on the remaining validation sets. We used the averaged score over the five models as the final score for each ion class. Across both benchmarks, BiteNet_I_ consistently outperformed all competing methods for the majority of ions (see Fig. 2; Supplementary Tables 6–7 and Supplementary Fig. 5 for more details). Remarkably, BiteNet_I_ achieved two- to three-fold higher MCC values compared to most of the methods for monoatomic ions (Ca^2+^, Na^+^, K^+^) as well as for polyatomic ions (${{{{\rm{PO}}}}}_{4}^{3-}$, ${{{{\rm{SO}}}}}_{4}^{2-}\, , {{{{\rm{CO}}}}}_{3}^{2-}$), where the coordination geometry may involve backbone atoms and lack of specific residue type patterns. Although less dominant, BiteNet_I_’s performance remained competitive across other cations and anions. On the MIonSite benchmark, BiteNet_I_ matched or exceeded the sequence-based method LMetalSite for all four metal types: Ca^2+^, Mg^2+^, Zn^2+^ and Mn^2+^ (MCC: Ca^2+^ 0.641 vs 0.506; Mg^2+^ 0.415 vs 0.410; Zn^2+^ 0.843 vs 0.836; Mn^2+^ 0.805 vs 0.740) (see also Supplementary Table 6); on the IonCom benchmark, BiteNet_I_ also shows better average results across these ions compared to LMetalSite, M-Ionic and the graph-based PT-LM-GNN methods (mean MCC over Ca/Mg/Zn/Mn: 0.669 vs 0.661 (LMetalSite), 0.605 (M-Ionic), and 0.459 (PT-LM-GNN)) (see Supplementary Table 7). We likewise evaluated AllMetal3D on both benchmarks; while it achieved competitive performance for several ion types, BiteNet_I_ delivers larger average MCC (e.g., 0.675 vs 0.461 averaged over the IonCom ions reported for AllMetal3D; Supplementary Table 7).

In addition, we compared BiteNet_I_ to Metal3D^19^, a convolutional model trained specifically for Zn^2+^ binding site localization. We applied Metal3D to the IonCom and MIonSite Zn^2+^ validation sets and converted the predicted Zn^2+^ positions into residue-wise scores. While Metal3D showed high accuracy on both benchmarks, BiteNet_I_ demonstrated better performance (Supplementary Tables 7 and 6). To compare the models on the original Metal3D binding site test set, we ran BiteNet_I_ on the same biological assemblies and used the evaluation protocol of Dürr et al.^19^, i.e., we filtered Zn^2+^ sites with fewer than two unique protein ligands within 2.8 Å and occupancy ≤0.5 and treated predictions within 5 Å of an experimental Zn^2+^ site as true positives. On the resulting set of 132 Zn^2+^ sites in 54 assemblies, Metal3D with a probability cutoff of *p* = 0.5 reached a precision of 0.76, recall of 0.68 and F1-score of 0.72, whereas BiteNet_I_ with the default score threshold of 0.5 achieved a precision 0.81, recall 0.78 and F1-score 0.80 with a comparable number of false-positive clusters. Because the Metal3D test set partially overlaps with our training data, we additionally removed 34 assemblies with sequence identity ≥0.3 or TM-score ≥0.5 to any training structure, which resulted in a non-redundant subset of 51 Zn^2+^ sites in 23 assemblies. On this subset, Metal3D (*p* = 0.5) obtained precision 0.71, recall 0.69 and F1-score 0.70, while BiteNet_I_ (threshold 0.5) reached precision 0.78, recall 0.92 and F1-score 0.85. Detailed metrics for both datasets and additional score thresholds are provided in Supplementary Tables 8 and 9. These results suggest that multi-ion predictive models may outperform single-ion ones.

With the advances in protein complex structure prediction, it is also interesting to compare BiteNet_I_ with AlphaFold3^20^ by Google DeepMind (https://deepmind.google/), a successor of AlphaFold2^21^ with enhanced capability to predict structures of molecular complexes, including protein-ion complexes. For each protein in the MIonSite and IonCom benchmarks, we provided to AlphaFold3 the amino acid sequence together with a single copy of the relevant ion as input, ran the AlphaFold3 pipeline to generate five structural models, and derived residue-level ion-binding scores from these models to compute the same performance metrics as for other methods (see Section Methods for details). BiteNet_I_ shows clear improvements over AlphaFold3 for several monoatomic ions (e.g., Ca^2+^, K^+^, Mg^2+^) and polyatomic ions (e.g., ${{{{\rm{PO}}}}}_{4}^{3-}$, ${{{{\rm{SO}}}}}_{4}^{2-}$), while AlphaFold3 performs slightly better for ${{{{\rm{CO}}}}}_{3}^{2-}$ and Na^+^. We want to emphasize, however, that comparison with AlphaFold3 should not be interpreted as a fair head-to-head benchmark. The two methods aim to solve very different computational tasks: BiteNet_I_ assumes that a protein structure is already available and predicts ion-binding sites on top of it, whereas AlphaFold3 predicts the full three-dimensional structure of the protein–ion complex directly from sequence (and ligand) information and therefore does not require an input structure. Moreover, MIonSite and IonCom benchmarks likely overlap with the training data of AlphaFold3. We further explored how BiteNet_I_ performed on the AlphaFold2-generated models, rather than experimentally determined structures. Specifically, we used models from the AlphaFold Protein Structure Database^22^ as input to BiteNet_I_. This approach also demonstrated competitive performance, but in general did not outperform BiteNet_I_ applied to experimental structures.

As for the computational speed, in our experiments, a single forward pass of the multi-task BiteNet_I_ model takes  ~ 0.31 s per orientation (see Supplementary Fig. 1) and 16.0 ± 0.5 s per 50 random orientations of a structure. This makes large-scale annotation of ion-binding sites feasible when experimental or predicted structures are available. To compare with the other methods, we evaluated runtimes elapsed on the MIonSite and IonCom benchmarks (Supplementary Fig. 1). Sequence-based models were, as expected, the fastest: M-Ionic^23^ and LMetalSite^24^ required on average 0.08 ± 0.08 s and 0.17 ± 0.41 s per protein, respectively. Among structure-based methods, the graph-based PT-LM-GNN^25^ and the voxel-based AllMetal3D^26^ were substantially more demanding, with mean runtimes of 15.9 ± 20.0 s and 45.8 ± 189.0 s per protein. To provide context, we also report the elapsed runtime of AlphaFold3 (Supplementary Fig. 1), which performs a much more complex sequence-to-structure prediction task.

Finally, we would like to note that the training datasets used across studies are, strictly speaking, distinct due to differing preprocessing pipelines, temporal or similarity cutoffs, and other factors. Furthermore, several recent approaches (e.g., LMetalSite, M-Ionic, PT-LM-GNN) leverage pretrained protein language models that were trained on large protein sequence corpora. Consequently, the MIonSite and IonCom validation sets used here may overlap with the training and/or pre-training data of some methods. Therefore, the reported numbers should not be interpreted as strictly non-redundant test-set evaluation of all the methods.

### Impact of structure resolution

The accuracy of ion-binding site prediction is inherently tied to the quality of the input protein structure. Lower-resolution structures may have spoiled coordination geometry, omit coordinating atoms or misplace bound ions. In this work, we used only high-resolution structures with a resolution of ≤2 Å to compose the datasets. However, PDB contains many more structures of lower resolution but with “resolved" ions. More precisely, including structures of ≤3 Å resolution would result in a twice larger training dataset. To quantify this in more detail, we have analyzed how the number of complexes and ion-binding sites grows with the resolution cutoff (Supplementary Fig. 6, Supplementary Table 10). Changing the cutoff from ≤2.0 to ≤2.5 Å increases the number of complexes from 12,816 to 19,114 (+49%) and the number of ion sites from 35,613 to 54,117 (+52%). A breakdown by ion type (Supplementary Table 10) shows that the ten ion types that gain the largest number of sites between 2.0 and 2.5 Å already account for  ~ 88% of all additional sites. This group is dominated by sulfate (4234 extra sites,  ~ 23% of the added sites), Ca^2+^ (2180) and Zn^2+^ (2166), and also includes Cl^−^, alkali cations, Mg^2+^, phosphate (${{{{\rm{PO}}}}}_{4}^{3-}$), Mn^2+^, acetate (ACT) and K^+^. Many of these ions correspond to crystallization additives (sulfate, phosphate, acetate) or bulk solvent ions (alkali and alkaline-earth cations and Cl^−^), whose placement can be particularly ambiguous at moderate resolution.

**Fig. 3:**
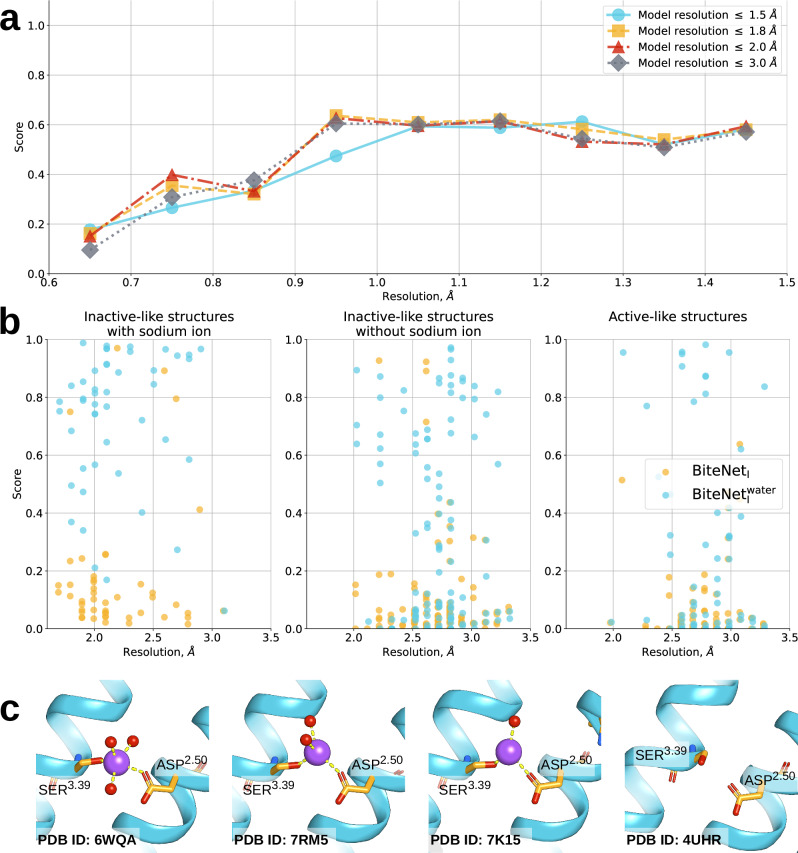
Effects of structure resolution and water molecules on BiteNet_I_ predictions. **a** The AP scores of BiteNet_I_ models as a function of experimental structure resolution. The four models were trained using structures with different resolution thresholds: ≤1.5 Å (cyan circles, solid line), ≤1.8 Å (orange squares, dashed line), ≤2.0 Å (red triangles, dash-dotted line), and ≤3.0 Å (gray diamonds, dotted line). Each point (*x*, *y*) corresponds to the AP score (*y*) calculated on the model’s validation subset of structures in the corresponding resolution bin. For each point, the exact sample size is provided in Supplementary Data 1 as the number of validation structures in the num_structures column. **b** Scatter plots with prediction scores for the sodium pocket in class A GPCR structures with at least one resolved water molecule. The three groups contain (*n* = 47) inactive-like structures with sodium ion, (*n* = 114) inactive-like structures without sodium ion, and (*n* = 71) active-like structures. Prediction scores for models trained without or with knowledge of surrounding water molecules are colored in orange and blue, respectively. **c** Examples of the sodium binding site in Class A GPCR structures with different numbers of resolved water molecules: 3 (PDB ID: 6WQA), 2 (PDB ID: 7RM5), 1 (PDB ID: 7K15), and 0 (PDB ID: 4UHR; active-like structure, no sodium). GPCR structure is represented with a cyan cartoon, water molecules are shown with red spheres, sodium ion is represented with a violet sphere, and binding residues (Asp^2.50^ and Ser^3.39^) are shown with yellow sticks.

To assess how resolution affects BiteNet_I_’s performance, we trained separate models on datasets filtered by resolution thresholds: ≤1.5 Å, ≤1.8 Å, ≤2.0 Å, and ≤3.0 Å. We did not observe that adding lower resolution structure improves the model performance on higher resolution structures, suggesting that model was not able to capture more useful information from it to generalize (see Fig. 3a and Supplementary Fig. 7). This is an expected result, because for resolution lower than 2.0 Å, the electron density may be ambiguous to place an ion, for example, reliable detection of monovalent ions typically requires a resolution of at least  < 2.3 Å^27^. Importantly, BiteNet_I_ maintained reasonable performance, suggesting robustness to structural uncertainty, making it particularly well-suited for application to predicted structures, such as those generated by AlphaFold.

While X-Ray structures are still considered more precise for atomic details, modern cryo-EM provides another source of high-resolution structures, including those with resolved ions. To test whether BiteNet_I_ transfers beyond crystallographic inputs, we assembled an external cryo-EM evaluation set from PDB by selecting entries determined by electron microscopy method, with resolution of ≤3.0 Å, and containing at least one of the ion types considered by BiteNet_I_, yielding a total of 1051 structures. We applied the pre-trained BiteNet_I_ model as is and evaluated the center-based detection performance macro-averaged across the 14 specific ion types. Notably, we observed performance on par with the held-out crystallographic test set (AP_center_ = 0.612 and AP_center_ = 0.614 for cryo-EM and X-ray structures, respectively; also see Supplementary Table 11). These results demonstrate BiteNet_I_’s robustness with respect to the learned binding site patterns and indicate its transferability to high-resolution cryo-EM structures.

Another crucial factor to consider in ion-binding site prediction is “bound” water molecules, which also require high-resolution electron density to be observed. Such water molecules frequently participate in ion coordination by forming hydrogen bonds or completing the coordination shell, particularly for monovalent and divalent cations. To quantify the influence of water on prediction accuracy, we trained two water-aware variants of BiteNet_I_ that included crystallographic water molecules in the input voxel grids. Note that in our representation each heavy atom contributes only a Gaussian density parameterized by its van der Waals radius (Eq. (1)), so we do not explicitly encode hydrogens, electrostatic effects or interactions. In the first water-aware variant, oxygen atoms of crystallographic waters were assigned to the same channel as protein hydroxyl oxygens (generic polar-oxygen channel). In the second variant, water oxygens were mapped to a dedicated “water oxygen” channel, allowing the network to distinguish them from protein side-chain oxygens. Both encodings yielded very similar performance (Supplementary Table 12); specifically, the separate water-oxygen channel model achieved a mean weighted average precision (mwAP) of 0.70 ± 0.02 (mean ± s.d. over *n* = 5 cross-validation folds), showing a solid improvement compared to 0.58 ± 0.02 mwAP of the water-free model trained without augmentation. This result highlights the critical role of solvent in ion recognition; however, it also underscores a practical limitation: in many protein structures (let alone structural models), particularly those solved at lower resolution, functionally important water molecules are absent. Last but not least, water molecules may compete with ions for occupancy, further complicating recognition of ion-binding sites. Note that all benchmark results in the previous subsections (MIonSite and IonCom) are obtained with the water-free BiteNet_I_ model; the water-aware variants are used only in this subsection and in the GPCR sodium case study below.

To demonstrate the interplay among structural resolution, conformational specificity, and “bound” water molecules, we used a sodium-binding site in G protein-coupled receptors (GPCRs) as a case study. GPCRs constitute a major class of membrane proteins with essential signaling functions and are prominent drug targets^28^. Class A GPCRs, in particular, feature a conserved sodium-binding pocket located at the center of the seven-transmembrane (7TM) helical bundle^7^. This site plays a known allosteric role^29^, preferentially stabilizing the inactive receptor conformation of a receptor. However, its identification has historically been hindered by the limited availability of high-resolution GPCR structures^30^. For fair assessment of BiteNet_I_’s capability to detect allosteric sodium binding sites in GPCRs, we removed all GPCR structures from the training set and derived a new model with no prior knowledge of sodium ions in GPCRs. We retrieved all available crystallographic structures of class A GPCRs, checked the presence of sodium ions, and assigned the structure as “active" or “inactive” according to the GPCRdb database^31^. The latter is important because sodium ions presumably stabilize the inactive-like conformations. When applying BiteNet_I_ to these structures, we observed that sodium ion is detected with high scores only in some structures, while for most of the structures it yielded low-confidence predictions (see Fig. 3b). It is known that the sodium coordination shell includes five oxygen atoms, and in GPCRs, water molecules participate in sodium coordination (see Fig. 3c). Upon including crystallographic water molecules in the input voxel grid via the polar-oxygen channel, BiteNet_I_’s predictions improved markedly: “inactive” structures with sodium showed sharp increases in confidence, while predictions for “inactive” and “active” structures without ion remained largely unchanged (see Supplementary Fig. 8). Note, that most of the “inactive” structures without sodium ion are low-resolution structures, which simply lack resolved water molecules. In the absence of high-resolution structures, one may try to model water molecules; however, it is still an open problem^32^. For instance, when using a state-of-the-art method, GalaxyWater-CNN^33^, we observed that the sodium pocket is always fully occupied by predicted water molecules with very high scores, leaving no space for the sodium ion. Stepping back, for a long time the sodium ion was not observed in the Class A GPCR structures; it was even thought to be occupied by a water molecule until the high-resolution structures revealed sodium^27^. This case study underscores the model’s sensitivity to subtle structural determinants of ion binding, including water-mediated coordination. Importantly, BiteNet_I_ demonstrated good predictive power without prior knowledge of protein family and its structures, indicating its utility as a general-purpose ion-binding site detection model.

Overall, these findings suggest that incorporating solvent-aware features could significantly enhance ion-binding site detection. Therefore, we anticipate that considering water molecules in ion-binding site prediction opens room for the following improvement.

### Point mutation analysis

**Fig. 4:**
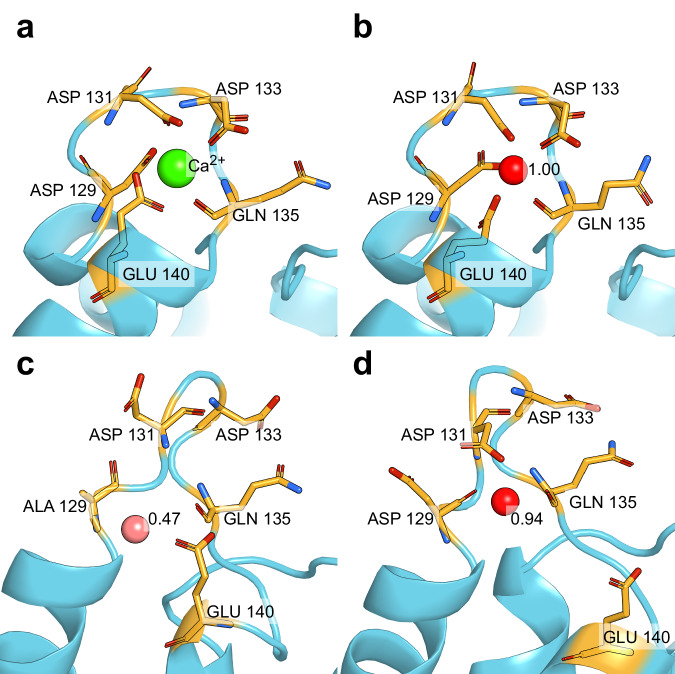
Point-mutation sensitivity of BiteNet_I_ at the calmodulin EF-hand IV Ca^2+^-binding site. All panels show the EF-hand IV loop with coordinating residues labeled. **a** Reference Ca^2+^-saturated structure (1CLL), with the experimental Ca^2+^ ion shown for context. **b** Identity-control model (D129D) generated from 1CLL (ions were removed before the generation); the predicted Ca^2+^ center (red sphere) and its BiteNet_I_ score (1.00) are shown. **c** CaM1234 structure (5TP5; D129A), the predicted Ca^2+^ (red sphere, score 0.47) corresponds to the highest-scoring NMR model. **d** Restoring Asp in the CaM1234 structure (A129D) increases the predicted Ca^2+^ site score to 0.94.

To further explore BiteNet_I_’s potential use in the point mutation analysis, we considered calmodulin (CaM), a ubiquitous Ca^2+^-sensor protein that binds up to four Ca^2+^ ions via four EF-hand motifs (helix–loop–helix Ca^2+^-binding loops), two in each lobe. According to the previous mutational studies, replacing the first coordinating Asp in each EF-hand by Ala (the CaM1234 construct) significantly disrupts Ca^2+^ binding^34^. We therefore performed in silico point mutation screening at the four positions (Asp20, Asp56, Asp93, and Asp129) in two structural backgrounds: a Ca^2+^-saturated CaM crystal structure (PDB ID: 1CLL) and the CaM1234 mutant (PDB ID: 5TP5; D20A/D56A/D93A/D129A; NMR ensemble). For each background and each position, we generated all point mutation substitutions using Modeller^35^; we remodeled (i) residues within  ± 2 positions in sequence around the mutation site and (ii) all residues within 6 Å of the mutated residue, using library_schedule=slow and md_level=slow_large. For 1CLL we generated 20 models per amino acid substitution (including the identity control D → D); for 5TP5 we started from all 20 NMR conformers and generated 20 models per conformer (400 models per amino acid substitution, including the identity control A → A). We then applied BiteNet_I_ to each structural model and retrieved the prediction scores for the binding site centers. To score each EF-hand site, we defined the site center using the coordinating atoms in 1CLL and then, for each modeled structure, assigned the score of the closest predicted Ca^2+^ center within 5 Å of this site center. In 1CLL, the four EF-hand coordination shells are formed by oxygen donors from the following residues (site I–IV, respectively): Asp20/Asp22/Asp24/Thr26/Glu31; Asp56/Asp58/Asn60/Thr62/Glu67; Asp93/Asp95/Asn97/Tyr99/Glu104; and Asp129/Asp131/Asp133/Gln135/Glu140. Across all four EF-hand sites, the point mutation screening shows that Asp is consistently among the highest-scoring substitutions in the CaM background (mean score = 0.91 ± 0.18; mean  ± s.d. over *n* = 80 modeled structures, corresponding to 4 EF-hand sites  × 20 models per site) and that replacing Asp by Ala reduces the predicted Ca^2+^ site score (0.62 ± 0.34). Conversely, in the CaM1234 background, restoring Asp increases the mean predicted site score from 0.17 to 0.23, however, the scores vary much more across the four EF-hand sites, likely due to conformational heterogeneity within the NMR ensemble (see Supplementary Fig. 9). Figure 4 illustrates these results for EF-hand IV (Asp129): the identity-control model (D129D) yields a Ca^2+^ score of 1.00, whereas the CaM1234 background yields markedly lower scores (the highest score is 0.47), and restoring Asp (A129D) results in high-scoring conformers (the highest score is 0.94). Overall, this case study suggests that BiteNet_I_ can be used as a practical tool for ranking or analysis of point mutations expected to weaken or restore ion binding.

### Ready-to-use application

To facilitate community access, we provide BiteNet_I_ as a registration-free web application at https://bitenet-ion.imolecule.app/. Users can upload protein structures in PDB format and optionally adjust two parameters: the binding score threshold and a rotational ensembling flag for improved prediction robustness (see Supplementary Fig. 10). After the job is finished, an interactive 3D viewer (via py3Dmol^36,37^) is displayed, showing predicted binding centers and scores. A downloadable CSV file lists center coordinates, ion-class predictions, probability scores, and binding-site residues. In addition, a residue-level CSV spreadsheet containing per-ion binding scores across the protein can also be downloaded. Supplementary Fig. 10 demonstrates screenshots of the landing page and the output results of the application.

To conclude, BiteNet_I_ provides a robust, multi-task framework for identifying ion-binding sites across a wide range of protein structures. It simultaneously predicts binding-site centers and residue-level binding scores for 14 biologically relevant ions using a unified 3D CNN architecture trained on high-quality experimental data. The model achieves state-of-the-art performance on established benchmarks, outperforming existing sequence- and structure-based ion-binding predictors. The freely accessible web interface and open dataset release ensure broad usability and reproducibility across the community.

## Methods

### Dataset construction

We retrieved crystallographic structures with resolution ≤2 Å from the Protein Data Bank (PDB)^38^. We filtered out non-relevant structures containing nucleic acid molecules, protein chains with less than 50 amino acid residues, containing more than eight protein chains, or exceeding 200 Å in any of the principal axes. We defined the binding site interface as a set of protein heavy atoms within 5 Å of the bound ion, and we discarded binding sites with fewer than ten atoms as non-relevant. We also removed structures in which at least one relevant ion has neighboring atoms within 5 Å from non-protein molecules of more than five atoms. This was done to eliminate cases in which ion binding occurs only in the presence of other molecules. We further curated the dataset by forming relevant ion-binding protein complexes from the crystallographic contacts to take into account binding site interfaces formed by multiple protein chains^16,39^. Additionally, we discarded complexes with more than 3 ions per chain to reduce the number of complexes with non-specific ion bindings, e.g., caused by the high concentration of ions upon crystallization. Further, we used ICM-Pro (www.molsoft.com) to refine each complex by modeling missing atoms and residues and mutating modified amino acid residues to the standard ones. We did not model gaps larger than 20 amino acids and filtered out complexes with remaining gaps or where the binding site interface size changed in more than one heavy atom. In total, we obtained a dataset of 12,816 complexes with 35,612 bound ions. In the next step, we composed a list of 123 residue names corresponding to ions and small charged molecules (see Supplementary Table 10). We chose target ion classes based on the number of different ion types in the dataset. More precisely, we selected ion types present in more than 100 different structures, provided the total number of such ions in the dataset exceeds 500. This yielded a list of 14 target ion classes, namely, Ca^2+^, Cl^−^, Co^2+^, Cu^2+^, Fe^3+^, Fe^2+^, K^+^, Mg^2+^, Mn^2+^, Na^+^, Ni^2+^, ${{{{\rm{PO}}}}}_{4}^{3-}$, ${{{{\rm{SO}}}}}_{4}^{2-}$, and Zn^2+^ (see Supplementary Table 13). After closer examination, we observed that different structures of the same protein may contain different ions in the same binding sites or even contain no ions (see Fig. 1d and Supplementary Fig. 2). This may be crucial for the derivation of a machine learning model, as the same structural region may be labeled as binding and non-binding. To avoid such a pitfall, we selected pairs of similar binding sites that are identical in terms of amino acid residues and with RMSD ≤ 1.0 Å. Then, if the ion types do not match in a pair, we generate additional complexes by superimposing the bound ion from one protein structure onto another. We further discarded cases in which the ion and protein form a clash, defined as a pair of atoms within 0.55(*r*_1_ + *r*_2_), where *r*_1_, *r*_2_ are the van der Waals radii of the atoms. Finally, we kept only those generated complexes that fulfill the curation procedure described above. Overall, the number of binding sites in the dataset increased from 35,612 to 75,647 (see Supplementary Table 13). However, this ion-transfer procedure does not introduce new binding sites: all augmented complexes share identical binding-site residues and exhibit very low RMSD values with existing structures. While in the present work we focused on X-ray crystallographic structures, for additional tests we have also considered cryo-EM structures. Supplementary Section (X-ray versus cryo-EM metal-binding sites in the PDB; Supplementary Figs. 11, 12) shows comparison of the abundance and basic geometric properties of metal-binding sites in X-ray and cryo-EM structures.

### Train-validation-test splits

To construct train-validation-test splits, we calculated sequence-, structure- and binding site-based similarities for each pair of complexes in the dataset. For the sequence identity, we first extracted sequences from all protein chains and performed multiple sequence alignment (MSA) using mafft^40^ (with parameters –retree 1 –maxiterate 0 –nofft –parttree). We defined the sequence identity as the number of non-gap characters in the MSA divided by the average length of two sequences. Similarly, for each pair of protein chains, we calculated the structure similarities using TM-align^41^ and defined the structure similarity score, as the TM-score normalized by the average length of the two chains. Finally, for each pair of ion binding sites, we calculated the binding site similarities using Probis^42^. Note that we used Probis because i) it is fast and suitable for large-scale calculations of the binding site similarities, and ii) it is based on local surface structure alignment, rather than on the cavity shape descriptors, which may be less feasible for ion binding sites. We defined the sequence, structure, and binding site similarity between two complexes as the maximum of the corresponding similarities over any pair of chains or binding sites from these complexes. Given the computed similarities, we clustered the dataset, such that two complexes belong to the same cluster if the sequence, structure or binding site similarity is higher than 0.9, 0.5 or 2.0, respectively. Then we split the obtained clusters into train-validation-test splits, so that the number of complexes in the test set is approximately 0.1 of the entire dataset. Because the splitting is done at the cluster level, no complex in the test set has sequence identity  > 0.9, TM-score  > 0.5, or binding site similarity score  > 2.0 (z-score) to any complex in the training set. Further, we clustered complexes in the train-validation partition based on sequence, structure, and binding site similarity thresholds of 0.9, 0.8, and 2.0, respectively. Finally, we used cluster-based five-fold cross-validation. Since the size of the clusters was not uniform, the largest cluster of 2537 complexes was always assigned to the train set. Further, independently for train-validation and test splits, within each ion class, we performed the following clustering: we selected all structures that contain at least one ion of this type. Then, we clustered the corresponding binding sites, and assigned the binding sites with the cluster indexes as the labels. We used the obtained labels for the cluster-based sampling during training and the calculation of the weighted metrics. Supplementary Tables 14 and 15 demonstrated the detailed statistics on the number of complexes and ions in each split.

### Test benchmarks

To quantify how well our model generalizes beyond the splits described above, we evaluated it on two widely used benchmarks—IonCom^18^ and MIonSite^17^. IonCom comprises 2100 non-redundant holo structures with ≤3.0 Å resolution, filtered to  < 40 % pairwise sequence identity and containing 3075 bound ions drawn from thirteen types (Zn^2+^, Cu^2+^, Fe^2+^, Fe^3+^, Ca^2+^, Mg^2+^, Mn^2+^, Na^+^, K^+^, ${{{{\rm{CO}}}}}_{3}^{2-}$, ${{{{\rm{NO}}}}}_{2}^{-}$, ${{{{\rm{SO}}}}}_{4}^{2-}$ and ${{{{\rm{PO}}}}}_{4}^{3-}$); binding interfaces are defined as protein heavy atoms within 5 Å of the ion, and the original study employed clustered five-fold cross-validation. MIonSite provides a training set of 7676 proteins and an identity-filtered ( < 30 %) independent test set of 274 proteins, covering twelve cations (Zn^2+^, Ca^2+^, Mg^2+^, Mn^2+^, Fe^3+^, Cu^2+^, Fe^2+^, Co^2+^, Na^+^, K^+^, Cd^2+^, and Ni^2+^); residues are labeled positive when any heavy atom lies within *r*_*i*_ + *r*_*a*_ + 0.5 Å of the ion. For both benchmarks, we report scores for BiteNet_I_ models trained (i) on the corresponding training sets from^17,18^ and (ii) on the curated training set described in Sections Dataset construction and Train-validation-test splits. Detailed metrics are presented in Section Benchmarking against existing methods, Fig. 2, Supplementary Fig. 5 and Supplementary Tables 6 and 7.

### Model

The neural network architecture is based on our previous work^15^, where we represent a protein structure as 64 × 64 × 64 voxel grids with a discretization of 1 Å and 11 channels corresponding to the different atomic types: sulfur, amide nitrogen, aromatic nitrogen, guanidinium nitrogen, ammonium nitrogen, carbonyl oxygen, hydroxyl oxygen, carboxyl oxygen, sp^2^ carbon, aromatic carbon, and sp^3^ carbon. Each channel of a voxel stores the sum of atomic occupancies calculated as:

1 $$\rho (r)=\left\{\begin{array}{ll}{e}^{-2{r}^{2}/{r}_{a}^{2}},\quad &{{{\rm{if}}}}\,r\le {r}_{{{{\rm{cutoff}}}}}\\ \!\!\!\!\!\!\!\!\!\!\!\!\!0,\quad &{{{\rm{otherwise}}}},\end{array}\right.$$

where *r* is the distance between an atom and the center of the corresponding voxel, *r*_cutoff_ = 4 Å, and *r*_*a*_ is the van der Waals radius of the atom. Note, that in the water-aware variants used in Section Impact of structure resolution, crystallographic water oxygens are additionally mapped either to the hydroxyl-oxygen channel or to a dedicated “water oxygen” channel. The voxel grids are fed to the 3D convolutional neural network that downsamples it to 16 × 16 × 16 cells of 4 voxels (see Supplementary Table 16). Each cell is linked with the output of *N*_classes_ × 4 values, where *N*_classes_ is the number of the ion classes, and 4 stands for 3D coordinates of the predicted binding site center and the probability score of the prediction. In this work, we trained the model to predict *N*_classes_ = 20 ion classes, including 14 specific ion types Ca^2+^, Cl^−^, Co^2+^, Cu^2+^, Fe^3+^, Fe^2+^, K^+^, Mg^2+^, Mn^2+^, Na^+^, Ni^2+^, ${{{{\rm{PO}}}}}_{4}^{3-}$, ${{{{\rm{SO}}}}}_{4}^{2-}$, Zn^2+^ and six generic classes corresponding to the ion charge ( − 3,  − 2,  − 1,  + 1,  + 2,  + 3) (see Supplementary Table 17). Finally, we filtered out all predictions with probability scores lower than 0.01 and performed non-maximum-suppression with the 4 Å threshold, as the post-processing stage. For the training, we used random orientation of the complexes, and for the inference, we averaged predictions obtained for 50 replicas of the input protein rotated around ten axes corresponding to the centroids of the icosahedron facets^43^ by 5 angles: *π*/3, 2*π*/3, *π*, 4*π*/3, and 5*π*/3.

### Training

We used a hierarchical sampling strategy, comprising ion-class-based and cluster-based levels, to explicitly balance ion classes and binding-site clusters during training. First, we divided the data into structures containing ions of a single class and structures containing ions of multiple classes; we denoted these two sets as *P*_class-single_ and *P*_class-multi_ and their ratio in the dataset as ${\hat{r}}_{{{{\rm{class}}}}{\mbox{-}}{{{\rm{single}}}}}$ and ${\hat{r}}_{{{{\rm{class}}}}{\mbox{-}}{{{\rm{multi}}}}}$, respectively. Next, at each sampling step, we check the ratio of the sampled structures *r*_class-single_ and *r*_class-multi_; if ${r}_{{{{\rm{class}}}}{\mbox{-}}{{{\rm{single}}}}} < {\hat{r}}_{{{{\rm{class}}}}{\mbox{-}}{{{\rm{single}}}}}$ we consider sampling from *P*_class-single_, otherwise, from *P*_class-multi_. Also, each structure is assigned a penalty, calculated as the number of times a complex was sampled multiplied by the number of ions in this complex. Given sampling from *P*_class-multi_, if not all structures are sampled at least once, we sample from it randomly; otherwise, we sample a structure with the minimum penalty. Given the sampling from *P*_class-single_, we first select the most undersampled ion class and consider structures with ions of this class clustered with respect to the ion binding site similarity. We denote *P*_site-single_ and *P*_site-multi_ as the sets of structures from *P*_class-single_ that belong to single or multiple clusters, respectively. Similarly, we calculated the ratio ${\hat{r}}_{{{{\rm{site}}}{\mbox{-}}{{{\rm{single}}}}}}$, ${\hat{r}}_{{{{\rm{site}}}{\mbox{-}}{{{\rm{multi}}}}}}$, and the penalties. Next, if ${r}_{{{{\rm{site}}}{\mbox{-}}{{{\rm{single}}}}}} < {\hat{r}}_{{{{\rm{site}}}{\mbox{-}}{{{\rm{single}}}}}}$, we consider sampling from *P*_site-single_, otherwise, from *P*_site-multi_. Finally, given a corresponding set (*P*_site-single_ or *P*_site-multi_), if not all structures are sampled, we sample a random structure from it; otherwise, we select the most undersampled cluster and sample a structure with the minimum penalty. Supplementary Fig. 13 illustrates the described sampling procedure. Note that we did not take into account non-specific ion classes (-3, -2, -1, +1, +2, +3) in the sampling procedure. It is worth noting that this hierarchical cluster-balanced sampling does not completely eliminate all sources of imbalance. In particular, individual complexes may contain multiple ions of the same or different class. Whenever such a complex is sampled, all of its ions contribute to the imbalance; therefore, complexes with multiple ions have a larger effective weight than complexes with a single ion, even under cluster-balanced sampling.

To reduce an imbalance towards negative examples, we trained models on the cubic grids containing at least one target ion using the following loss function:

2 $$L=	 {\sum}_{i=1}^{{N}_{{{{\rm{classes}}}}}}{c}_{i}\cdot \left({\sum}_{j=1}^{{N}_{{{{\rm{cells}}}}}}(-{s}_{ij}\cdot \log ({\hat{s}}_{ij})-(1-{s}_{ij})\cdot \log (1-{\hat{s}}_{ij}))\right.\\ 	 \left. +{\lambda }_{{{{\rm{coord}}}}}\cdot {\sum}_{j=1}^{{N}_{{{{\rm{cells}}}}}}{s}_{ij}\cdot ({({x}_{ij}-{\hat{x}}_{ij})}^{2}+{({y}_{ij}-{\hat{y}}_{ij})}^{2}+{({z}_{ij}-{\hat{z}}_{ij})}^{2})\right)+\gamma {L}_{2},$$

where *N*_classes_ is the number of ion classes, *N*_cells_ is the number of cells in a single output cubic grid, *s*_*ij*_ and ${\hat{s}}_{ij}$ are the true labels and the predicted probability scores, respectively, *x*_*ij*_, *y*_*ij*_, *z*_*ij*_ and ${\hat{x}}_{ij}$, ${\hat{y}}_{ij}$, ${\hat{z}}_{ij}$ are the true and predicted coordinates of the *i*-th ion class inside the *j*-th cell, respectively, *L*_2_ is the Euclidean norm of the model weights; *λ*_coord_ = 5.0 and *γ* = 10^−5^ are the weights for the coordinate mismatch and regularization terms, respectively. Note that the loss is computed only for the ion classes present in the cubic grid. We trained models for 300 epochs using the Adam optimizer, with a batch size of 16 and a learning rate decay ranging from 10^−3^ to 10^−5^.

### Metrics

We used average precision (AP; area under the precision-recall curve) as the main performance metric for validation of our model. Here, prediction is considered to be true positive (TP), if there is at least one ion of a specific class within 4 Å of the predicted center, and false positive (FP) otherwise; the false negatives (FN) are defined as the number of ions of the specific class, for which there are no predictions in the 4 Å vicinity. Precision, recall, and AP are calculated as:

3 $${{{\rm{P}}}}=\frac{{{{\rm{TP}}}}}{{{{\rm{TP}}}}+{{{\rm{FP}}}}},$$

4 $${{{\rm{R}}}}=\frac{{{{\rm{TP}}}}}{{{{\rm{TP}}}}+{{{\rm{FN}}}}},$$

5 $${{{\rm{AP}}}}={\sum}_{n}({{{{\rm{R}}}}}_{n}-{{{{\rm{R}}}}}_{n-1}){{{{\rm{P}}}}}_{n},$$

where P_*n*_ and R_*n*_ are the precision and recall calculated for the first *n* predictions, when sorted by probability score. During validation, we calculated i) the weighted average precision (wAP) for each ion class independently and ii) the mean weighted average precision (mwAP), as the average of these per-class scores. For this, to each ion, we assigned a weight, calculated as the inverse number of ions in the corresponding cluster. After that, weights of predictions are computed as the average weight of all ions of respective class in the corresponding complex, and used to calculate weighted average precision according to:

6 $${{{\rm{wP}}}}=\frac{{{{\rm{wTP}}}}}{{{{\rm{wTP}}}}+{{{\rm{wFP}}}}},$$

7 $${{{\rm{wR}}}}=\frac{{{{\rm{wTP}}}}}{{{{\rm{wTP}}}}+{{{\rm{wFN}}}}},$$

8 $${{{\rm{wAP}}}}={\sum}_{n}({{{{\rm{wR}}}}}_{n}-{{{{\rm{wR}}}}}_{n-1}){{{{\rm{wP}}}}}_{n},$$

where wTP, wFP and wFN are the weighted sums of true positive, false positive, and false negative predictions, respectively; wP_*n*_ and wR_*n*_ are precision and recall calculated for the first *n* predictions, when sorted by the probability score.

For comparison with other methods, we also calculated the residue-based metrics: average precision (AP), the area under the receiver operating characteristic curve (ROC AUC), and the Matthews correlation coefficient (MCC). MCC is defined as:

9 $${{{\rm{MCC}}}}=\frac{{{{\rm{TP}}}}\times {{{\rm{TN}}}}-{{{\rm{FP}}}}\times {{{\rm{FN}}}}}{\sqrt{({{{\rm{TP}}}}+{{{\rm{FP}}}})({{{\rm{TP}}}}+{{{\rm{FN}}}})({{{\rm{TN}}}}+{{{\rm{FP}}}})({{{\rm{TN}}}}+{{{\rm{FN}}}})}}.$$

Here, for each class, the binding residues are defined as ones within the distance *d*_*ia*_ = *r*_*i*_ + *r*_*a*_ + 0.5 from any ion from the class, where *r*_*i*_ and *r*_*a*_ are the van der Waals radii of the ion and amino acid atom, respectively. This criterion was originally proposed in the CASP experiment^44^ and was used for scoring in previous methods for prediction of ion-binding residues^17,18^. Thus, labels 1 and 0 correspond to binding and non-binding residues, and, TP and FN are the numbers of positive residues classified as binding and non-binding, respectively. Similarly, TN and FP are the numbers of negative residues classified as non-binding and binding, respectively.

As the BiteNet_I_ model outputs predictions as the centers, we perform an additional post-processing step to obtain the probability of each residue being on the binding site interface with respect to a specific ion type. For this, we scored each atom in the input protein structure according to:

10 $${s}_{ia}={{\max }_{p,\parallel {{{{\bf{r}}}}}_{p}-{{{{\bf{r}}}}}_{a}\parallel \le d}}{s}_{ip}\times {f}_{i}(\parallel {{{{\bf{r}}}}}_{ip}-{{{{\bf{r}}}}}_{a}\parallel,{r}_{{{{\rm{norm}}}}}),$$

where *i* is the ion class index, *s*_*ip*_ is the score of prediction *p* of class *i*; **r**_*ip*_ and **r**_*a*_ are the coordinates of prediction *p* for class *i* and atom *a*, respectively, *d* is the distance threshold, and *f*_*i*_ is the distance-dependent function with normalization parameter *r*_norm_. The optimal values for these parameters were chosen independently for each model and each ion class, as optimal for residue-based AP and MCC scores on the training set (see Supplementary Table 18).

We would like to stress that several common performance metrics (including MCC, accuracy, and sensitivity (recall), and specificity) are threshold-dependent. Due to the strong class imbalance (low ratio of binding residues), sensitivity and specificity can be trivially driven to extreme values by predicting almost all residues as binding or non-binding, and accuracy may appear overly optimistic because it is dominated by the majority (non-binding) class. MCC is more appropriate for imbalanced data, but it still varies with the chosen operating threshold and therefore must be interpreted together with the thresholding protocol. ROC AUC is threshold-independent, yet it can also look optimistic under severe imbalance and does not directly reflect precision at practically relevant recall levels. In contrast, the AP metric evaluates the ranking of predictions and is therefore well-suited for highly imbalanced detection tasks; we thus emphasize AP alongside MCC. Nevertheless, we report MCC, accuracy, sensitivity, specificity, and ROC AUC in the main tables. AP/ROC AUC can only be computed when per-residue prediction scores are available; hence, for some baselines, we report only threshold-based metrics as provided in the original studies.

### Reference methods used for comparison

The metrics for the reference methods reported in Fig. 2, Supplementary Fig. 5 and Supplementary Tables 6 and 7 are based on the original publications listed below or by running the authors’ released implementations as described below. For earlier methods (MetalDetector v2.0, S-SITE, COACH, TargetS, IonSeq, IonCom, MIB, MIonSite and GASS-Metal) we directly used the reported test-set performance and did not re-train or re-evaluate the models. For more recent baselines (LMetalSite, M-Ionic, PT-LM-GNN, Metal3D, AllMetal3D and AlphaFold3) we used the authors’ released implementations and pre-trained models to obtain predictions on the MIonSite and IonCom validation sets and computed the metrics in a unified way as described below. For structure-based methods (Metal3D, AllMetal3D and PT-LM-GNN), we first mapped predictions to protein atoms or residues in the reference structures and then aligned residue scores to the benchmark sequences. Structure-to-sequence mapping was performed by extracting the sequence from the PDB file and using global alignment (PARASAIL ^45^) to obtain a mapping from structure residue indices to target-sequence positions. Scores for residues that could not be aligned were set to zero. For probe-based methods (Metal3D and AllMetal3D), we assigned probes to atoms using simple distance-based cutoffs. For IonCom we used a fixed 5.0 Å cutoff between probe centers and atom positions, whereas for MIonSite we used a van-der-Waals-based cutoff, considering a probe to be in contact with an atom if the distance between the probe center and the atom was $\le 0.5+{r}_{{{{\rm{vdW}}}}}^{{{{\rm{ion}}}}}+{r}_{{{{\rm{vdW}}}}}^{{{{\rm{atom}}}}}$ Å. Atom-level scores were defined as the maximum probability over all probes contacting the atom, and residue-level scores were defined as the maximum over all atoms in the residue.

MetalDetector v2.0 (2011)^46^ is a sequence-based SVM model that classifies cysteine (CYS) and histidine (HIS) residues as binding or non-binding.

S-SITE (2013)^47^ aligns binding-specific substructures from template complexes onto the query and refines hits with sequence-profile alignment, outputting template scores for candidate sites.

COACH (2013)^47^ combines outputs from multiple methods and re-scores the aggregated pockets to rank likely binding sites.

TargetS (2013)^48^ is a template-free predictor that feeds sequence-derived windows into an ensemble of RF classifiers, then applies spatial clustering to delineate Ca^2+^, Zn^2+^, Mg^2+^, Mn^2+^ and Fe^3+^ sites.

IonSeq (2016)^18^ slides a fixed-width window along the sequence, calculates sequence-based features, and applies AdaBoost to classify residues for thirteen ion types.

IonCom (2016)^18^ fuses IonSeq scores and template matches in an SVM to rank ion-binding pockets.

MIB (2016)^49^ superposes residue triplets from the query onto a library of ion-binding triplets; clusters of compatible matches yield final predictions and docked ion poses.

MIonSite (2019)^17^ encodes sequence windows as composition and physicochemical descriptors and uses an enhanced AdaBoost ensemble to provide ligand-specific residue probabilities for twelve cations.

LMetalSite (2022)^24^ feeds protein-language model embeddings into multi-task dense layers to identify Zn^2+^, Ca^2+^, Mg^2+^ and Mn^2+^ sites without structural input. We ran the authors’ implementation with default settings and used the per-residue probabilities output for these four ions as scores for the MIonSite and IonCom sequences. More specifically, LMetalSite ^24^ is an alignment-free sequence-based metal ion-binding site predictor that uses ProtT5-XL-UniRef50 embeddings and multi-task learning to output residue-level binding probabilities for several ion types. For each input sequence the method produces a CSV file with columns of the form {ION}_prob and {ION}_pred for each supported ion, where {ION}_prob contains comma-separated probabilities (one per residue) and {ION}_pred contains comma-separated binary predictions. We ran the authors’ implementation on the MIonSite and IonCom sequences and, for each ion, extracted the corresponding {ION}_prob and {ION}_pred fields for the sequence of interest. Because LMetalSite is sequence-based, no structural alignment was required; residues are evaluated in the original sequence order. For probability-based metrics, such as AUC and average precision we used the values from {ION}_prob, whereas for binary classification metrics (accuracy, recall, specificity, F1 and MCC) we used the {ION}_pred labels provided by the method.

GASS-Meta**l** (2022)^50^ employs a genetic algorithm to optimize the superposition of local atomic environments onto a curated template set, returning sites consistent with known coordination geometries.

M-Ionic (2024)^23^ passes protein-language model residue embeddings into light gradient-boosted trees, producing ion-type-agnostic binding probabilities trained on a BioLip-derived set and evaluated on an identity-filtered hold-out set. M-Ionic^23^ is a sequence-only model that passes residue embeddings from a pretrained protein language model into gradient-boosted trees to predict metal-binding residues. In the released implementation, the model outputs binary predictions (0/1) per residue rather than continuous probabilities. We used the authors’ inference pipeline with default parameters to obtain residue-level predictions for the MIonSite and IonCom sequences. Because only binary outputs are available, probability-based metrics, such as AUC and average precision, were computed by treating the 0/1 predictions as degenerate probabilities, while all other metrics (accuracy, recall, specificity, F1 and MCC) used the binary labels directly.

AlphaFold3 (2024)^20^ For each AlphaFold3 run, we retrieved five models, and for each model, we calculated residue binding scores as follows. The residue score is zero if there is no predicted ion close to it, and the residue score is equal to the pLDDT score of the predicted ion if the distance from any heavy atom of the residue to that ion is ≤0.5 + *vdw*_1_ + *vdw*_2_ Å for MIonSite and ≤5 Å for IonCom benchmarks. After that, we average residue scores over five models. Note, that because the precise training data and temporal cut-off used to train AlphaFold3 have not been released, we did not attempt to construct a time-split benchmark with respect to its training set. Therefore, some MIonSite and IonCom complexes are likely to overlap with structures seen during AlphaFold3 training, and the reported AlphaFold3 scores should be interpreted with caution. We also checked the performance of combining of AlphaFold2^21^ model and BiteNet_I_. For this we retrieved AlphaFold2 models for proteins with PDB IDs from MIonSite and IonCom benchmarks from AlphaFold2 database^22^. After that we ran BiteNet_I_ model on these models and retrieved the residue scores.

Metal3D (2023)^19^ Metal3D^19^ is a convolutional neural network that predicts three-dimensional probability maps for Zn^2+^ positions on a voxel grid around a protein structure. We used the authors’ implementation and followed their evaluation protocol for the Zn^2+^ binding site test set. Briefly, Zn^2+^ sites with fewer than two unique protein ligands within 2.8 Å and occupancy ≤0.5 were removed from the ground truth, and a predicted Zn^2+^ site was counted as a true positive if it was within 5 Å of an experimental site. False-positive predictions were clustered with a 5 Å radius and counted once per cluster. For comparison with residue-level benchmarks (IonCom and MIonSite), we converted Metal3D Zn^2+^ site predictions to residue-wise scores using the following approach. The residue score is zero if there is no predicted ion close to it, and the residue score is equal to the Metal3D center score of the predicted ion if the distance from any heavy atom of the residue to that ion is ≤0.5 + *vdw*_1_ + *vdw*_2_ Å for MIonSite and ≤5 Å for IonCom benchmarks.

For IonCom and MIonSite, we used the Metal3D probe PDB files as input. Each probe is represented by a pseudo-atom with its occupancy field encoding the predicted probability. We treated these probes as point predictions of Zn^2+^ positions and converted them to residue-wise scores using the distance-based mapping: probes were assigned to nearby atoms using the IonCom or MIonSite interface definition, atom scores were defined as the maximum probability over assigned probes, and residue scores were defined as the maximum over atoms in the residue. Residues without any assigned probe received a score of zero, and the resulting residue-wise scores were aligned to the benchmark sequences for metric computation.

PT-LM-GNN^25^ is a hybrid method that combines ProtTrans-style protein language model embeddings with a graph neural network operating on residue contact graphs to predict ligand-binding residues. For each protein chain the public implementation outputs a pickle file containing a dictionary with two arrays: "probabilities" (residue-level probabilities) and "predictions" (residue-level binary labels). We ran the authors’ pretrained models on the MIonSite and IonCom structures for the supported ion types (Ca^2+^, Fe^3+^, Mg^2+^, Mn^2+^, Zn^2+^). For each structure, we built the required residue graph from the PDB coordinates, obtained the "probabilities" and "predictions" arrays, and mapped them to the benchmark sequences using the structure-to-sequence alignment procedure described above. For probability-based metrics (AUC, average precision) we used the "probabilities" values, while for binary metrics (accuracy, recall, specificity, F1 and MCC) we used the "predictions" labels as provided by the model, without applying any additional threshold. If the aligned prediction length did not match the target-sequence length, we treated the case as a length mismatch and used an all-zero score vector. For a subset of proteins, the PT-LM-GNN pipeline failed for technical reasons; these proteins were assigned zero scores for all residues when computing aggregate statistics.

For a small number of structures, the public implementation failed; in these cases, we assigned zero residue probabilities for metric aggregation.

AllMetal3D^26^ extends Metal3D to predict binding sites for multiple metal ion types and water molecules using 3D CNNs with the same backbone architecture as Metal3D but trained on a broader set of ions. For each protein chain, the AllMetal3D pipeline outputs a PDB file with probe centers and occupancies (equivalent to probabilities), and, in addition, a result dictionary that contains identity-class probabilities for generic “X” probes. Probe PDB files contain pseudo-atoms whose element field can either encode a specific ion (e.g., ZN, MG, CA, NA, CU) or the generic “X” probe type. The result dictionary provides class probabilities over six identity classes (["Alkali", "MG", "CA", "ZN", "NonZNTM", "NoMetal"]) for each X probe. We mapped these classes to individual ions as follows: Alkali  → Na^+^ and K^+^, NonZNTM  → Cu^2+^, Fe^3+^, Co^2+^, Mn^2+^, Ni^2+^ and Fe^2+^, and NoMetal $\to \,{{{{\rm{CO}}}}}_{3}^{2-}$, ${{{{\rm{PO}}}}}_{4}^{3-}$, ${{{{\rm{SO}}}}}_{4}^{2-}$ and ${{{{\rm{NO}}}}}_{2}^{-}$. For a given ion type we selected probes according to simple rules: (i) for ions with explicit probe elements (e.g. ZN, MG, CA) we kept probes whose element name matched the ion; (ii) for alkali ions (Na^+^, K^+^) we kept NA probes and X probes with Alkali class probability  > 0.01; (iii) for other transition-metal ions (Cu^2+^, Fe^3+^, Co^2+^, Mn^2+^, Ni^2+^, Fe^2+^) we kept CU probes and X probes with NonZNTM class probability  > 0.01; (iv) for polyatomic ions (${{{{\rm{CO}}}}}_{3}^{2-}$, ${{{{\rm{PO}}}}}_{4}^{3-}$, ${{{{\rm{SO}}}}}_{4}^{2-}$, ${{{{\rm{NO}}}}}_{2}^{-}$) we used only X probes with NoMetal class probability  > 0.01. For explicit-ion probes, we used the PDB occupancy directly as the probe score. For X probes, we multiplied the occupancy by the relevant identity-class probability from the result dictionary to obtain an ion-specific probe score. These ion-specific probe scores were then converted to atom- and residue-level scores using the same interface and aggregation strategy as for Metal3D (constant 5.0 Å cutoff for IonCom and van-der-Waals-based cutoff for MIonSite), followed by alignment to the benchmark sequences. For a small number of structures, the AllMetal3D code failed to produce predictions; in these cases, we used all-zero residue score vectors when computing aggregate metrics.

As for PT-LM-GNN, some proteins could not be processed successfully by the public implementation; these cases were assigned zero residue probabilities when computing aggregate benchmark statistics.

### Statistics and reproducibility

Model performance was evaluated using cluster-based train-validation-test splits. Cross-validation results are reported over *n* = 5 folds, where each fold corresponds to an independently trained model evaluated on a held-out validation subset defined at the cluster level. Summary values for cross-validation and ablation experiments are reported as mean ± s.d. over the five folds. Results on the independent test set were calculated once on the held-out test structures and are therefore reported without cross-validation error bars. Benchmark comparisons on MIonSite and IonCom used the sample sizes defined by the corresponding benchmark datasets or validation folds, as reported in the figure captions and Supplementary Tables. The main center-based performance metric was average precision (AP), calculated from the ranked list of predicted ion-binding centers. Weighted AP and mean weighted AP were calculated using inverse binding-site-cluster-size weights to reduce the influence of redundant binding-site clusters. For residue-level comparisons with other methods, we additionally calculated AP, ROC AUC and Matthews correlation coefficient (MCC), using the residue labels and thresholds defined in Section Metrics. No formal hypothesis tests were used; the reported comparisons are based on the performance metrics. Unless stated otherwise, sample sizes denote numbers of protein structures, ion-binding sites, generated structural models, residue-level labels, or prediction jobs, as specified in the corresponding figure caption, Supplementary Table, or Source Data file.

## Supplementary information

Transparent Peer Review file

Supplementary Information

Description of Additional Supplementary files

Supplementary Data 1

nr-reporting-summary

## Data Availability

The crystallographic and cryo-EM structures used in this study were obtained from the Protein Data Bank^38^. The PDB accession codes used for dataset construction, train-validation-test split assignments, ion annotations, processed structures, and source data underlying the figures and tables, are deposited in Zenodo under accession DOI: 10.5281/zenodo.20663378^51^. Source data for figures in the main text are also provided as Supplementary Data 1.

## Code availability

The code is available at https://github.com/i-Molecule/BiteNetIon. Software dependencies, package versions, and license information are provided in the core repository. The archived version of the code is also available in Zenodo under DOI: 10.5281/zenodo.20664508^52^. The derived BiteNet_I_ models are also freely accessible for the academic community for off-the-shelf usage at https://bitenet-ion.imolecule.app/.
