# Supplementary material for: Multivalent ion binding site identification with structure-based deep learning
Source: Commun Biol. 2026 Jul 27;9:1039. doi: 10.1038/s42003-026-10659-1 (PMC13434133; doi:10.1038/s42003-026-10659-1)
Supplement: Supplementary file 1 [file 42003_2026_10659_MOESM1_ESM.pdf]

nature portfolio

Peer Review File

Multivalent ion binding site identification with structure-based deep learning

Corresponding Author: Dr Petr Popov

This file contains all reviewer reports in order by version, followed by all author rebuttals in order by version.

Version 0:

Reviewer comments:

Reviewer #1

(Remarks to the Author)
The manuscript COMMSBIO-25-9009-T entitled “Multivalent ion binding site identification with structure-based deep learning” is an interesting and concise manuscript that described a model named BiteNetI, that uses 3-D convolutional neural networks (CNN) to localize ion binding centers and predict binding residues for 14 biologically relevant ions.

This work is based on the authors’ previous knowledge and studies combined with structure-based deep learning method, and the authors showed and claimed that their model outperforms most current methods, including AlphaFold3. I think the work deserves publication after some revision.

I have the following concerns and suggestions for their revision:

1) The authors have addressed the resolution dependence, and finally used 2.0 A as a cutoff to filter out their learning set of complex structures, I suggest that they make a histogram of the resolution dependence of their selected numbers of structures, say 1.5A, 1.8A 2.0 A, 2.3 A, 2.5 A, 2.8A and 3.0 A etc. I would suggest that a well refined model of crystallographic resolution of 2.5 A as a cutoff is more pertinent and may include more structures for the learning dataset.

2) What about a comparison with high resolution cryo-EM structures? I assume the authors have exclusively chosen crystal structures for the learning dataset, but there are more and more high resolution cryo-EM structures available (e.g. better than 2.5-2.6 A), I wonder if the metal binding sites in the high-resolution cryo-EM structures are similar to the ones in the crystal structures.

3) The following reference should be cited:
Cheng Y, Wang H, Xu H, Liu Y, Ma B, Chen X, Zeng X, Wang X, Wang B, Shiau C, Ovchinnikov S, Su XD, Wang C. Co-evolution-based prediction of metal-binding sites in proteomes by machine learning. Nat Chem Biol. 2023 Jan 2. doi: 10.1038/s41589-022-01223-z.

Some minor point: please do some language and grammar check, for example
On page 8,
3 Methods 3.1 Datasets 3.1.1 Dataset construction
“… and we discarded binding sites with less than ten atoms as…” should be “few than” etc.

Reviewer #2

(Remarks to the Author)
Reviewer Report:

The work addresses an important problem in computational structural biology, and the development of BiteNetI represents a valuable contribution to the field. The manuscript demonstrates considerable experience and effort in model development and benchmarking. However, several fundamental issues require careful attention before the work can be accepted for

publication.

Strengths of the Manuscript:
- Accurate prediction of ion binding sites is crucial for understanding metalloproteins and enzyme mechanisms.
- The authors have assembled substantial datasets and performed extensive comparisons.
- Using 3D structural information is appropriate for this problem, given the geometric constraints of ion coordination.
- Development of a web server will make this tool accessible to the broader community.

Major Concerns Requiring Revision:
1) Water Molecule Representation:
The manuscript states that water molecules are "encoded as hydroxyl oxygen atoms." This representation is chemically problematic and may introduce systematic biases into your model.

H_2O (water) and OH^- (hydroxide) possess fundamentally different electronic structures. Water is a neutral dipolar molecule, while hydroxide carries a formal -1 charge with substantially different electron density distribution. Additionally, their van der Waals radii differ significantly.

Hydrogen bonding patterns are distinct. Water has two donor hydrogens and two acceptor lone pairs, while OH^- has only one hydrogen and enhanced basicity.

Recommendation:
- Labelling the water molecules using their correct chemical identity, such as appropriate van der Waals parameters, partial charges.
- Alternatively, provide a theoretical justification for why this approximation is valid for your specific application
- Discuss potential systematic biases this choice may introduce, particularly for water-coordinated metal sites

2) AlphaFold3 Comparison Methodology
The comparison with AlphaFold3 is problematic as it compares fundamentally different computational tasks, and the acknowledged data leakage concern further undermines the fairness of this comparison.
You acknowledge that AlphaFold3 "likely was trained on the protein-ion complexes from the benchmarks." This creates a scenario where one method may have memorized test cases, making performance comparisons problematic.
Recommendations:
- Either using a time-split benchmark (structures released after AlphaFold3 training cutoff) or clearly stating this limitation and avoiding definitive superiority claims.

Time Comparisons:
The timing comparison of BiteNetI versus AlphaFold3 is unfair due to the fact that BiteNetI performs a binding site identification task on a given structure, while AlphaFold3 performs a de novo structure prediction together with other ions and ligands.

Recommendations:
- Remove or substantially revise the time comparison, acknowledging that these are fundamentally different computational tasks
- Focus comparisons on methods which are performing on equivalent tasks

Minor Issues / Recommendations:
Web Server URL:
- The web server URL is listed as "???", which appears to be a placeholder. The link should be provided.

Section: 3.2 Model Description
There appears to be a discrepancy in the ion list. The text mentions Mn^{2+} ions, but the list in Section 3.2 seems incomplete or inconsistent.
" ... including 14 specific ion types $Ca2^+$, Cl^-, Co^{2+}, Cu^{2+}, Fe^{3+}, Fe^{2+}, K^+, Mg^{2+}, Na^+, $Ni2^+$, $PO3^{-4}$, $SO_2{}^{-4}$, Zn^{2+} and ..."
This part includes 13 ions.

Generalization Testing:
To demonstrate that your model learns chemical principles rather than memorizing structural motifs, consider testing on:
- Protein folds absent from training data
- Engineered proteins with non-natural binding motifs
- Point mutations in known binding sites (does the model correctly predict loss/gain of binding?)

This would provide valuable insight into what the model has learned.

Error Analysis:
- Consider adding an analysis of failed cases (which ions are hardest to predict? which coordination geometries are more vulnerable? Which protein families?)
- Include case studies of incorrect predictions with a discussion of why the model struggled

Conclusion

This manuscript addresses an important problem in computational structural biology with a well-designed approach. BiteNetI demonstrates promising performance and represents a substantial effort in dataset curation and model development.
However, the fundamental issues that were outlined above, particularly the chemical representation of water molecules and the methodological concerns regarding AlphaFold3 comparisons, require careful attention before publication. These revisions are necessary to ensure the scientific rigor and reproducibility of the work.
I believe that addressing these concerns will greatly strengthen the manuscript and enhance its impact on the community, and will leave no gap for possible upcoming models. The core methodology is sound, and with the suggested modifications, this work has the potential to become a valuable tool for ion binding site prediction in structural biology.
I appreciate the authors' substantial efforts in developing BiteNetI and look forward to seeing the revised manuscript.

Reviewer #3

(Remarks to the Author)
=== Review Summary ===

The manuscript presents BiteNetI, a 3D Convolutional Neural Network (CNN) for predicting ion binding sites. In its current form, the paper suffers from several fundamental flaws. The core issues that need to be addressed are two-fold:

1) Unsupported claims of methodological innovation: The paper's primary methodological contributions (e.g., data augmentation) are not substantiated with sufficient empirical evidence. Since the architecture used in the paper has been used before [1], it seems that the paper does not present any true methodological novelty.

2) Inadequate benchmarking: The comparison to state-of-the-art methods is incomplete and omits key methods and metrics, making it difficult to see if the proposed method achieves any meaningful advantage over methods already available in the literature.

=== Major Concerns ===

1) Unsupported claims of methodological innovation
The paper lacks a clear focus on its specific methodological innovation. It relies on a previously used 3D-CNN based architecture [1], and the novel contributions it does claim are not sufficiently empirically supported.

1.1) Data augmentation: This is claimed as a key contribution (as it is mentioned in the abstract), yet the paper provides no direct evidence (e.g., an ablation study) demonstrating that this technique improves performance. On the contrary, the Supplementary Table S3 seems to suggest that the model trained on the augmented data performs consistently worse than on the original data. Moreover, it seems that the paper contains an incorrect formulation in the third paragraph of Section 2.1: “we did not observe performance improvement due to such augmentation in terms of mAP on five-fold cross-validation (0.545 ±0.016 vs. 0.544 ±0.015)”—those numbers seem to correspond to the first row of Supplementary Table S3 in which they seem to be defined as performance of the same model on original and augmented structures, respectively, whereas the description seems to suggest it is performance on the same test data of two models trained, respectively, without and with data augmentation.
To claim an innovative data augmentation technique, the authors must either provide rigorous evidence of its positive effect (e.g., comparing performance with and without augmentation) or do not state this as a primary contribution. (Ideal case would be to retrain at least one other structure-based method with and without the proposed augmentation to truly strengthen this claim).

1.2) Data sampling: The proposed data sampling technique is described in a unclear manner, e.g., in the first mention of the sampling procedure:
“For example, there are 5,203 Zn^{2+} binding sites in our dataset corresponding to 530 clusters, and the largest cluster has 480 binding sites. Therefore, uniform sampling during training likely leads to the overfit toward the most represented binding site cluster. For this reason, we used the cluster-based sampling described in Section Training, resulting in slight improvement compared to the uniform sampling (see Supplementary Table S3). Note, however, such cluster-based sampling does not solve the imbalance problem completely, because there are structures containing multiple ions of the same or different types.”
This passage is confusing because it exclusively uses Zn^{2+} ions as an example, while the full dataset supposedly represents all ion types. This leaves the reader uncertain whether the stated numbers refer only to the Zn^{2+} subset or are illustrative of the entire dataset.
Furthermore, the final sentence ("Note, however...") introduces a limitation of the method before the method itself has been explained. This point would be better placed in Section 3.3 (Training), alongside the detailed description of the sampling procedure. This limitation, when discussed in Section 3.3, should preferably be illustrated with a specific example to clarify the exact nature of the problem.
The schematic Figure S7 could use a more descriptive caption. For example, a toy example showing how the proposed sampling method avoids oversampling overrepresented clusters could also be helpful.
Table S3 implies that while the performance gain of the proposed sampling procedure is consistent, it is also marginal, and it

might not be a sufficient standalone innovation.

1.3) Speed-up claims: The paper highlights the method's speed; however, Figure S1 indicates that the reported deployment time requires aggregating predictions over 50 orientations of the protein-ligand complex. Furthermore, a comparison is made to AlphaFold3, a co-folding method that is not specialized for ion binding prediction and is known to be significantly slower. Without a clear demonstration of superiority over other specialized models, this comparison is not sufficient to validate the contribution (see also below).

2) Inadequate benchmarking
The experimental comparison is insufficient to properly situate BiteNetI's performance against the current SOTA.

2.1) Missing SOTA baselines: The paper omits several recent and highly relevant methods, making the comparison incomplete. A thorough comparison is essential.

2.1.1) Metal3D [2]: The paper seems to assume it does not need to compare against this specialist tool, which is a missed opportunity. Testing if a generalist model (BiteNetI) can outperform a specialist model (Metal3D) on its own benchmarks (e.g., for zinc ions) could be a genuine point of innovation (i.e., showing that a multi-valence model improves ion-specific prediction), but it is currently unexplored. Moreover, while it could be argued that the reviewed work is concurrent, the preprint of AllMetal3D [3], which extends Metal3D to all types of atoms, was published in February 2025.

2.1.2) LMetalSite: This method is mentioned in the manuscript but it is absent from the main comparison plots (Figure 2). The data in Supplementary Table S5, where it is included, suggests that BiteNetI might not offer a consistent, significant improvement over LMetalSite.

2.1.3) GPred [4]: including a Graph Neural Network in the comparisons could increase the value of the paper by exposing the advantages and limitations of different architectures.
If performance numbers for key methods are not available in the literature, those methods should be run to provide a direct and fair comparison.

2.2) Metric reporting: The authors focus on MCC and mwAP in the main text. While we agree with the point about the importance of those metrics (last paragraph of Section 3.4), the supplementary material suggests that BiteNetI might be performing slightly worse on other metrics (for example in terms of accuracy in Table S5). This selective reporting, combined with the missing baselines, gives an unfavorable impression and makes it difficult to truly assess if the proposed method achieves state-of-the-art performance. For a transparent and fair comparison, the main text must contain at least a concise overview of results on all standard metrics.

2.3) AlphaFold3 comparison: While the comparison to AlphaFold3 (AF3) is interesting, AF3 was not designed or optimized for this specific task. Beating AF3 thus does not compensate for the lack of proper benchmarking of other tools in this area. The manuscript would be much stronger if it focused on comparing its performance against tools designed specifically for ion binding site prediction.

=== Positive Aspects ===

3) Despite these major flaws, the manuscript contains valuable work. In particular, the analyses focusing on the impact of PDB structure resolution and the inclusion of solvent effects are welcome additions. Also, the authors’ effort to make the model accessible through an online application is appreciated, as it indicates significant work and commitment to tool usability.

=== Recommendations ===

4) The recommendation is that the authors improve the manuscript in the following four aspects:

4.1) Provide rigorous evidence (including ablation studies) for all claims of innovation, especially the data augmentation.

4.2) Conduct a thorough benchmark against all relevant SOTA methods, including Metal3D. LMetalSite results should be fairly reported in the main text.

4.3) Provide a concise overview of results for all standard metrics (AUROC, MCC, mwAP, Accuracy) and all methods in the main paper to allow for a fair and transparent comparison. Older methods could be omitted, but to assure fairness, similar structural methods (LMetalSite, 3DMetal [2]) need to be included, even if it requires running an inference.

4.4) Refocus the manuscript to ensure clarity and rigor—each claimed contribution should be clearly stated and well supported by evidence. Introduce innovations one at a time, demonstrating their individual impact through systematic experiments or analyses. Finally, show that the combination of these validated components leads to a state-of-the-art model.

Version 1:

Reviewer comments:

Reviewer #1

(Remarks to the Author)
I am happy with the authors' responses and revision. From my point of view, it should be OK to be published now.

Reviewer #2

(Remarks to the Author)
I thank the authors for their thorough and constructive response to the first-round comments.

The revised manuscript is substantially improved, and the new additions considerably strengthen the work. The authors have addressed all major concerns. I am satisfied with the revision and support publication, pending resolution of the following minor remaining issues.

1. Residual inconsistency in GPCR case study language (Section 2.3)

The authors clarified the water representation in the general paragraph of Section 2.3.
However, the GPCR case study paragraph retains the original problematic phrasing: "Upon including crystallographic water molecules as hydroxyl oxygen atoms in the input grid..." This sentence was not updated and directly conflicts with the clarification made just paragraphs earlier. Please revise this sentence to use language consistent with the rest of the corrected Section 2.3 (e.g., "upon including crystallographic water molecules in the input voxel grid via the polar-oxygen channel...").

2. Dataset size numbers potentially confusing for readers

The Introduction states "a manually curated dataset of ~10,000 protein–ion complex structures", while the Abstract states "over 75,000 high-resolution protein–ion complexes." The distinction between the number of PDB structures (~10,000) and the number of individual ion-binding site instances (~75,000 after augmentation) is important but may confuse a first-time reader. A brief parenthetical clarification at the first occurrence of each number would improve readability.

Summary
The authors have responded comprehensively, and the core methodology, benchmarking, and generalization evidence are now of publishable quality. The points above are all minor and can be resolved in a brief final revision without re-review. I recommend acceptance contingent on these corrections.

Reviewer #3

(Remarks to the Author)
=== Review Summary ===

The authors have thoroughly and thoughtfully addressed the reviewers' feedback. The revisions substantially improve the clarity, rigor, and completeness of the manuscript. One concern remains regarding the limited empirical support for the benefits of the proposed data augmentation, but we believe this can be addressed with a minor additional revision.

=== Concerns ===

While the authors attempt to address the previous concern about the lack of evidence for the benefits of the proposed dataset by reframing it as a “label consistency” method, the paper still lacks numerical evidence that label inconsistency was a real problem in the first place. The reasoning is plausible, but the manuscript does not show even small side-by-side improvements in model generalization. Such improvements do not need to be large or consistent across all protein targets or ion types; however, the proposed innovation should be supported by quantitative evidence rather than assumptions about potential issues in existing datasets. Since the authors describe label consistency as a form of regularization, it should be possible to demonstrate improved generalization in at least some experimental settings. We therefore recommend performing additional experiments or designing new evaluation setups if necessary, to demonstrate the benefits of the proposed data augmentation and “label consistency” approach, as showing its effectiveness could significantly strengthen the paper’s impact.

=== Positive Aspects ===

The authors effectively addressed other previous major concerns regarding the claims of methodological innovation and inadequate benchmarking. Most notable improvements:

Reframed Methodological Claims:
The authors appropriately adjusted their claims regarding data augmentation. By reframing the ion-transfer augmentation as

a regularization and label-consistency mechanism rather than the primary methodological novelty, the manuscript is now more scientifically accurate.

Comprehensive Benchmarking:
We highly appreciate the massive effort put into the expanded benchmarking. Including recent state-of-the-art baselines like LMetalSite, M-Ionic, PT-LM-GNN, Metal3D, and AllMetal3D provides a much fairer and rigorous evaluation of BiteNet1's capabilities.

Specialist vs. Generalist Evaluation:
Evaluating BiteNet1 directly against the specialized Metal3D model on its own Zn2+ test set was a great addition that strongly validates the advantages of the multi-ion approach.

=== Recommendations ===

Accept after revision.

Version 2:

Reviewer comments:

Reviewer #3

(Remarks to the Author)
The authors have successfully addressed our final concern. We appreciate the effort put into conducting this additional analysis. While the empirical improvements demonstrated are modest, the addition of Supplementary Figure S1 makes the benefits of the data augmentation visible and understandable to the reader, which is important for methodological transparency. We are satisfied with the revised manuscript.

Open Access This Peer Review File is licensed under a Creative Commons Attribution 4.0 International License, which permits use, sharing, adaptation, distribution and reproduction in any medium or format, as long as you give appropriate credit to the original author(s) and the source, provide a link to the Creative Commons license, and indicate if changes were made.
In cases where reviewers are anonymous, credit should be given to 'Anonymous Referee' and the source.
The images or other third party material in this Peer Review File are included in the article's Creative Commons license, unless indicated otherwise in a credit line to the material. If material is not included in the article's Creative Commons license and your intended use is not permitted by statutory regulation or exceeds the permitted use, you will need to obtain permission directly from the copyright holder.
To view a copy of this license, visit https://creativecommons.org/licenses/by/4.0/

MULTIVALENT ION BINDING SITE IDENTIFICATION WITH STRUCTURE-BASED DEEP LEARNING

February 18, 2026

We sincerely thank the editor and reviewers for their valuable feedback and constructive suggestions, that helped us to substantially improve the clarity and quality of the manuscript. Below we have addressed all the comments in blue and highlighted changes in the manuscript in red.

Reviewer 1

The manuscript COMMSBIO-25-9009-T entitled "Multivalent ion binding site identification with structure-based deep learning" is an interesting and concise manuscript that described a model named BiteNetI, that uses 3-D convolutional neural networks (CNN) to localize ion binding centers and predict binding residues for 14 biologically relevant ions.

This work is based on the authors' previous knowledge and studies combined with structure-based deep learning method, and the authors showed and claimed that their model outperforms most current methods, including AlphaFold3. I think the work deserves publication after some revision.

I have the following concerns and suggestions for their revision:

1) The authors have addressed the resolution dependence, and finally used 2.0 A as a cutoff to filter out their learning set of complex structures, I suggest that they make a histogram of the resolution dependence of their selected numbers of structures, say 1.5A, 1.8A 2.0 A, 2.3 A, 2.5 A, 2.8A and 3.0 A etc. I would suggest that a well refined model of crystallographic resolution of 2.5 A as a cutoff is more pertinent and may include more structures for the learning dataset.

We thank the reviewer for this constructive suggestion. In the revised Supplementary Information we now provide a dedicated figure (Figure S8) and table (Table S9) that quantify how the number of complexes and ion-binding sites in our curated dataset depends on the maximal resolution cutoff (1.5, 1.8, 2.0, 2.3, 2.5, 2.8 and 3.0 Å). Relaxing the cutoff from $\leq$ 2.0 to $\leq$ 2.5 Å increases the number of complexes from 12,816 to 19,114 (+49%) and the number of ion sites from 35,613 to 54,117 (+52%). Thus, a 2.5 Å cutoff would indeed yield a substantially larger learning dataset. A breakdown by ion type (Table S10) shows, however, that the additional 18,504 sites between 2.0 and 2.5 Å are strongly concentrated in a small set of very abundant ions. The ten ion types that gain the most sites in this interval together account for approximately 88% of all added sites. This group is dominated by sulfate (4,234 extra sites, $\sim$ 23% of the added sites), Ca^{2+} (2,180) and Zn^{2+} (2,166), and also includes Cl^-, alkali cations, Mg^{2+}, phosphate (PO_4^{3-}), Mn^{2+}, acetate (ACT) and K^+. Many of these ions correspond to crystallization additives (sulfate, phosphate, acetate) or bulk solvent ions (alkali and alkaline-earth cations and Cl^-), for which the distinction from water or other anions becomes increasingly ambiguous at moderate resolution. At the same time, our resolution-dependence experiments (Section 2.3 Impact of structure resolution, Figure 4a and Figure S4) show that including lower-resolution structures up to 3.0 Å in the training data does not improve, and in some ion classes slightly degrades, BiteNet_I performance on high-resolution validation structures. This suggests that the additional lower resolution structures could increase class imbalance and label noise, without providing a consistent benefit for generalization. For these reasons, we decided to retain a conservative cutoff of $\leq$ 2.0 Å for the main training datasets. We now explicitly document the resolution dependence of dataset size and ion composition in the Supplementary Information and clarify in the main text why we opted for the $\leq$ 2.0 Å cutoff.

Changes in the manuscript:

- Added to the main text, Impact of structure resolution (paragraph 1):
  To quantify this in more details, we have analyzed how the number of complexes and ion-binding sites grows with the resolution cutoff (Figure S8, Table S10). Changing the cutoff from ≤ 2.0 Å to ≤ 2.5 Å increases the number of complexes from 12,816 to 19,114 (+49%) and the number of ion sites from 35,613 to 54,117 (+52%). A breakdown by ion type (Table S10) shows that the ten ion types that gain the largest number of sites between 2.0 and 2.5Å already account for $\sim 88\%$ of all additional sites. This group is dominated by sulfate (4,234 extra sites, $\sim 23\%$ of the added sites), Ca^{2+} (2,180) and Zn^{2+} (2,166), and also includes Cl^-, alkali cations, Mg^{2+}, phosphate (PO_4^{3-}), Mn^{2+}, acetate (ACT) and K^+. Many of these ions correspond to crystallization additives (sulfate, phosphate, acetate) or bulk solvent ions (alkali and alkaline-earth cations and Cl^-), whose placement can be particularly ambiguous at moderate resolution.
- Added SI Figure S8
- Updated SI Table S10

2) What about a comparison with high resolution cryo-EM structures? I assume the authors have exclusively chosen crystal structures for the learning dataset, but there are more and more high resolution cryo-EM structures available (e.g. better than 2.5-2.6 A), I wonder if the metal binding sites in the high-resolution cryo-EM structures are similar to the ones in the crystal structures.

We thank the reviewer for this valuable comment; indeed it is very interesting to compare the method's performance on cryo-EM data. In the revised manuscript we now explicitly address the relationship between X-ray and cryo-EM metal-binding sites and refer to a short analysis provided in Supplementary Section X-ray versus cryo-EM metal-binding sites in the PDB (Figures S9–S10).

First, we would like to note, that despite advances in cryo-EM, the X-ray structures are still dominating, at least for the protein-ion binding site data. We analyzed all .pdb entries deposited before 1 January 2025, and counted, for each experimental method and resolution bin, how many structures contain at least one of the ion types used in this study (the same set as in Table S10). X-ray structures with at least one selected ion are much more numerous than cryo-EM structures with ions across all resolution ranges (Figure S9). For example, at nominal resolution ≤ 2.5 Å there are roughly 7.9×10^4 X-ray structures with at least one selected ion, compared to about 5.1×10^2 cryo-EM structures; at ≤ 3.0 Å these numbers are 9.3×10^4 and 2.1×10^3, respectively.

Second, strictly speaking, cryo-EM data and X-ray data may correspond to different distributions, thus, the models trained on X-ray structures may not be applicable to cryo-EM structures and vice versa. To assess whether the local geometry of metal sites is comparable, we examined two ions, Mg^{2+} and Zn^{2+}, which are the most abundant metal ions in both the X-ray and cryo-EM subsets. For each Mg^{2+} and Zn^{2+} site we collected all neighboring O/N/S atoms from protein residues, ligands and water molecules within 3 Å and computed the coordination number and the distance between the ion and the centroid of its coordinating atoms (off-centering). Overall, the distributions of mean metal–O distances are very similar between X-ray and cryo-EM structures. Mg^{2+} sites show comparable coordination numbers and off-centering in both methods, whereas Zn^{2+} sites in cryo-EM tend to have slightly fewer coordinating atoms and somewhat larger off-centering than in X-ray structures (Figure S10). These results indicate that high-resolution cryo-EM structures can provide metal-binding sites with broadly similar local geometry to X-ray crystallography, although deposited cryo-EM models are on average less complete.

Finally, we composed a set of cryo-EM structures (resolution ≤ 3.0Å; 1051 structures in total) and applied BiteNet_I to this set without any retraining. Importantly, we observed performance on par with the held-out X-ray test set. We now report this analysis in the main text (Section 2.3 Impact of structure resolution) and provide detailed per-class/per-ion metrics, including center-based AP, in a new Table S11.

Changes in the manuscript:

- Added to the main text, Impact of structure resolution (paragraph 2):
  While X-Ray structures are still considered more precise for atomic details, modern cryo-EM provides another source of high-resolution structures, including those with resolved ions. To test whether BiteNet_I transfers beyond crystallographic inputs, we assembled an external cryo-EM evaluation set from PDB by selecting entries determined by electron microscopy method, with resolution of ≤ 3.0 Å, and containing at least one of the ion types considered by BiteNet_I, yielding a total of 1051 structures. We applied the pre-trained BiteNet_I model as is and evaluated the macro-averaged across the 14 specific ion types center-based detection performance. Notably, we observed performance on par with the held-out crystallographic test set ($AP_{center} = 0.612$ and $AP_{center} = 0.614$ for cryo-EM and X-ray structures, respectively; also see Table S11). These results demonstrate BiteNet_I's robustness with respect to the learned binding site patterns and indicate its transferability to high-resolution cryo-EM structures.

- Added to the main text: Section 3.1.1 Dataset construction:
  While in the present work we focused on X-ray crystallographic structures, for additional tests we have also considered cryo-EM structures. Supplementary Section X-ray versus cryo-EM metal-binding sites in the PDB (Figures S9–S10) shows comparison of the abundance and basic geometric properties of metal-binding sites in X-ray and cryo-EM structures.
- Added to SI: Table S11, Figure S9, Figure S10
- Added to SI New Section X-ray versus cryo-EM metal-binding sites in the PDB

3) The following reference should be cited: Cheng Y, Wang H, Xu H, Liu Y, Ma B, Chen X, Zeng X, Wang X, Wang B, Shiau C, Ovchinnikov S, Su XD, Wang C. Co-evolution-based prediction of metal-binding sites in proteomes by machine learning. Nat Chem Biol. 2023 Jan 2. doi: 10.1038/s41589-022-01223-z.

We thank the reviewer for pointing out this recent work. We have now cited Cheng *et al*. (Nat. Chem. Biol. 2023) and added to the Introduction section:
Recently, Cheng *et al*. introduced a co-evolution-based machine-learning framework (MetalNet) that predicts metal-binding residue networks directly from multiple sequence alignments at proteome scale, further illustrating the potential of data-driven approaches for large-scale metalloproteome annotation [1].

Some minor point: please do some language and grammar check, for example On page 8, 3 Methods 3.1 Datasets 3.1.1 Dataset construction "... and we discarded binding sites with less than ten atoms as..." should be "few than" etc.

We thank the reviewer for careful reading, valuable remarks, and positive feedback to our work. We did additional language check and updated the grammar.

Reviewer 2

The work addresses an important problem in computational structural biology, and the development of BiteNetI represents a valuable contribution to the field. The manuscript demonstrates considerable experience and effort in model development and benchmarking. However, several fundamental issues require careful attention before the work can be accepted for publication.

Strengths of the Manuscript: - Accurate prediction of ion binding sites is crucial for understanding metalloproteins and enzyme mechanisms. - The authors have assembled substantial datasets and performed extensive comparisons. - Using 3D structural information is appropriate for this problem, given the geometric constraints of ion coordination. - Development of a web server will make this tool accessible to the broader community.

Major Concerns Requiring Revision:

1) Water Molecule Representation: The manuscript states that water molecules are "encoded as hydroxyl oxygen atoms." This representation is chemically problematic and may introduce systematic biases into your model. H2O (water) and OH- (hydroxide) possess fundamentally different electronic structures. Water is a neutral dipolar molecule, while hydroxide carries a formal -1 charge with substantially different electron density distribution. Additionally, their van der Waals radii differ significantly. Hydrogen bonding patterns are distinct. Water has two donor hydrogens and two acceptor lone pairs, while OH- has only one hydrogen and enhanced basicity.

Recommendation: - Labelling the water molecules using their correct chemical identity, such as appropriate van der Waals parameters, partial charges. - Alternatively, provide a theoretical justification for why this approximation is valid for your specific application - Discuss potential systematic biases this choice may introduce, particularly for water-coordinated metal sites

We thank the reviewer for raising this point and agree that our original wording about water representation could be misleading. BiteNet$_I$ does not encode explicit electronic-structure information for any atom type: the voxel representation uses only heavy-atom positions and Gaussian densities parametrized by tabulated van der Waals radii (Eq. 1). Formal charges, partial charges, explicit hydrogens, or lone-pair directions are not used in the model. In the original text, the phrase "encoded as hydroxyl oxygen atoms" referred to mapping water oxygens to a generic polar-oxygen channel in this coarse-grained representation, rather than to treating H_2O as OH^- with a formal -1 charge. In the revised manuscript, we clarify this abstraction explicitly in Section 3.2 (Model) and refine the description in Section 2.3 (Impact of structure resolution). Namely, we describe two water-aware BiteNet$_I$ variants: (i) one in which crystallographic water oxygens and protein hydroxyl oxygens are in the same polar-oxygen channel, and (ii) a second variant

in which water oxygens are mapped to a separate "water oxygen" channel. Both variants use the same van der Waals radius for oxygen; no partial charges or hydrogen positions are encoded, in line with the rest of the representation.

As summarized in the revised Section 2.3 and Table S9, both variants of water encodings yield very similar performance. When structural waters are included in both training and validation, the mean weighted average precision improves from 0.58 ± 0.02 for the water-free model (trained without augmentation) to 0.69–0.70, while the per-ion AP scores differ by at most 0.01 between the "merged" and "separate water" channels. Thus, for our specific application, the dominant effect is the presence of resolved water molecules in the coordination environment rather than the precise assignment of their oxygens to the hydroxyl versus water-specific channels. We also make explicit that all benchmark results on the MIonSite and IonCom datasets are obtained with the water-free BiteNet_I model. The water-aware variants are used only in the analysis of resolution and water effects (Section 2.3). For the GPCR Na^+ case study, we emphasize in the revised text that inclusion of crystallographic waters markedly increases the predicted confidence for sodium in inactive GPCR structures.

Changes in the manuscript:

- Updated in the main text, Impact of structure resolution (paragraph 4):
  To quantify the influence of water on prediction accuracy, we trained two water-aware variants of BiteNet_I in which crystallographic water molecules were included in the input voxel grids. Note, that in our representation each heavy atom contributes only a Gaussian density parameterized by its van der Waals radius (Eq. 1), so we do not explicitly encode hydrogens, electrostatic effects or interactions. In the first water-aware variant, oxygen atoms of crystallographic waters were assigned to the same channel as protein hydroxyl oxygens (generic polar-oxygen channel). In the second variant, water oxygens were mapped to a dedicated "water oxygen" channel, allowing the network to distinguish them from protein side-chain oxygens. Both encodings yielded very similar performance (Table S9); specifically, the separate water-oxygen channel model achieved a mean weighted average precision (mwAP) of 0.70 ± 0.02, showing a solid improvement compared to 0.58 ± 0.02 mwAP of the water-free model trained without augmentation.
  and futher
  Note, that all benchmark results in the previous subsections (MIonSite and IonCom) are obtained with the water-free BiteNet_I model; the water-aware variants are used only in this subsection and in the GPCR sodium case study below.

- Added to the main text, Section 3.2 Model:
  Note, that in the water-aware variants used in Section 2.3, crystallographic water oxygens are additionally mapped either to the hydroxyl-oxygen channel or to a dedicated "water oxygen" channel.

2) AlphaFold3 Comparison Methodology The comparison with AlphaFold3 is problematic as it compares fundamentally different computational tasks, and the acknowledged data leakage concern further undermines the fairness of this comparison. You acknowledge that AlphaFold3 "likely was trained on the protein-ion complexes from the benchmarks." This creates a scenario where one method may have memorized test cases, making performance comparisons problematic.

Recommendations: - Either using a time-split benchmark (structures released after AlphaFold3 training cutoff) or clearly stating this limitation and avoiding definitive superiority claims.

We fully agree with the reviewer. Our intention to use AF3 is to demonstrate that despite advances in the protein co-folding approaches, the protein-ion binding site prediction is still unsolved, rather than to make a rigorous comparison between hardly comparable methods. In the revised manuscript we have therefore explicitly reframed the AlphaFold3 analysis as well as added comparison with dedicated methods solving the same problem as our approach:

- In Section 2.2 (Benchmarking against existing methods) we rewrote the AlphaFold3 paragraph to emphasise that BiteNet_I performs structure-based ion-binding site prediction on a given protein structure, whereas AlphaFold3 is a general-purpose end-to-end complex predictor that takes sequence (and ligands) as input and does not require an experimental structure. We now state clearly that the two methods address different computational problems and that the AF3 comparison should therefore *not* be interpreted as a strictly fair head-to-head benchmark.
- In the same section we report per-ion performance for transparency, but we no longer claim that BiteNet_I "surpasses" AlphaFold3. Instead, we state that BiteNet_I attains comparable or higher MCC values for most ions, while AlphaFold3 performs slightly better for CO_3^{2-} and Na^+.
- In Section 3.5 (Reference methods used for comparison) we added a paragraph clarifying that the exact training data and temporal cut-off used for AlphaFold3 have not been released in sufficient detail. Consequently,

we could not construct a rigorous time-split benchmark with respect to AF3, and some MIonSite and IonCom complexes are likely to have been seen during AF3 training. We explicitly note that this implies a potential data leakage from the AF3 training set into our benchmarks.

- In the abstract we now describe AF3 only as a secondary, black-box reference: we state that BiteNet_I achieves state-of-the-art performance compared to existing ion-binding predictors and that its residue-level scores are often comparable to or higher than those obtained from AlphaFold3 in this constrained usage scenario, rather than making a definitive superiority claim.
- Throughout the Results and concluding sections we now stress that our main conclusions about BiteNet_I are based on comparisons with specialized ion-binding site predictors (S-SITE, COACH, IonCom, IonSeq, MIonSite, LMetalSite, etc.), while the AF3 analysis is presented as an auxiliary, illustrative baseline.

Changes in the manuscript:

- Updated in the main text, Abstract:
  On two different test benchmarks, BiteNet_I achieves state-of-the-art performance compared to existing ion-binding predictors as well as to a more general method, AlphaFold3, when used to predict the entire structure of protein bound to ions.
- Added/updated in the main text, Benchmarks against existing methods:
  With the advances in protein complex structure prediction, it is also interesting to compare BiteNet_I with AlphaFold3 [2] by Google DeepMind (`https://deepmind.google/`), a successor of AlphaFold2 [3] with enhanced capability to predict structures of molecular complexes, including protein-ion complexes. For each protein in the MIonSite and IonCom benchmarks we provided to AlphaFold3 the amino acid sequence together with a single copy of the relevant ion as input, ran the AlphaFold3 pipeline to generate five structural models, and derived residue-level ion-binding scores from these models to compute the same performance metrics as for other methods (see Section 3 for details). BiteNet_I shows clear improvements over AlphaFold3 for several monoatomic ions (e.g. Ca^{2+}, K^{+}, Mg^{2+}) and polyatomic ions (e.g. PO_4^{3-}, SO_4^{2-}), while AlphaFold3 performs slightly better for CO_3^{2-} and Na^{+}. We want to emphasize, however, that comparison with AlphaFold3 should not be interpreted as a fair head-to-head benchmark. The two methods aim to solve very different computational tasks: BiteNet_I assumes that a protein structure is already available and predicts ion-binding sites on top of it, whereas AlphaFold3 predicts the full three-dimensional structure of the protein–ion complex directly from sequence (and ligand) information and therefore does not require an input structure. Moreover, MIonSite and IonCom benchmarks likely overlap with the training data of AlphaFold3. We further explored how BiteNet_I performed on the AlphaFold2-generated models, rather than experimentally determined structures. Specifically, we used models from the AlphaFold Protein Structure Database [4] as input to BiteNet_I. This approach also demonstrated competitive performance, but in general did not outperform BiteNet_I applied to experimental structures.
- Updated sentence in the main text, Ready-to-use application (paragraph 2):
  The model achieves state-of-the-art performance on established benchmarks, outperforming existing sequence- and structure-based ion-binding predictors.
- Added to the main text, Reference methods used for comparison (AlphaFold3):
  Note, that because the precise training data and temporal cut-off used to train AlphaFold3 have not been released, we did not attempt to construct a time-split benchmark with respect to its training set. Therefore, some MIonSite and IonCom complexes are likely to overlap with structures seen during AlphaFold3 training, and the reported AlphaFold3 scores should be interpreted with caution.

Time Comparisons: The timing comparison of BiteNetI versus AlphaFold3 is unfair due to the fact that BiteNetI performs a binding site identification task on a given structure, while AlphaFold3 performs a de novo structure prediction together with other ions and ligands.

Recommendations: - Remove or substantially revise the time comparison, acknowledging that these are fundamentally different computational tasks - Focus comparisons on methods which are performing on equivalent tasks

We agree with the reviewer that the original runtime comparison between BiteNet_I and AlphaFold3 (AF3) could be interpreted as a direct measure of algorithmic efficiency between two methods that solve different computational tasks. In the revised manuscript we have therefore done the following:

- In Section 2.2 (Benchmarking against existing methods) we removed the explicit statement that BiteNet_I is "$\sim 36\times$ faster than AlphaFold3" and no longer present AF3 runtimes as a quantitative speed-up factor over BiteNet_I.

- We now report only the absolute runtime of BiteNet_I on our hardware (16.0 ± 0.5 s per protein structure) to illustrate its suitability for large-scale annotation once a structure (experimental or predicted) is available.
- We set comparison with the specific predictors as the main focus.
- We keep AF3 runtimes only in Supplementary Figure S1 for completeness and explicitly state in the main text that these timings are to provide contextual scale.

Changes in the manuscript:

- Updated in the main text, Benchmarking against existing methods (paragraph 3):
  As for the computational speed, in our experiments a single forward pass of the multi-task BiteNet_I model takes ~ 0.31 s per orientation (see Figure S1) and 16.0 ± 0.5 s per 50 random orientations of a structure. This makes large-scale annotation of ion-binding sites feasible when experimental or predicted structures are available. To compare with the other methods, we evaluated runtimes elapsed on the MIonSite and IonCom benchmarks (Figure S1). Sequence-based models were, as expected, the fastest: M-Ionic [5] and LMetalSite [6] required on average 0.08 ± 0.08 s and 0.17 ± 0.41 s per protein, respectively. Among structure-based methods, the graph-based PT-LM-GNN [7] and the voxel-based AllMetal3D [8] were substantially more demanding, with mean runtimes of 15.9 ± 20.0 s and 45.8 ± 189.0 s per protein. To provide contextual scale, we also report the elapsed runtime of AlphaFold3 (Figure S1), which performs a much more complex sequence-to-structure prediction task.
- Updated SI Figure S1

Minor Issues / Recommendations: Web Server URL: - The web server URL is listed as "???", which appears to be a placeholder. The link should be provided.

We thank the reviewer for pointing this out. In Section 2.5 ("Ready-to-use application"), we have replaced the placeholder text "???" with the actual URL of the BiteNetI web server: `https://bitenet-ion.imolecule.app/`. The same URL is also stated in the Code availability section.

Section: 3.2 Model Description There appears to be a discrepancy in the ion list. The text mentions Mn2+ ions, but the list in Section 3.2 seems incomplete or inconsistent. "... including 14 specific ion types Ca2+, Cl-, Co2+, Cu2+, Fe3+, Fe2+, K+, Mg2+, Na+, Ni2+, PO3-4, SO2-4, Zn2+ and ..." This part includes 13 ions.

We thank the reviewer for noticing this inconsistency. BiteNet_I is indeed trained to predict 14 specific ion types. We have corrected Section 3.2 to explicitly list all 14 ion types, including Mn^{2+}, and we have also ensured that the description in Section 3.1.1 is consistent with this list.

Generalization Testing: To demonstrate that your model learns chemical principles rather than memorizing structural motifs, consider testing on: - Protein folds absent from training data - Engineered proteins with non-natural binding motifs - Point mutations in known binding sites (does the model correctly predict loss/gain of binding?)

This would provide valuable insight into what the model has learned.

We agree that generalization testing is important. In fact, our dataset splitting scheme was explicitly designed to enforce generalization beyond closely related proteins. As described in Section 3.1.2, we cluster complexes by sequence identity, global structural similarity (TM-score), and binding site similarity, and then assign entire clusters to train, validation, or test sets. Because splitting is done at the cluster level, no complex in the test set has sequence identity > 0.9, TM-score > 0.5, or highly similar binding site geometry to any training complex. We have now clarified this point in the Methods (Section 3.1.2), emphasizing that the test set therefore contains proteins with different overall folds and ion-coordination environments than those seen during training. In addition, we evaluate BiteNet_I on two independently curated benchmarks, MIonSite and IonCom (Section 2.2 and Supplementary Tables S5–S6), which use their own sequence-identity filtering and train/test splits. For these benchmarks we train separate models on the respective training sets and report performance on their held-out validation/test sets, providing further evidence that the model generalizes to new proteins beyond our in-house dataset. Finally, to more directly address fold- and family-level generalization for a specific, functionally important site, we have added a difficult case study, namely, detecting the allosteric Na^+ binding pocket in class A GPCRs (Section 2.3 and Figure 4b,c). Importantly, for this experiment we removed all GPCR structures from the training data and retrained BiteNet_I. Despite having no prior exposure to GPCR folds, the model correctly identifies the Na^+ pocket in inactive-like GPCR structures and captures its dependence on both receptor conformation and coordinating water molecules.

To further test the generalizing ability we performed evaluation of cryo-EM structures (our models trained on the crystallographic structures) - please see answer to the Reviewer 1.

We thank the reviewer for the idea about the point mutations. To see if BiteNet_I can be useful in analysis of point mutations, we have performed an in silico mutagenesis case study on a canonical Ca^{2+}-binding motif in calmodulin (CaM). CaM binds up to four Ca^{2+} ions via four EF-hand loops, and a previous mutational study reported that substituting the first coordinating Asp in each EF-hand (positions 20, 56, 93, and 129) with Ala (the CaM1234 construct) strongly disrupts Ca^{2+} binding [9]. We therefore performed in silico mutagenesis analysis in two structural backgrounds: a Ca^{2+}-bound crystal structure (PDB ID: 1CLL; WT) and the CaM1234 mutant (PDB ID: 5TP5; D20A/D56A/D93A/D129A; NMR ensemble). For each position and substitution we generated mutant structures with MODELLER [10], restricting remodeling to residues within ± 2 positions in sequence and within 6 Å of the mutation site (`library_schedule=slow`, `md_level=slow_large`). We quantified the predicted Ca^{2+} site score as the score of the closest predicted Ca^{2+} center within 5 Å of a site center defined from coordinating atoms in 1CLL. Across all four EF-hands, the WT background shows consistently high scores for the identity control (D→D; $n = 80$, mean$= 0.911$, std$= 0.184$) and a marked reduction for the disruptive Asp→Ala substitution (D→A; $n = 80$, mean$= 0.622$, std$= 0.336$). Conversely, in the CaM1234 mutant background, the identity control is low-scoring (A→A; $n = 1600$, mean$= 0.171$, std$= 0.196$), and restoring Asp increases the predicted Ca^{2+} site score (A→D; $n = 1600$, mean$= 0.225$, std$= 0.208$), with a smaller average effect due to conformational heterogeneity across the NMR ensemble. We report the full mutational scan as a heatmap in the Supplementary Information (Figure S6) and illustrate the effect visually for EF-hand IV (Asp129) in the main text (Figure 3).
We believe altogether these tests provide a solid evidence that the model has learned general coordination patterns rather than memorizing the training dataset.

Changes in the manuscript:

- Added to the main text, Train-validation-test splits:
  Because the splitting is done at the cluster level, no complex in the test set has sequence identity > 0.9, TM-score > 0.5, or binding site similarity score > 2.0 (z-score) to any complex in the training set.

- Added to the main text, Point mutation analysis:
  To further explore BiteNet_I's potential use in the point mutation analysis, we considered calmodulin (CaM), a ubiquitous Ca^{2+}-sensor protein that binds up to four Ca^{2+} ions via four EF-hand motifs (helix–loop–helix Ca^{2+}-binding loops), two in each lobe. According to the previous mutational studies, replacing the first coordinating Asp in each EF-hand by Ala (the CaM1234 construct) significantly disrupts Ca^{2+} binding [9]. We therefore performed in silico point mutation screening at the four positions (Asp20, Asp56, Asp93, and Asp129) in two structural backgrounds: a Ca^{2+}-saturated CaM crystal structure (PDB ID: 1CLL) and the CaM1234 mutant (PDB ID: 5TP5; D20A/D56A/D93A/D129A; NMR ensemble). For each background and each position we generated all point mutation substitutions using Modeller [10]; we remodeled (i) residues within ± 2 positions in sequence around the mutation site and (ii) all residues within 6 Å of the mutated residue, using `library_schedule=slow` and `md_level=slow_large`. From 1CLL we generated 20 models per amino acid substitution (including the identity control D→D); from 5TP5 we started from all 20 NMR conformers and generated 20 models per conformer (400 models per amino acid substitution, including the identity control A→A). We then applied BiteNet_I to each structural model and retrieved the prediction scores for the binding site centers. To score each EF-hand site, we defined the site center using the coordinating atoms in 1CLL and then, for each modelled structure, assigned the score of the closest predicted Ca^{2+} center within 5 Å of this site center. In 1CLL, the four EF-hand coordination shells are formed by oxygen donors from the following residues (site I–IV, respectively): Asp20/Asp22/Asp24/Thr26/Glu31; Asp56/Asp58/Asn60/Thr62/Glu67; Asp93/Asp95/Asn97/Tyr99/Glu104; and Asp129/Asp131/Asp133/Gln135/Glu140. Across all four EF-hand sites, the point mutation screening shows that Asp is consistently among the highest-scoring substitutions in the CaM background (mean score$= 0.91 \pm 0.18$) and that replacing Asp by Ala reduces the predicted Ca^{2+} site score (mean$= 0.62$, std$= 0.34$). Conversely, in the CaM1234 background, restoring Asp increases the mean predicted site score from 0.17 to 0.23, however, the scores vary much more across the four EF-hand sites, likely due to conformational heterogeneity within the NMR ensemble (see Figure S6). Figure 3 illustrates these results for EF-hand IV (Asp129) : the identity-control model (D129D) yields a Ca^{2+} score of 1.00, whereas the CaM1234 background yields markedly lower scores (the highest score is 0.47), and restoring Asp (A129D) results in high-scoring conformers (the highest score is 0.94). Overall, this case study suggests that BiteNet_I can be used as a practical tool for ranking or analysis of point mutations expected to weaken or restore ion binding.

- Added Figure 3 to the main text.

- Added Figure S6 to SI.

Error Analysis: - Consider adding an analysis of failed cases (which ions are hardest to predict? which coordination geometries are more vulnerable? Which protein families?) - Include case studies of incorrect predictions with a discussion of why the model struggled

We thank the reviewer for this suggestion. First, we have summarized per-ion difficulty for the model in Section 2.1 (Models' architecture and performance) based on Supplementary Table S2. As expected, ions with well-defined and strongly chelated coordination geometries (Zn^{2+}, Fe^{3+}/Fe^{2+}, Mn^{2+}, Co^{2+}, Cu^{2+}) achieve the highest AP and MCC scores, whereas monovalent cations and anions with more diffuse or context-dependent coordination patterns (Na^{+}, K^{+}, Cl^{-}, SO_4^{2-}, PO_4^{3-}) remain more challenging. Second, we expand our qualitative analysis of failure modes in the Results section. As shown in Figure 1c and Supplementary Table S4, a substantial fraction of high-scoring false positives correspond to charged small molecules such as nucleotide phosphates or heme groups, indicating that BiteNet_I tends to recognize generic ion-like coordination patterns even when these are occupied by non-ion cofactors. On the other hand, our resolution analysis (Figure 4a and Supplementary Figure S4) shows that model's performance degrades for lower-resolution structures, where coordination geometry and bound ions are less reliably resolved, which is also somewhat expected. As for the false negative examples, we inspected test-set structures where BiteNet_I yields zero recall for a given ion class (i.e., no prediction of the correct class within 4 Å of any ground-truth ion). We found that these cases represent ions coordinated predominantly by solvent molecules and/or crystallization additives rather than by protein atoms, as well as low-occupancy ions. We include representative examples and their coordination environments in Figure 1e.

Changes in the manuscript:

- Added to the main text, Error analysis subsection in Models' architecture and performance:
  Consistent with chemical intuition, we observed that ion types with well-defined, strongly chelated coordination geometries such as Zn^{2+}, Fe^{3+}/Fe^{2+}, Mn^{2+}, Co^{2+} and Cu^{2+} achieve the highest AP and MCC scores, whereas monovalent cations and anions with more diffuse and context-dependent coordination patterns (Na^{+}, K^{+}, Cl^{-}, SO_4^{2-}, PO_4^{3-}) remain more challenging (Table S2).
  and further
  As for the false negative examples, we inspected test-set structures for which BiteNet_I shows zero recall for a given ion class, i.e. no predicted sites of that class fall within the distance threshold (4 Å) of any annotated ion. Across the inspected cases, the missed ions frequently exhibit weak or ambiguous coordination patterns: many are predominantly solvent-coordinated (e.g. hexahydrated Mg^{2+}) or stabilized by crystallization additives such as acetate or sulfate rather than by protein side chains. We also observed ions refined with partial occupancies (e.g. Ni^{2+} in PDB ID: 4YBN with occupancy 0.20), indicating weak support for the ion assignment and/or low site population. Figure 1**e** shows representative examples of such atomic environments. Together with the resolution-dependent analysis in Section 2.3 and Figure 4, these observations indicate that false predictions are enriched in structurally ambiguous regions (low-resolution or partially resolved coordination shells) and in sites that are chemically compatible with ion binding but are occupied by other charged cofactors in a given structure.
- Updated Figure 1**d** in the main text.
- Added Figure 1**e** in the main text.

Conclusion

This manuscript addresses an important problem in computational structural biology with a well-designed approach. BiteNetI demonstrates promising performance and represents a substantial effort in dataset curation and model development. However, the fundamental issues that were outlined above, particularly the chemical representation of water molecules and the methodological concerns regarding AlphaFold3 comparisons, require careful attention before publication. These revisions are necessary to ensure the scientific rigor and reproducibility of the work. I believe that addressing these concerns will greatly strengthen the manuscript and enhance its impact on the community, and will leave no gap for possible upcoming models. The core methodology is sound, and with the suggested modifications, this work has the potential to become a valuable tool for ion binding site prediction in structural biology. I appreciate the authors' substantial efforts in developing BiteNetI and look forward to seeing the revised manuscript.

We thank the reviewer for careful reading, valuable remarks, and positive feedback to our work.

Reviewer 3

=== Review Summary ===

The manuscript presents BiteNetI, a 3D Convolutional Neural Network (CNN) for predicting ion binding sites. In its current form, the paper suffers from several fundamental flaws. The core issues that need to be addressed are two-fold:

1) Unsupported claims of methodological innovation: The paper's primary methodological contributions (e.g., data augmentation) are not substantiated with sufficient empirical evidence. Since the architecture used in the paper has been used before [1], it seems that the paper does not present any true methodological novelty.

2) Inadequate benchmarking: The comparison to state-of-the-art methods is incomplete and omits key methods and metrics, making it difficult to see if the proposed method achieves any meaningful advantage over methods already available in the literature.

=== Major Concerns ===

1) Unsupported claims of methodological innovation The paper lacks a clear focus on its specific methodological innovation. It relies on a previously used 3D-CNN based architecture [1], and the novel contributions it does claim are not sufficiently empirically supported.

1.1) Data augmentation: This is claimed as a key contribution (as it is mentioned in the abstract), yet the paper provides no direct evidence (e.g., an ablation study) demonstrating that this technique improves performance. On the contrary, the Supplementary Table S3 seems to suggest that the model trained on the augmented data performs consistently worse than on the original data. Moreover, it seems that the paper contains an incorrect formulation in the third paragraph of Section 2.1: "we did not observe performance improvement due to such augmentation in terms of mAP on five-fold cross-validation (0.545 ±0.016 vs. 0.544 ±0.015)"—those numbers seem to correspond to the first row of Supplementary Table S3 in which they seem to be defined as performance of the same model on original and augmented structures, respectively, whereas the description seems to suggest it is performance on the same test data of two models trained, respectively, without and with data augmentation. To claim an innovative data augmentation technique, the authors must either provide rigorous evidence of its positive effect (e.g., comparing performance with and without augmentation) or do not state this as a primary contribution. (Ideal case would be to retrain at least one other structure-based method with and without the proposed augmentation to truly strengthen this claim).

We thank the reviewer for careful reading of our manuscript. Indeed, the values 0.545 ± 0.016 and 0.544 ± 0.015 correspond to the first row of Supplementary Table S3 (Table S3 in the revised Supplementary Information). These numbers report the mean weighted average precision (mwAP) of the *same* multi-task model with cluster-based sampling and ion-transfer augmentation, evaluated on (i) the subset of original complexes (no added ions) and (ii) the full augmented set (with transferred ions), respectively. To clarify this, we have rewritten the paragraph in Section 2.1.2 (Ablation study) to state that these values compare evaluation on original versus augmented structure sets for a single trained model, and we now refer directly to Supplementary Table S3 when discussing these numbers. Note, that Supplementary Table S3 already contains the ablation study over: (i) model heads (multi-task vs. single-task), (ii) data augmentation (with vs. without ion transfers), and (iii) sampling strategy (cluster-based vs. random). As the reviewer points out, the best mwAP on the original, non-augmented structures is obtained for the multi-task model trained *without* ion-transfer augmentation and *with* cluster-based sampling (0.576 ± 0.023). When ion-transfer augmentation is enabled and the same sampling scheme is used, the corresponding mwAP on the original structures is lower (0.545 ± 0.016). Across all tested configurations, the differences between models trained with and without augmentation are very minor (on the order of ≈ 0.03 or smaller) and comparable to the standard deviations across the five cross-validation folds. We therefore agree that the ion-transfer augmentation does not lead to a systematic improvement in global mwAP on our internal cross-validation splits, and we explicitly state this in the revised Section 2.1.2 (Ablation study). Further to avoid impression that data augmentation is the key contribution of this work, in the revised manuscript:

- In the abstract, we no longer highlight the augmentation as a key methodological contribution. Instead, we describe the training data as "a carefully curated dataset of over 75,000 high-resolution protein–ion complexes, in which near-identical binding sites are consistently annotated by transferring ions between homologous structures".
- In Section 3.1.1 (Dataset construction), we clarify that the ion-transfer procedure does not introduce novel binding sites, but generates complexes with very similar binding environments and slightly perturbed conformations. Its main purpose is to (i) enforce consistent labels for near-identical binding sites across different PDB entries and (ii) expose the model to this additional information, as a kind of model's regularization for near-identical binding sites.
- In Section 2.1.2 (Ablation study) we discuss the ablation study in Table S3 noting that the best mwAP is achieved without augmentation.

1.2) Data sampling: The proposed data sampling technique is described in a unclear manner, e.g., in the first mention of the sampling procedure: "For example, there are 5,203 Zn2+ binding sites in our dataset corresponding to 530 clusters, and the largest cluster has 480 binding sites. Therefore, uniform sampling during training likely leads to the overfit toward the most represented binding site cluster. For this reason, we used the cluster-based sampling described in Section Training, resulting in slight improvement compared to the uniform sampling (see Supplementary Table S3). Note, however, such cluster-based sampling does not solve the imbalance problem completely, because there are structures containing multiple ions of the same or different types." This passage is confusing because it exclusively uses Zn2+ ions as an example, while the full dataset supposedly represents all ion types. This leaves the reader uncertain whether the stated numbers refer only to the Zn2+ subset or are illustrative of the entire dataset. Furthermore, the final sentence ("Note, however...") introduces a limitation of the method before the method itself has been explained. This point would be better placed in Section 3.3 (Training), alongside the detailed description of the sampling procedure. This limitation, when discussed in Section 3.3, should preferably be illustrated with a specific example to clarify the exact nature of the problem. The schematic Figure S7 could use a more descriptive caption. For example, a toy example showing how the proposed sampling method avoids oversampling overrepresented clusters could also be helpful. Table S3 implies that while the performance gain of the proposed sampling procedure is consistent, it is also marginal, and it might not be a sufficient standalone innovation.

We agree and thank the reviewer for this helpful suggestion. In Section 2.1.2 (Ablation study) we now specify that the numbers 5,203 binding sites and 517 clusters refer specifically to Zn^{2+}, and we clarify that similar long-tailed cluster-size distributions are observed for all 14 specific ion types considered in this work (new Table S13, Figure S11).

The sentence discussing the limitation of the sampling scheme (structures containing multiple ions of different classes) has been moved from the Results section to the Training subsection (Section 3.3), where the hierarchical sampling algorithm is described. We explain that the sampler is a heuristic compromise that aim to reduce, but does not completely eliminate, cluster-level imbalance. We have expanded the caption of Figure S12 to give a step-by-step description of the hierarchical class- and cluster-based sampling procedure, clarifying the role of single- vs multi-class and single- vs multi-cluster complexes.

Changes in the manuscript:

- Updated Abstract:
  Trained on a carefully curated dataset of over 75,000 high-resolution protein–ion complexes, in which near-identical binding sites are consistently annotated by transferring ions between homologous structures, BiteNet_I shows strong generalization ability across diverse ions within a unified multitask architecture.
- Added to main text, Subsection 'Ablation study' in Models' architecture and performance:
  Note that we enlarged the dataset more than twofold by adding superimposed ions to highly similar binding sites (see Section 3.1.1). To quantify the effect of this ion-transfer augmentation and the sampling strategy on the models' performance, we conducted an ablation study (see Supplementary Table S3 for detailed results). We observed, that training without transferred ions and with cluster-based sampling yields the highest mwAP on the original, non-augmented structures for the multi-task model (0.576 ± 0.023). Adding transferred ions and training with the same sampling scheme leads to a slightly lower mwAP on the original structures (0.545 ± 0.016), and across all tested configurations (multi-task vs. single-task heads, cluster-based vs. random sampling) the differences between performance of the models trained with and without ion-transfer augmentation are within ≈ 0.03. This behavior is expected, because the augmentation does not introduce novel binding sites but adds near-duplicate binding environments with slightly perturbed conformations, so it acts primarily as a label-consistency and regularization mechanism rather than as a source of new information. Importantly, the collected dataset is imbalanced not only with respect to the ion types, but also with respect to the binding sites for a given ion type. For example, there are $5{,}203$ Zn^{2+} binding sites in our dataset corresponding to 517 clusters, and the largest cluster has 480 binding sites. Similar long-tailed cluster-size distributions are observed for all 14 specific ion types considered in this work (see Table S13, Figure S11). If complexes are sampled uniformly during training, the largest cluster would likely dominate over the small clusters resulting in potential overfitting towards representative binding pattern of the largest cluster. To mitigate this, we employed a hierarchical cluster-balanced sampling scheme that equalizes the number of updates per ion class and per binding site cluster during training (Section 3.3). In five-fold cross-validation this sampling strategy yields a slight improvement in mean weighted AP compared to the uniform sampling (see Table S3 e.g. 0.545 ± 0.016 versus 0.537 ± 0.022 for the multi-task model trained with augmentation). We therefore used the model trained using cluster-based sampling strategy and augmented dataset, as the final BiteNet_I model.
- Added to the main text, Dataset construction:
  However, this ion-transfer procedure does not introduce new binding sites: all augmented complexes share identical binding site residues and very low RMSD with existing structures.

- Updated in the main text, Training:
  We used an hierarchical sampling strategy, comprising ion class-based and cluster-based levels, to explicitly balance both ion classes and binding site clusters during training.
  and further
  It is worth to note, this hierarchical cluster-balanced sampling does not completely eliminate all sources of imbalance. In particular, individual complexes may contain multiple ions of the same or different class. Whenever such a complex is sampled, all of its ions contribute to imbalance; therefore, complexes with multiple ions have a larger effective weight than complexes with a single ion, even under cluster-balanced sampling.
- Added to Supplement: Table S13, Figure S11
- Updated Caption in Supplement Figure S12

1.3) Speed-up claims: The paper highlights the method's speed; however, Figure S1 indicates that the reported deployment time requires aggregating predictions over 50 orientations of the protein-ligand complex. Furthermore, a comparison is made to AlphaFold3, a co-folding method that is not specialized for ion binding prediction and is known to be significantly slower. Without a clear demonstration of superiority over other specialized models, this comparison is not sufficient to validate the contribution (see also below).

We agree with the reviewer. In the revised manuscript we now specify that the reported 16.0 ± 0.5 s per structure corresponds to our default deployment setting, where BiteNet$_{\mathrm{I}}$ aggregates predictions over 50 random orientations of the protein structure. We additionally report the single-orientation runtime (~ 0.31 s for the multi-task model) and clarify in the caption of Figure S1 that the bars show single-orientation timings, whereas the dashed line corresponds to the 50-orientation setting. We also softened the comparison to AlphaFold3 (also see responses for Reviewer 2), using its runtime only as contextual information rather than as a direct speed-up baseline. Finally, we have done comparison against the specialized methods and added the corresponding analysis in the manuscript.

Changes in the manuscript:

- Updated in the main text, Benchmarking against existing methods (paragraph 3):
  As for the computational speed, in our experiments a single forward pass of the multi-task BiteNet$_{\mathrm{I}}$ model takes ~ 0.31 s per orientation (see Figure S1) and 16.0 ± 0.5 s per 50 random orientations of a structure. This makes large-scale annotation of ion-binding sites feasible when experimental or predicted structures are available. To compare with the other methods, we evaluated runtimes elapsed on the MIonSite and IonCom benchmarks (Figure S1). Sequence-based models were, as expected, the fastest: M-Ionic [5] and LMetalSite [6] required on average 0.08 ± 0.08 s and 0.17 ± 0.41 s per protein, respectively. Among structure-based methods, the graph-based PT-LM-GNN [7] and the voxel-based AllMetal3D [8] were substantially more demanding, with mean runtimes of 15.9 ± 20.0 s and 45.8 ± 189.0 s per protein. To provide contextual scale, we also recorded the elapsed runtime of AlphaFold3 (Figure S1), which performs a much more complex sequence-to-structure prediction task.
- Updated in SI, Figure S1 includes performance of the other methods.

2) Inadequate benchmarking The experimental comparison is insufficient to properly situate BiteNetI's performance against the current SOTA.

2.1) Missing SOTA baselines: The paper omits several recent and highly relevant methods, making the comparison incomplete. A thorough comparison is essential.

We agree and thank the Reviewer for this important remark. In the revised manuscript we substantially extended the comparison with state-of-the-art approaches. In addition to the methods originally included in our IonCom and MIonSite comparisons, we evaluate the recent sequence-based predictors LMetalSite and M-Ionic, the hybrid graph-based method PT-LM-GNN, and the structure-based Metal3D and AllMetal3D methods. For these predictors we used the authors' public implementations and pretrained models to obtain predictions on the MIonSite and IonCom validation sets, and we converted their outputs to residue-level scores using a unified protocol described in a new Supplementary Methods subsection ("External baselines and score computation") and summarized at the end of the Methods section in the main text. The main comparison figure (Figure 2) has been updated so that LMetalSite, M-Ionic and PT-LM-GNN are shown alongside BiteNet$_{\mathrm{I}}$ on both IonCom and MIonSite. Detailed per-ion results for all methods, including Metal3D and AllMetal3D, are now provided in Supplementary Tables S5–S5 and in the newly added Zn-specific tables for IonCom and MIonSite. Across both benchmarks, BiteNet$_{\mathrm{I}}$ consistently outperforms or matches these modern baselines for the majority of ions.

Last but not least, we note in the revised manuscript that for most external methods the exact training data and temporal cut-offs relative to MIonSite and IonCom are not available in a form that would allow us to remove all possible training overlaps. Our comparisons should therefore be interpreted as representative of typical user-facing performance of these methods on standard benchmarks, rather than as strictly non-redundant test-set evaluations.

2.1.1) Metal3D [2]: The paper seems to assume it does not need to compare against this specialist tool, which is a missed opportunity. Testing if a generalist model (BiteNetI) can outperform a specialist model (Metal3D) on its own benchmarks (e.g., for zinc ions) could be a genuine point of innovation (i.e., showing that a multi-valence model improves ion-specific prediction), but it is currently unexplored. Moreover, while it could be argued that the reviewed work is concurrent, the preprint of AllMetal3D [3], which extends Metal3D to all types of atoms, was published in February 2025.

We thank the reviewer for this suggestion. In the revised manuscript we have carried out two complementary sets of experiments. First, we evaluated BiteNet_I on the original Metal3D Zn^{2+} binding site test set. We obtained the Metal3D Zn^{2+} test set from Dürr et al. [2] and ran BiteNet_I on the same biological assemblies, strictly following the Metal3D evaluation protocol: (1) Zn^{2+} sites with fewer than two unique protein ligands within 2.8 Å and occupancy ≤ 0.5 were removed from the ground truth; (2) a predicted Zn^{2+} site was counted as a true positive if its center lay within 5 Å of an experimental Zn^{2+} ion; and (3) false-positive predictions were clustered at 5 Å and counted once per cluster. On the resulting set of 132 Zn^{2+} sites in 54 assemblies, Metal3D with the default cutoff $p = 0.5$ achieved precision 0.76, recall 0.68 and F1-score 0.72, whereas BiteNet_I with its default score threshold 0.5 achieved precision 0.81, recall 0.78 and F1-score 0.80 with a comparable number of false-positive clusters. Because the Metal3D test set partially overlaps with our training data, we additionally removed assemblies with sequence identity ≥ 0.3 or TM-score ≥ 0.5 to any BiteNet_I training structure, which yielded a non-redundant subset of 51 Zn^{2+} sites in 23 assemblies. On this subset, Metal3D ($p = 0.5$) obtained precision 0.71, recall 0.69 and F1-score 0.70, whereas BiteNet_I (threshold 0.5) reached precision 0.78, recall 0.92 and F1-score 0.85. These results, together with per-threshold statistics, are now reported in Section 2.2 and in Supplementary Tables S7 (full Metal3D test set) and S8 (non-redundant subset). This directly demonstrates that the generalist multi-ion BiteNet_I model can outperform the Zn-specific Metal3D model on its own benchmark. Second, we evaluated Metal3D and the more recent AllMetal3D [8] on the IonCom and MIonSite benchmarks. For Metal3D, we used the Zn probe PDB files produced by the public implementation and converted probe centers into residue-wise scores by: (1) assigning probes to nearby heavy atoms using a fixed 5.0 Å cutoff for IonCom or a van-der-Waals-based cutoff for MIonSite; and (2) aggregating probe probabilities by taking the maximum over probes per atom and the maximum over atoms per residue. For AllMetal3D, the pipeline outputs PDB files with probes for specific ions and generic "X" probes, along with a Python result dictionary providing identity-class probabilities (Alkali, MG, CA, ZN, NonZNTM, NoMetal) for each X probe. We mapped these classes to individual ions (Na^{+}/K^{+}, transition metals, CO_3^{2-}/PO_4^{3-}/SO_4^{2-}/NO_2^{-}) and constructed ion-specific probe scores by: (i) using the PDB occupancy directly for probes with an explicit ion element (e.g. ZN, MG, CA); and (ii) multiplying the occupancy of X probes by the relevant identity-class probability from the result dictionary. These ion-specific probe scores were then converted to residue-level scores using the same distance-based assignment and aggregation strategy as for Metal3D, followed by alignment to the benchmark sequences. On IonCom and MIonSite, Metal3D shows high accuracy and strong Zn performance but does not surpass BiteNet_I on Zn^{2+} in either benchmark (see updated Zn-specific tables and Supplementary Tables S5–S6). AllMetal3D achieves competitive performance for several ion types but remains below BiteNet_I on average across ions. We also report runtime measurements for AllMetal3D in Supplementary Figure S1, which show that AllMetal3D is substantially slower (tens of seconds per protein on our hardware) than BiteNet_I in its default 50-orientation mode (~ 16 s per protein), and much slower than sequence-based baselines.

2.1.2) LMetalSite: This method is mentioned in the manuscript but it is absent from the main comparison plots (Figure 2). The data in Supplementary Table S5, where it is included, suggests that BiteNetI might not offer a consistent, significant improvement over LMetalSite.

We agree with the reviewer that LMetalSite deserves a more detailed comparison. In the revised manuscript we have made two main changes. First, we updated the main comparison figure (Figure 2) so that LMetalSite is now shown alongside IonCom, MIonSite, M-Ionic, PT-LM-GNN and BiteNet_I on both benchmarks. Second, we reran the authors' LMetalSite implementation on the MIonSite and IonCom sequences and evaluated its per-ion performance in the same framework as BiteNet_I. LMetalSite outputs, for each supported ion (Ca^{2+}, Mg^{2+}, Zn^{2+}, Mn^{2+}), both residue-wise probabilities (`{ION}_prob`) and binary predictions (`{ION}_pred`) in CSV format. In our evaluation we use the probabilities for AUC and average precision and the provided binary labels for accuracy, recall, specificity, F1-score and MCC, without applying any additional thresholding on our side. These details are described in the new Supplementary Methods subsection and in the updated Methods baseline block. We discuss these results in Section 2.2 of the revised manuscript. On the MIonSite benchmark, BiteNet_I matches or exceeds LMetalSite for all four ions supported by LMetalSite and achieves notably higher MCC for Zn^{2+} and Mn^{2+} (Supplementary Table S5).

On the IonCom benchmark, BiteNet$_I$ also performs better on average across these ions, although the improvement over LMetalSite for Zn^{2+} is less prominent (Supplementary Table S6). We additionally report runtime measurements for LMetalSite, which show that it is substantially faster (on the order of 0.1–0.2 s per protein) than structure-based methods, including BiteNet$_I$.

2.1.3) GPred [4]: including a Graph Neural Network in the comparisons could increase the value of the paper by exposing the advantages and limitations of different architectures. If performance numbers for key methods are not available in the literature, those methods should be run to provide a direct and fair comparison.

We agree with the reviewer, that including comparison with GNN-based method is interesting. We have addressed this Remark as follows. We have attempted to include GPred suggested by the Reviewer but encountered technical limitations that prevented us from obtaining reliable predictions on IonCom and MIonSite. More specifically, we examined the public GitHub repository `https://github.com/wn1225/GPred` referenced in the GPred paper [4] and the web link `https://www.musite.net/`. The repository does not contain a working inference script for test sequences. The script `GPred_5fold_train.py`, which is suggested as the main entry point, depends on helper classes and variables (e.g. `EarlyStopping`, `weight`) that are not defined anywhere in the repository, so we could not run even the training code. The web link `https://www.musite.net/` points to a different method and does not provide a way to obtain GPred predictions for arbitrary sequences. Therefore, we discarded this method from consideration. We also tried to run several additional predictors:

- **MoM** [11] — requires the construction of a method-specific reference dataset, but the necessary preprocessing steps and command-line arguments are not documented in sufficient detail for us to reproduce the authors' pipeline on IonCom or MIonSite.
- **PINmyMETAL** [12] — the software can be run, but the output format does not clearly specify residue-level binding probabilities, and we were unable to derive unambiguous residue-wise scores compatible with the MIonSite and IonCom benchmarks.
- **IonPred** [13] — the public code does not provide trained model weights or information on where to obtain them, so we could not generate predictions.
- **DeepProSite** [14] — requires extensive environment setup and multiple non-trivial data preparation steps that go beyond what we could reasonably reconstruct from the available documentation.
- **PT-LM-GNN** [7] — does provides pre-trained models and inference code.

Therefore, we added a modern GNN-based baseline, PT-LM-GNN [7], to our comparisons. PT-LM-GNN combines protein language-model embeddings with a graph neural network on residue contact graphs to predict ligand-binding residues. We used the authors' pretrained models and inference code to compute residue-wise probabilities and binary predictions for Ca^{2+}, Mg^{2+}, Zn^{2+}, Mn^{2+} and Fe on the IonCom and MIonSite validation sets, and we evaluated these outputs alongside BiteNet$_I$ using our unified protocol (Supplementary Methods, Section External baselines and score computation, and Supplementary Tables S5–S6). The revised Section 2.2 states that BiteNet$_I$ achieves higher MCC than PT-LM-GNN for most ions on both benchmarks, indicating that our approach is advantageous to this GNN built on top of large pretrained sequence models.

Changes in the manuscript:

- Added to the main text, Benchmarking against existing methods:
  On the MIonSite benchmark, BiteNet$_I$ matched or exceeded recent sequence-based method LMetalSite for all four metal types: Ca^{2+}, Mg^{2+}, Zn^{2+} and Mn^{2+} (MCC: Ca^{2+} 0.641 vs 0.506; Mg^{2+} 0.415 vs 0.410; Zn^{2+} 0.843 vs 0.836; Mn^{2+} 0.805 vs 0.740) (see also Table S5) ; on the IonCom benchmark, BiteNet$_I$ also shows better average results across these ions compared to LMetalSite, M-Ionic and the graph-based PT-LM-GNN methods (mean MCC over Ca/Mg/Zn/Mn: 0.669 vs 0.661 (LMetalSite), 0.605 (M-Ionic), and 0.459 (PT-LM-GNN)) (see Table S6). We likewise evaluated AllMetal3D on both benchmarks; while it achieved competitive performance for several ion types, BiteNet$_I$ delivers larger average MCC (e.g., 0.675 vs 0.461 averaged over the IonCom ions reported for AllMetal3D; Table S6).
  In addition, we compared BiteNet$_I$ to Metal3D [15], a convolutional model trained specifically for Zn^{2+} binding site localization. We applied Metal3D to the IonCom and MIonSite Zn^{2+} validation sets and converted the predicted Zn^{2+} positions into residue-wise scores. While Metal3D showed high accuracy on both benchmarks, BiteNet$_I$ demonstrated better performance (Tables S6 and S5). To compare the models on the original Metal3D binding site test set, we ran BiteNet$_I$ on the same biological assemblies and used the evaluation protocol of Dürr et al. [15], i.e. we filtered Zn^{2+} sites with fewer than two unique protein ligands within 2.8 Å and occupancy ≤ 0.5 and treated predictions within 5 Å of an experimental Zn^{2+} site as true positives. On the resulting set of 132 Zn^{2+} sites in 54 assemblies, Metal3D with a probability cutoff of $p = 0.5$ reached a

precision of 0.76, recall of 0.68 and F1-score of 0.72, whereas BiteNet$_I$ with the default score threshold of 0.5 achieved precision 0.81, recall 0.78 and F1-score 0.80 with a comparable number of false-positive clusters. Because the Metal3D test set partially overlaps with our training data, we additionally removed 34 assemblies with sequence identity ≥ 0.3 or TM-score ≥ 0.5 to any training structure, which resulted in a non-redundant subset of 51 Zn^{2+} sites in 23 assemblies. On this subset Metal3D ($p = 0.5$) obtained precision 0.71, recall 0.69 and F1-score 0.70, while BiteNet$_I$ (threshold 0.5) reached precision 0.78, recall 0.92 and F1-score 0.85. Detailed metrics for both datasets and additional score thresholds are provided in Supplementary Tables S7 and S8. These results suggest that multi-ion predictive models may outperform single-ion ones.
With the advances in protein complex structure prediction, it is also interesting to compare BiteNet$_I$ with AlphaFold3 [2] by Google DeepMind (`https://deepmind.google/`), a successor of AlphaFold2 [3] with enhanced capability to predict structures of molecular complexes, including protein-ion complexes. For each protein in the MIonSite and IonCom benchmarks we provided to AlphaFold3 the amino acid sequence together with a single copy of the relevant ion as input, ran the AlphaFold3 pipeline to generate five structural models, and derived residue-level ion-binding scores from these models to compute the same performance metrics as for other methods (see Section 3 for details). BiteNet$_I$ shows clear improvements over AlphaFold3 for several monoatomic ions (e.g. Ca^{2+}, K^+, Mg^{2+}) and polyatomic ions (e.g. PO_4^{3-}, SO_4^{2-}), while AlphaFold3 performs slightly better for CO_3^{2-} and Na^+. We want to emphasize, however, that comparison with AlphaFold3 should not be interpreted as a fair head-to-head benchmark. The two methods aim to solve very different computational tasks: BiteNet$_I$ assumes that a protein structure is already available and predicts ion-binding sites on top of it, whereas AlphaFold3 predicts the full three-dimensional structure of the protein–ion complex directly from sequence (and ligand) information and therefore does not require an input structure. Moreover, MIonSite and IonCom benchmarks likely overlap with the training data of AlphaFold3, so its performance should be viewed as an upper bound for what can be obtained from a state-of-the-art end-to-end structure predictor.
We further explored how BiteNet$_I$ performed on the AlphaFold2-generated models, rather than experimentally determined structures. Specifically, we used models from the AlphaFold Protein Structure Database [4] as input to BiteNet$_I$. This approach also demonstrated competitive performance, but in general did not outperform BiteNet$_I$ applied to experimental structures. // As for the computational speed, in our experiments a single forward pass of the multi-task BiteNet$_I$ model takes $\sim$ 0.31,s per orientation (see Figure S1) and 16.0 ± 0.5 s per 50 random orientations of a structure. This makes large-scale annotation of ion-binding sites feasible when experimental or predicted structures are available. To compare with the other methods, we evaluated runtimes elapsed on the MIonSite and IonCom benchmarks (Figure S1). Sequence-based models were, as expected, the fastest: M-Ionic [5] and LMetalSite [6] required on average 0.08 ± 0.08 s and 0.17 ± 0.41 s per protein, respectively. Among structure-based methods, the graph-based PT-LM-GNN [7] and the voxel-based AllMetal3D [8] were substantially more demanding, with mean runtimes of 15.9 ± 20.0 s and 45.8 ± 189.0 s per protein. To provide contextual scale, we also recorded the elapsed runtime of AlphaFold3 (Figure S1), which performs a much more complex sequence-to-structure prediction task.
Finally, we would like to note that the training datasets employed by different studies are, strictly speaking, different due to different preprocessing pipelines, temporal or similarity cutoffs, and other reasons. Furthermore, several recent approaches (e.g. LMetalSite, M-Ionic, PT-LM-GNN) leverage pretrained protein language models, which were exposed to a large corpus of protein sequences. Consequently, the MIonSite and IonCom validation sets used here may overlap with the training and/or pre-training data of some methods. Therefore, the reported numbers should not be interpreted as strictly non-redundant test-set evaluation of all the methods.

- Updated in the main text: Figure 2 (added methods).
- Updated in SI: Tables S5 S6 (added scores for Metal3D, AllMetal3D, LMetalSite, M-Ionic, PT-LM-GNN), Figure S3 (added methods)
- Added to the main text, Reference methods used for comparison:
The metrics for the reference methods reported in Figures 2,S3 and Tables S5,S6 are based on the original publications listed below or by running the authors' released implementations as described here. For earlier methods (MetalDetector v2.0, S-SITE, COACH, TargetS, IonSeq, IonCom, MIB, MIonSite and GASS-Metal) we directly used the reported test-set performance and did not re-train or re-evaluate the models. For more recent baselines (LMetalSite, M-Ionic, PT-LM-GNN, Metal3D, AllMetal3D and AlphaFold3) we used the authors' released implementations and pre-trained models to obtain predictions on the MIonSite and IonCom validation sets and computed the metrics in a unified way as described below.
and further for LMetalSite:
We ran the authors' implementation with default settings and used the per-residue probabilities output for these four ions as scores for the MIonSite and IonCom sequences.

and further for M-Ionic:
We used the released model and inference script to obtain residue-wise probabilities on the MIonSite and IonCom validation sets and interpreted the ion-specific outputs as binding scores for each residue.
and further for AlphaFold3:
Note, that because the precise training data and temporal cut-off used to train AlphaFold3 have not been released, we did not attempt to construct a time-split benchmark with respect to its training set. Therefore, some MIonSite and IonCom complexes are likely to overlap with structures seen during AlphaFold3 training, and the reported AlphaFold3 scores should be interpreted with caution.
and further for Metal3D:
Metal3D (2023) [15] Metal3D [15] is a convolutional neural network that predicts three-dimensional probability maps for Zn^{2+} positions on a voxel grid around a protein structure. We used the authors' implementation and followed their evaluation protocol for the Zn^{2+} binding site test set. Briefly, Zn^{2+} sites with fewer than two unique protein ligands within 2.8 Å and occupancy ≤ 0.5 were removed from the ground truth, and a predicted Zn^{2+} site was counted as a true positive if it was within 5 Å of an experimental site. False-positive predictions were clustered with a 5 Å radius and counted once per cluster. For comparison with residue-level benchmarks (IonCom and MIonSite), we converted Metal3D Zn^{2+} site predictions to residue-wise scores using the following approach. The residue score is zero if there is no predicted ion close to it, and residue score is equal to the Metal3D center score of the predicted ion if distance from any heavy atom of residue to that ion is $\leq 0.5 + vdw_1 + vdw_2$Åfor MIonSite and ≤ 5Å for IonCom benchmarks.
and further for PT-LM-GNN:
PT-LM-GNN (2025) [7] combines pretrained protein language-model embeddings with a graph neural network operating on residue graphs to predict ligand-binding residues. We used the authors' pretrained weights and inference code to compute ion-specific residue probabilities for Ca^{2+}, Mg^{2+}, Zn^{2+}, Mn^{2+} and Fe ions on the MIonSite and IonCom validation sets. For a small number of structures the public implementation failed; in these cases we assigned zero residue probabilities for metric aggregation.
and further for AllMetal3D:
AllMetal3D (2025) [8] extends Metal3D to joint prediction of ion identity, localization and coordination geometry for several common metal ions on a 3D voxel grid. We ran the authors' pipeline on the MIonSite and IonCom structures, selected ion-specific probe centres from the predicted probability maps and converted these to residue-wise scores using the same distance-based mapping as for Metal3D. As for PT-LM-GNN, some proteins could not be processed successfully by the public implementation; these cases were assigned zero residue probabilities when computing aggregate benchmark statistics.

- Added section to SI: External baselines and score computation

2.2) Metric reporting: The authors focus on MCC and mwAP in the main text. While we agree with the point about the importance of those metrics (last paragraph of Section 3.4), the supplementary material suggests that BiteNetI might be performing slightly worse on other metrics (for example in terms of accuracy in Table S5). This selective reporting, combined with the missing baselines, gives an unfavorable impression and makes it difficult to truly assess if the proposed method achieves state-of-the-art performance. For a transparent and fair comparison, the main text must contain at least a concise overview of results on all standard metrics.

In this work we emphasize mwAP and MCC because the residue-level binding site detection task is highly imbalanced, and several commonly reported metrics (accuracy, sensitivity/recall, specificity) are threshold-dependent and can be misleading; accuracy and ROC AUC may also appear overly optimistic under severe imbalance. To make this explicit, we expanded the discussion of metric suitability and limitations in the main manuscript (Section 3.4, Metrics). For completeness, we also report the standard metrics used in prior studies (MCC, accuracy, sensitivity, specificity, and ROC AUC) alongside our primary metrics in the tables, and we refer to the Supplementary Tables S5, S6 for the full breakdown. AP/ROC AUC can only be computed when per-residue prediction scores are available; therefore, for some baselines we report only the threshold-based metrics as provided in the original publications.

Changes in the manuscript:

- Updated in the main text, Metrics (last paragraph):
We would like to stress that several common performance metrics (including MCC, accuracy, and sensitivity (recall), and specificity) are threshold-dependent. Due to the strong class imbalance (low ratio of binding residues), sensitivity and specificity can be trivially driven to extreme values by predicting almost all residues as binding or non-binding, and accuracy may appear overly optimistic because it is dominated by the majority (non-binding) class. MCC is more appropriate for imbalanced data, but it still varies with the chosen operating threshold and therefore must be interpreted together with the thresholding protocol. ROC AUC is threshold-independent, yet it can also look optimistic under severe imbalance and does not directly reflect

precision at practically relevant recall levels. In contrast, the AP metric evaluates the ranking of predictions and is therefore well-suited for highly imbalanced detection tasks; we thus emphasize AP alongside MCC. Nevertheless, we report MCC, accuracy, sensitivity, specificity, and ROC AUC in the main tables. AP/ROC AUC can only be computed when per-residue prediction scores are available; hence for some baselines we report only threshold-based metrics as provided in the original studies.

2.3) AlphaFold3 comparison: While the comparison to AlphaFold3 (AF3) is interesting, AF3 was not designed or optimized for this specific task. Beating AF3 thus does not compensate for the lack of proper benchmarking of other tools in this area. The manuscript would be much stronger if it focused on comparing its performance against tools designed specifically for ion binding site prediction.

We agree with the reviewer. Our intention to use AlphaFold3 is to make a complementary comparison with a widely used modern structure prediction pipeline, and to show that despite the advances in the co-folding methods, the protein-ion binding site prediction problem is still not-resolved. We clarified this intention and softened the claims about comparison with AlphaFold3 in terms of accuracy and speed through the revised version of manuscript (see also response to Reviewer 2).

=== Positive Aspects ===

3) Despite these major flaws, the manuscript contains valuable work. In particular, the analyses focusing on the impact of PDB structure resolution and the inclusion of solvent effects are welcome additions. Also, the authors' effort to make the model accessible through an online application is appreciated, as it indicates significant work and commitment to tool usability.

=== Recommendations ===

4) The recommendation is that the authors improve the manuscript in the following four aspects:

4.1) Provide rigorous evidence (including ablation studies) for all claims of innovation, especially the data augmentation.

4.2) Conduct a thorough benchmark against all relevant SOTA methods, including Metal3D. LMetalSite results should be fairly reported in the main text.

4.3) Provide a concise overview of results for all standard metrics (AUROC, MCC, mwAP, Accuracy) and all methods in the main paper to allow for a fair and transparent comparison. Older methods could be omitted, but to assure fairness, similar structural methods (LMetalSite, 3DMetal [2]) need to be included, even if it requires running an inference.

4.4) Refocus the manuscript to ensure clarity and rigor—each claimed contribution should be clearly stated and well supported by evidence. Introduce innovations one at a time, demonstrating their individual impact through systematic experiments or analyses. Finally, show that the combination of these validated components leads to a state-of-the-art model.

We thank the reviewer for careful reading, valuable remarks, and positive feedback to our work.

References

[1] Yao Cheng, Haobo Wang, Hua Xu, Yuan Liu, Bin Ma, Xuemin Chen, Xin Zeng, Xianghe Wang, Bo Wang, Carina Shiau, Sergey Ovchinnikov, Xiao-Dong Su, and Chu Wang. Co-evolution-based prediction of metal-binding sites in proteomes by machine learning. *Nature Chemical Biology*, 19(5):548–555, May 2023.

[2] Josh Abramson, Jonas Adler, Jack Dunger, Richard Evans, Tim Green, Alexander Pritzel, Olaf Ronneberger, Lindsay Willmore, Andrew J. Ballard, Joshua Bambrick, Sebastian W. Bodenstein, David A. Evans, Chia-Chun Hung, Michael O'Neill, David Reiman, Kathryn Tunyasuvunakool, Zachary Wu, Akvilė Žemgulytė, Eirini Arvaniti, Charles Beattie, Ottavia Bertolli, Alex Bridgland, Alexey Cherepanov, Miles Congreve, Alexander I. Cowen-Rivers, Andrew Cowie, Michael Figurnov, Fabian B. Fuchs, Hannah Gladman, Rishub Jain, Yousuf A. Khan, Caroline M. R. Low, Kuba Perlin, Anna Potapenko, Pascal Savy, Sukhdeep Singh, Adrian Stecula, Ashok Thillaisundaram, Catherine Tong, Sergei Yakneen, Ellen D. Zhong, Michal Zielinski, Augustin Žídek, Victor Bapst, Pushmeet Kohli, Max Jaderberg, Demis Hassabis, and John M. Jumper. Accurate structure prediction of biomolecular interactions with AlphaFold 3. *Nature*, 630(8016):493–500, June 2024.

[3] John Jumper, Richard Evans, Alexander Pritzel, Tim Green, Michael Figurnov, Olaf Ronneberger, Kathryn Tunyasuvunakool, Russ Bates, Augustin Žídek, Anna Potapenko, et al. Highly accurate protein structure prediction with alphafold. *Nature*, 596(7873):583–589, 2021.

[4] Mihaly Varadi, Stephen Anyango, Mandar Deshpande, Sreenath Nair, Cindy Natassia, Galabina Yordanova, David Yuan, Oana Stroe, Gemma Wood, Agata Laydon, Augustin Žídek, Tim Green, Kathryn Tunyasuvunakool, Stig Petersen, John Jumper, Ellen Clancy, Richard Green, Ankur Vora, Mira Lutfi, Michael Figurnov, Andrew Cowie, Nicole Hobbs, Pushmeet Kohli, Gerard Kleywegt, Ewan Birney, Demis Hassabis, and Sameer Velankar. AlphaFold Protein Structure Database: Massively expanding the structural coverage of protein-sequence space with high-accuracy models. *Nucleic Acids Research*, 50(D1):D439–D444, January 2022.

[5] Aditi Shenoy, Yogesh Kalakoti, Durai Sundar, and Arne Elofsson. M-Ionic: Prediction of metal-ion-binding sites from sequence using residue embeddings. *Bioinformatics*, 40(1):btad782, January 2024.

[6] Qianmu Yuan, Sheng Chen, Yu Wang, Huiying Zhao, and Yuedong Yang. Alignment-free metal ion-binding site prediction from protein sequence through pretrained language model and multi-task learning. *Briefings in Bioinformatics*, 23(6):bbac444, November 2022.

[7] Hamza Gamouh, Marian Novotný, and David Hoksza. Hybrid protein–ligand binding residue prediction with protein language models: Does the structure matter? *Bioinformatics*, 41(8):btaf431, August 2025.

[8] Simon L. Dürr and Ursula Rothlisberger. AllMetal3D: Joint prediction of localization, identity and coordination geometry of common metal ions in proteins, February 2025.

[9] Michael Piazza, Valentina Taiakina, Thorsten Dieckmann, and J. Guy Guillemette. Structural Consequences of Calmodulin EF Hand Mutations. *Biochemistry*, 56(7):944–956, February 2017.

[10] Benjamin Webb and Andrej Sali. Comparative Protein Structure Modeling Using MODELLER. *Current Protocols in Bioinformatics*, 54(1):5.6.1–5.6.37, 2016.

[11] Vincenzo Laveglia, Milana Bazayeva, Claudia Andreini, and Antonio Rosato. Hunting down zinc(II)-binding sites in proteins with distance matrices. *Bioinformatics*, 39(11):btad653, November 2023.

[12] Heping Zheng, Huihui Zhang, Juanhong Zhong, Michal Gucwa, Yishuai Zhang, Haojie Ma, Lei Deng, Longfei Mao, Wladek Minor, and Nasui Wang. PinMyMetal: A hybrid learning system to accurately model metal binding sites in macromolecules. *Research Square*, pages rs.3.rs–3908734, February 2024.

[13] Clement Essien, Lei Jiang, Duolin Wang, and Dong Xu. Prediction of Protein Ion–Ligand Binding Sites with ELECTRA. *Molecules*, 28(19):6793, January 2023.

[14] Yitian Fang, Yi Jiang, Leyi Wei, Qin Ma, Zhixiang Ren, Qianmu Yuan, and Dong-Qing Wei. DeepProSite: Structure-aware protein binding site prediction using ESMFold and pretrained language model. *Bioinformatics*, 39(12):btad718, December 2023.

[15] Simon L. Dürr, Andrea Levy, and Ursula Rothlisberger. Metal3D: A general deep learning framework for accurate metal ion location prediction in proteins. *Nature Communications*, 14(1):2713, May 2023.

Multivalent ion binding site identification with structure-based deep learning
Response to reviewers

April 17, 2026

We sincerely thank the editor and the reviewers for their valuable feedback and positive evaluation of our work. We have addressed the remaining minor issues and provide the corresponding remarks below.

Reviewer 1

I am happy with the authors' responses and revision. From my point of view, it should be OK to be published now.

We thank the reviewer for the positive evaluation of our revision and for supporting publication of the manuscript.

Reviewer 2

I thank the authors for their thorough and constructive response to the first-round comments.

The revised manuscript is substantially improved, and the new additions considerably strengthen the work. The authors have addressed all major concerns. I am satisfied with the revision and support publication, pending resolution of the following minor remaining issues.

Residual inconsistency in GPCR case study language (Section 2.3)

The authors clarified the water representation in the general paragraph of Section 2.3. However, the GPCR case study paragraph retains the original problematic phrasing: "Upon including crystallographic water molecules as hydroxyl oxygen atoms in the input grid..." This sentence was not updated and directly conflicts with the clarification made just paragraphs earlier. Please revise this sentence to use language consistent with the rest of the corrected Section 2.3 (e.g., "upon including crystallographic water molecules in the input voxel grid via the polar-oxygen channel...").

We have updated the misleading sentence in the revised version of the manuscript.

Dataset size numbers potentially confusing for readers

The Introduction states "a manually curated dataset of $\sim$10,000 protein–ion complex structures", while the Abstract states "over 75,000 high-resolution protein–ion complexes." The distinction between the number of PDB structures ($\sim$10,000) and the number of individual ion-binding site instances ($\sim$75,000 after augmentation) is important but may confuse a first-time reader. A brief parenthetical clarification at the first occurrence of each number would improve readability.

We thank the reviewer for pointing this out. We revised the Abstract to refer consistently to the manually curated dataset of over 10,000 high-resolution protein–ion complex structures, in line with the Introduction; and the number of the binding sites is in Methods (Dataset construction).

Summary

The authors have responded comprehensively, and the core methodology, benchmarking, and generalization evidence are now of publishable quality. The points above are all minor and can be resolved in a brief final revision without re-review. I recommend acceptance contingent on these corrections.

We thank the reviewer for the positive assessment of the revised manuscript and for these helpful final suggestions. We have addressed both minor points in the revised version.

Reviewer 3

Review Summary

The authors have thoroughly and thoughtfully addressed the reviewers' feedback. The revisions substantially improve the clarity, rigor, and completeness of the manuscript. One concern remains regarding the limited empirical support for the benefits of the proposed data augmentation, but we believe this can be addressed with a minor additional revision.

We thank the reviewer for the positive assessment and for recognizing the improvements in the revised manuscript. To address the remaining concern, we performed an additional analysis and added the resulting quantitative evidence to the Supplementary Information.

Concerns

While the authors attempt to address the previous concern about the lack of evidence for the benefits of the proposed dataset by reframing it as a "label consistency" method, the paper still lacks numerical evidence that label inconsistency was a real problem in the first place. The reasoning is plausible, but the manuscript does not show even small side-by-side improvements in model generalization. Such improvements do not need to be large or consistent across all protein targets or ion types; however, the proposed innovation should be supported by quantitative evidence rather than assumptions about potential issues in existing datasets. Since the authors describe label consistency as a form of regularization, it should be possible to demonstrate improved generalization in at least some experimental settings. We therefore recommend performing additional experiments or designing new evaluation setups if necessary, to demonstrate the benefits of the proposed data augmentation and "label consistency" approach, as showing its effectiveness could significantly strengthen the paper's impact.

To address this point, we performed an analysis on groups of corresponding ion-binding sites derived from the superposition-based transfer procedure. Namely, we compared the score distributions from models trained without augmentation and with augmentation on near-identical binding sites from the test set and observed a modest but favorable trend overall: the augmented model showed lower score dispersion in 19 of 31 group–label combinations (61.3%) and higher mean score in 20 of 31 combinations (64.5%). We have added the results to the Supplementary Information (see Supplementary Figure S1).

Positive Aspects

The authors effectively addressed other previous major concerns regarding the claims of methodological innovation and inadequate benchmarking. Most notable improvements:

- **Reframed Methodological Claims:** The authors appropriately adjusted their claims regarding data augmentation. By reframing the ion-transfer augmentation as a regularization and label-consistency mechanism rather than the primary methodological novelty, the manuscript is now more scientifically accurate.
- **Comprehensive Benchmarking:** We highly appreciate the massive effort put into the expanded benchmarking. Including recent state-of-the-art baselines like LMetalSite, M-Ionic, PT-LM-GNN, Metal3D, and AllMetal3D provides a much fairer and rigorous evaluation of BiteNet1's capabilities.
- **Specialist vs. Generalist Evaluation:** Evaluating BiteNet1 directly against the specialized Metal3D model on its own Zn^{2+} test set was a great addition that strongly validates the advantages of the multi-ion approach.

We thank the reviewer for the positive assessment of these aspects of the revised manuscript.

Recommendations

Accept after revision.

We thank the reviewer for supporting acceptance after this final revision.
