# Supplementary material for: Multivalent ion binding site identification with structure-based deep learning
Source: Commun Biol. 2026 Jul 27;9:1039. doi: 10.1038/s42003-026-10659-1 (PMC13434133; doi:10.1038/s42003-026-10659-1)
Supplement: Supplementary file 2 [file 42003_2026_10659_MOESM2_ESM.pdf]

Supplementary Information for

Multivalent ion binding site identification with structure-based deep learning

Igor Kozlovskii[1,2] and Petr Popov[1,2]

[1]Constructor University Bremen gGmbH, Bremen 28759, Germany
[2]Constructor Labs, Bremen 28759, Germany

June 24, 2026

Supplementary Table 1: Performance scores on 5-fold cross-validation and on the test set. Values in the Mean row are mean ± s.d. over $n = 5$ cross-validation folds.

Set	mAP	-3	-2	-1	+1	+2	+3
Fold 0	0.57	0.29	0.27	0.25	0.38	0.73	0.68
Fold 1	0.55	0.40	0.27	0.14	0.33	0.61	0.88
Fold 2	0.54	0.36	0.25	0.21	0.27	0.79	0.88
Fold 3	0.54	0.25	0.26	0.14	0.33	0.74	0.87
Fold 4	0.52	0.27	0.29	0.10	0.37	0.72	0.64
Mean	0.54±0.01	0.31±0.05	0.27±0.01	0.17±0.05	0.34±0.04	0.72±0.06	0.79±0.11
Test	0.53	0.29	0.26	0.19	0.32	0.66	0.79

CA	CL	CO	CU	FE	FE2	K	MG
0.72	0.19	1.00	0.95	0.72	0.97	0.80	0.41
0.74	0.16	0.82	0.86	0.97	0.76	0.45	0.39
0.68	0.26	0.56	0.71	0.93	0.85	0.55	0.67
0.76	0.21	0.73	0.89	0.84	1.00	0.63	0.55
0.73	0.17	0.87	0.99	0.80	0.72	0.28	0.41
0.73±0.03	0.20±0.04	0.80±0.15	0.88±0.10	0.85±0.09	0.86±0.11	0.54±0.17	0.48±0.11
0.63	0.29	0.86	0.73	0.94	0.87	0.43	0.52

MN	NA	NI	PO4	SO4	ZN		
0.94	0.38	0.73	0.29	0.26	0.88		
0.96	0.33	0.74	0.39	0.27	0.89		
0.92	0.26	0.59	0.36	0.25	0.84		
0.86	0.31	0.68	0.24	0.25	0.83		
0.75	0.43	0.95	0.27	0.29	0.90		
0.88±0.08	0.34±0.06	0.74±0.12	0.31±0.05	0.27±0.01	0.87±0.02		
0.77	0.33	0.75	0.29	0.26	0.92		

Supplementary Table 2: Performance of $\text{BiteNet}_{\text{I}}$ model across specific ion types in the test set. The performance metrics include average precision (AP), area under the receiver-operating characteristic curve (ROC AUC), and Matthews correlation coefficient (MCC).

Class	AP	ROC AUC	MCC
-3	0.369	0.889	0.417
-2	0.316	0.888	0.353
-1	0.212	0.853	0.276
+1	0.364	0.837	0.393
+2	0.646	0.937	0.637
+3	0.799	0.979	0.778
CA	0.672	0.947	0.649
CL	0.331	0.874	0.367
CO	0.790	0.978	0.783
CU	0.655	0.983	0.588
FE	0.927	0.996	0.865
FE2	0.950	0.996	0.944
K	0.472	0.934	0.424
MG	0.446	0.907	0.500
MN	0.725	0.953	0.704
NA	0.363	0.816	0.402
NI	0.716	0.936	0.730
PO4	0.368	0.889	0.421
SO4	0.315	0.890	0.349
ZN	0.904	0.985	0.857

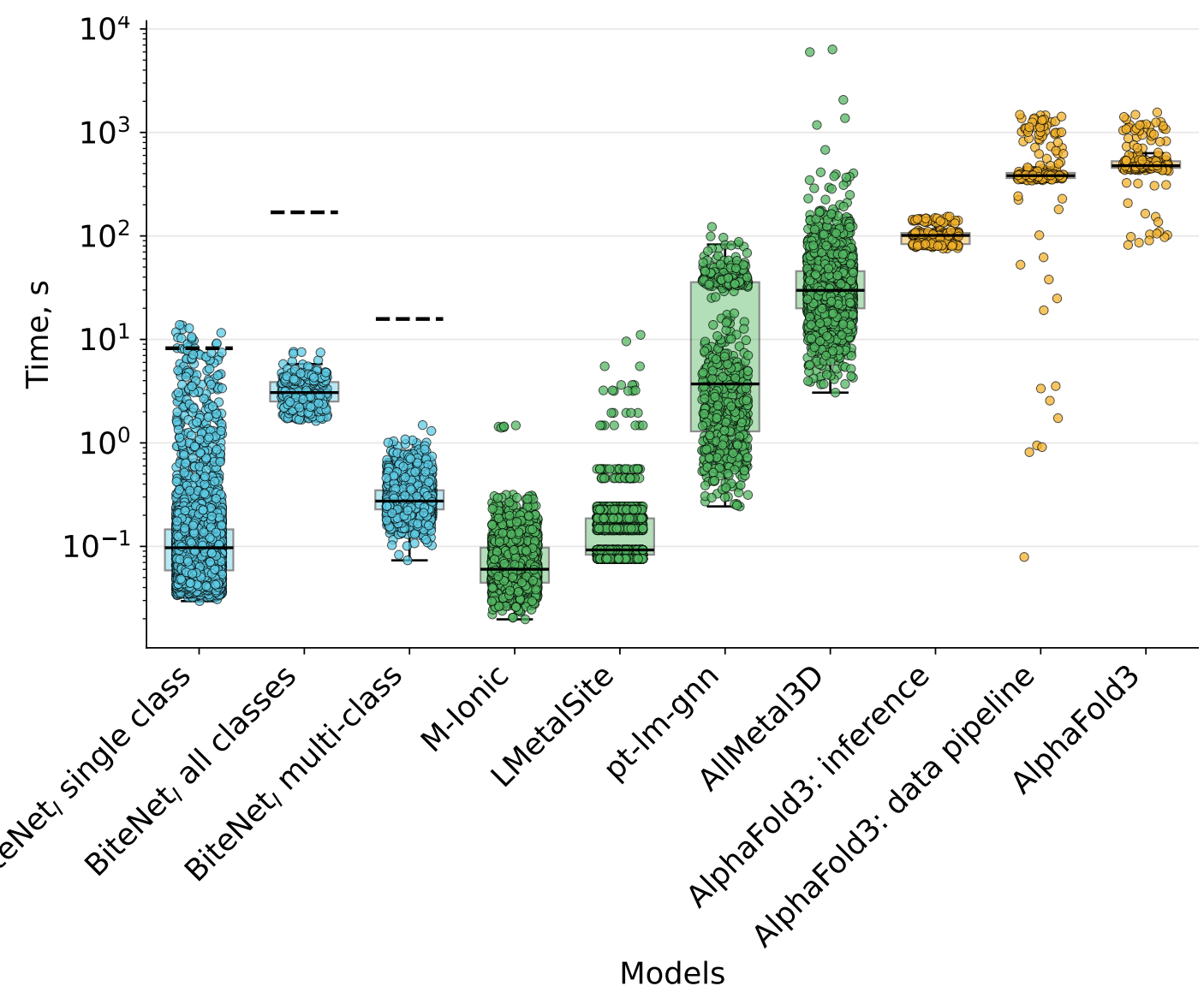

Supplementary Figure 1: **Runtime distributions for BiteNet$_\mathrm{I}$, reference ion-binding predictors, and AlphaFold3, shown on a logarithmic scale.** Boxplots show the median and interquartile range, with whiskers extending to 1.5 times the interquartile range. For BiteNet$_\mathrm{I}$, timings correspond to single-orientation predictions. "BiteNet$_\mathrm{I}$ single class" denotes ion-specific single-class models, "BiteNet$_\mathrm{I}$ all classes" denotes the summed runtime of applying all single-class models separately, and "BiteNet$_\mathrm{I}$ multi-class" denotes a single multitask model predicting all classes jointly. Dashed horizontal markers indicate the corresponding BiteNet$_\mathrm{I}$ runtimes after aggregation over 50 orientations. For external methods, each timing record corresponds to one benchmark prediction job. Sample sizes are: BiteNet$_\mathrm{I}$ single class, (n = 7078); BiteNet$_\mathrm{I}$ all classes, (n = 419); BiteNet$_\mathrm{I}$ multi-class, (n = 1992); M-Ionic, (n = 2374); LMetalSite, (n = 2374); PT-LM-GNN, (n = 748); AllMetal3D, (n = 2392); AlphaFold3 data pipeline, (n = 264); AlphaFold3 inference, (n = 234); AlphaFold3 total runtime, (n = 228). AlphaFold3 was run on a machine with Intel(R) Xeon(R) w7-3455 CPU and NVIDIA GeForce RTX 4090 GPU.

Supplementary Table 3: Statistics on the number of predictions located within 1Å distance from ions and non-ion small molecules in the structure. Number and fraction from all predictions are shown in respective columns.

Ion	All	Close to ion		Close to small mol	
		Num	Frac	Num	Frac
	81185	17559	0.216	5631	0.069
+1	4645	1097	0.236	71	0.015
+2	4129	1191	0.288	227	0.055
+3	2251	1034	0.459	203	0.090
-1	6165	498	0.081	547	0.089
-2	5875	621	0.106	487	0.083
-3	3596	447	0.124	414	0.115
CA	2385	1008	0.423	18	0.008
CL	5129	475	0.093	343	0.067
CO	3327	986	0.296	274	0.082
CU	3386	990	0.292	237	0.070
FE	2269	959	0.423	209	0.092
FE2	2426	1026	0.423	188	0.077
K	4826	827	0.171	30	0.006
MG	3208	1155	0.360	120	0.037
MN	2945	1107	0.376	206	0.070
NA	4052	1073	0.265	44	0.011
NI	3207	1012	0.316	249	0.078
PO4	3597	452	0.126	412	0.115
SO4	6035	636	0.105	479	0.079
ZN	3046	772	0.253	255	0.084

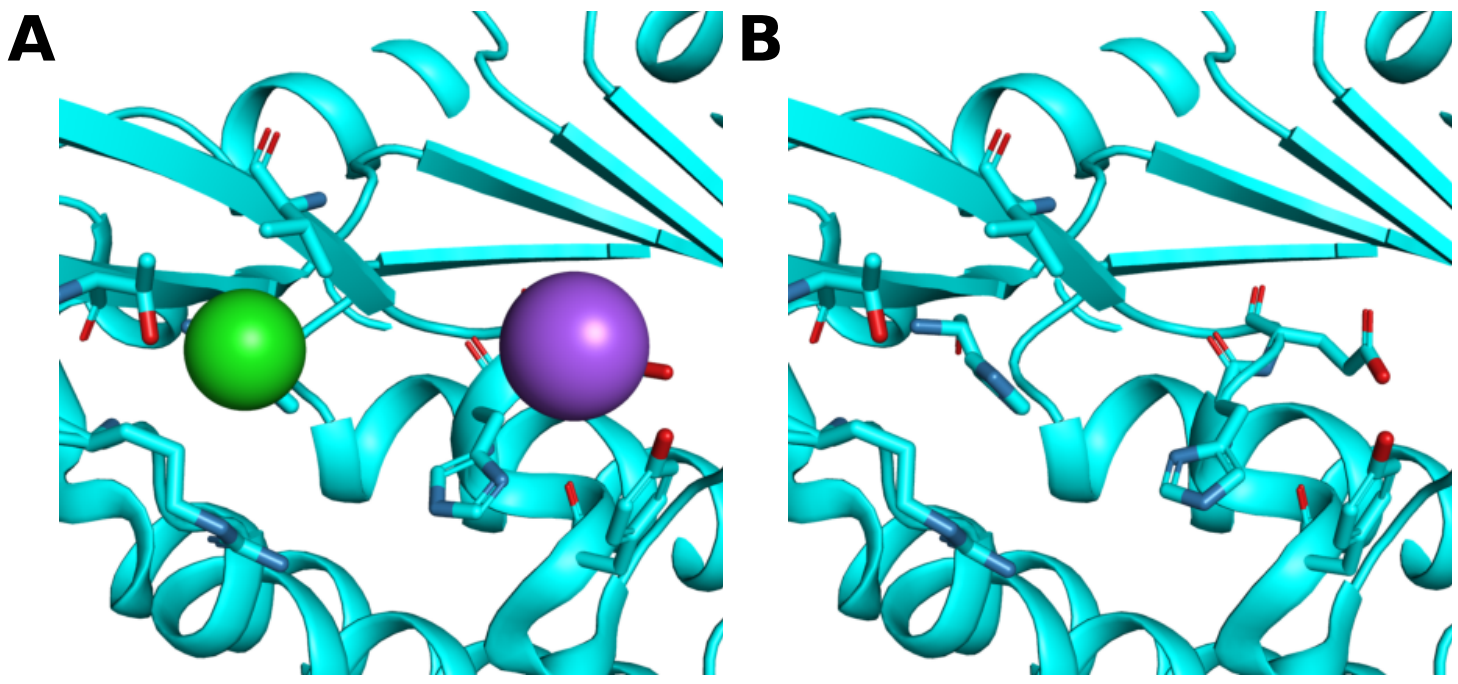

Supplementary Figure 2: **Ion presence differs between structurally similar aldehyde dehydrogenase structures.** Places occupied by Cl^- and Na^+ in one structure (A) (PDB ID: 4X4L) are empty in another structure (B) (PDB ID: 5AC2) of a Aldehyde Dehydrogenase 1A1 protein.

Supplementary Table 4: Effect of model configuration on five-fold cross-validation performance of the $\mathrm{BiteNet_I}$ model. Mean weighted average precision (mwAP $\pm$ s.d.) is reported over $n = 5$ cross-validation folds for each model configuration. mwAP is weighted by the inverse number of examples per class and reported for each combination of: Model head: multi-class output (Multi) vs. single-class heads (Single); Data augmentation: no added ions (Augmentation: -) vs. ions transferred from near-identical structures (Augmentation: +); Sampling strategy: cluster-based (Sampling +) vs. random. "mwAP (original)" denotes performance on the dataset without added ions; "mwAP (augmented)" on the dataset with transferred ions.

Multi-task	Augmentation	Sampling	mwAP on original structures (no added ions)	mwAP on augmented structures (with added ions)
multi	+	+	0.545 ± 0.016	0.544 ± 0.015
multi	+	-	0.537 ± 0.022	0.539 ± 0.018
multi	-	+	0.576 ± 0.023	0.571 ± 0.020
multi	-	-	0.572 ± 0.035	0.568 ± 0.032
single	+	+	0.541 ± 0.025	0.539 ± 0.023
single	+	-	0.540 ± 0.024	0.536 ± 0.023
single	-	+	0.550 ± 0.033	0.556 ± 0.034
single	-	-	0.548 ± 0.034	0.552 ± 0.036

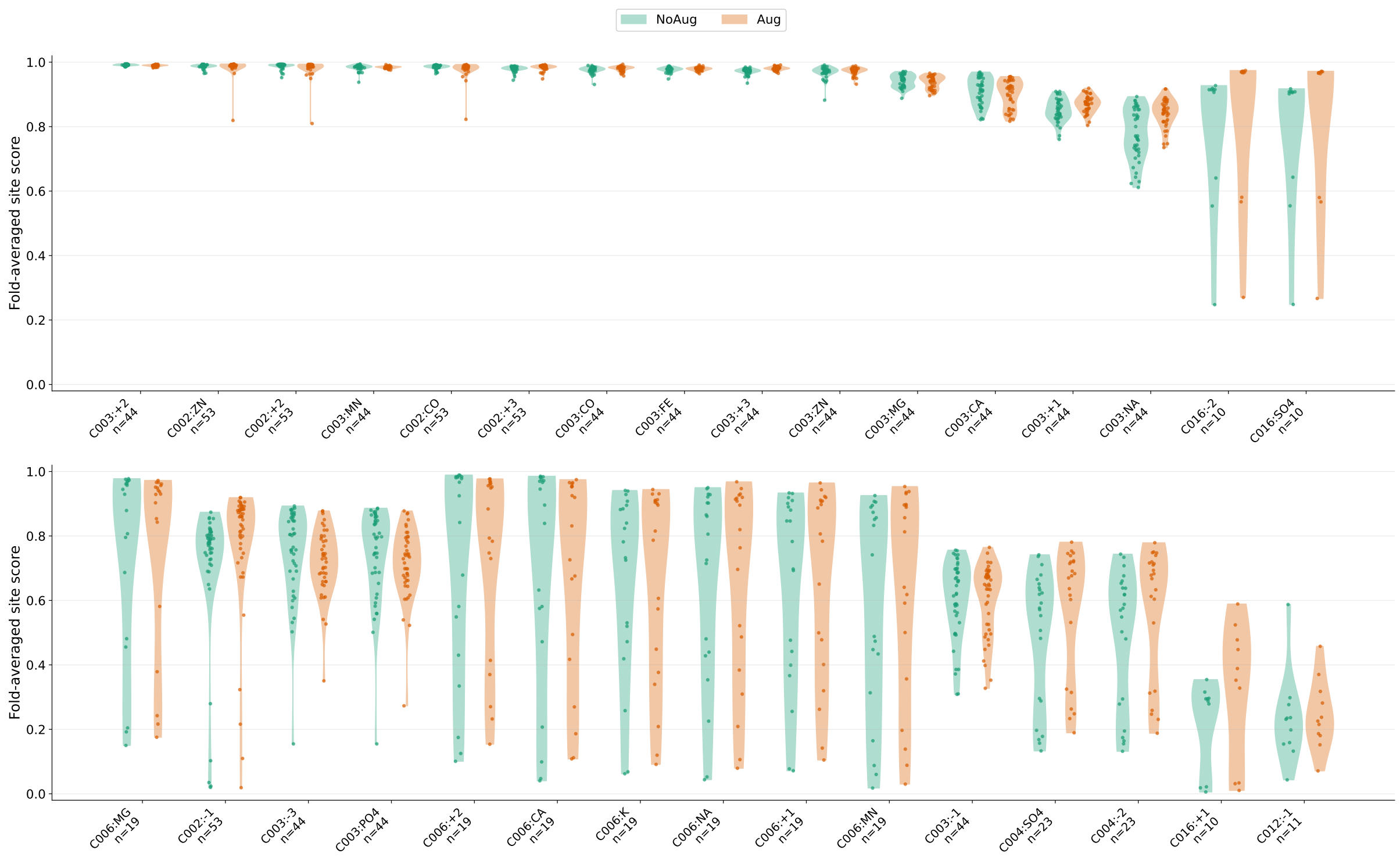

Supplementary Figure 3: **Ion-transfer augmentation modestly affects scores for ion-binding site prediction.** Score distributions for test-subset groups of corresponding ion-binding sites linked by the ion-transfer augmentation procedure. Group labels have the format "group id:class label". Only groups formed from structures in the test set with mean RMSD ≥ 0.3 Å and at least 10 binding-site samples are shown; exact group-specific sample sizes are indicated in the x-axis labels as ($n = X$) binding-site samples. For each group, the NoAug and Aug distributions are calculated from the same (n) binding-site samples. NoAug and Aug denote models trained without and with ion-transfer data augmentation, respectively. Each point denotes the fold-averaged predicted site score for one binding-site sample. Violin plots show the distribution of these fold-averaged site scores within each group. In total, ($n = 31$) groups are shown. The Aug model showed lower score standard deviation in 19 of 31 groups (61.3%) and higher mean score in 20 of 31 groups (64.5%).

Supplementary Table 5: Cluster-size statistics for the 14 specific ion types considered in this study. For each ion type we list the total number of binding sites in the dataset (#sites), the number of binding-site clusters (#clusters), the number of clusters of size 1 (#clusters size=1), and the sizes of the five largest clusters (Clusters 1–5). The strongly skewed, long-tailed distributions highlight substantial redundancy within many ion types and motivate the cluster-balanced sampling strategy used during training.

Ion	#sites	#clusters	#clusters size=1	Cluster 1	Cluster 2	Cluster 3	Cluster 4	Cluster 5
Ca^{2+} (CA)	5,830	771	251	933	420	271	261	216
Cl^{-} (CL)	11,192	1,399	427	574	430	337	260	260
Co^{2+} (CO)	1,237	66	9	200	152	113	64	59
Cu^{2+} (CU)	1,282	89	22	345	206	163	55	45
Fe^{3+} (FE)	1,108	114	18	187	94	59	47	44
Fe^{2+} (FE2)	574	49	6	74	62	53	35	28
K^{+} (K)	1,260	206	54	123	75	66	31	31
Mg^{2+} (MG)	3,937	652	196	368	204	107	89	87
Mn^{2+} (MN)	1,807	146	22	301	188	176	119	67
Na^{+} (NA)	6,953	811	254	1,043	277	226	192	177
Ni^{2+} (NI)	1,155	105	33	232	166	113	64	62
PO_4^{3-} (PO4)	3,944	596	191	253	250	146	136	115
SO_4^{2-} (SO4)	15,813	1,813	397	587	339	283	229	226
Zn^{2+} (ZN)	5,203	517	142	480	386	361	241	233

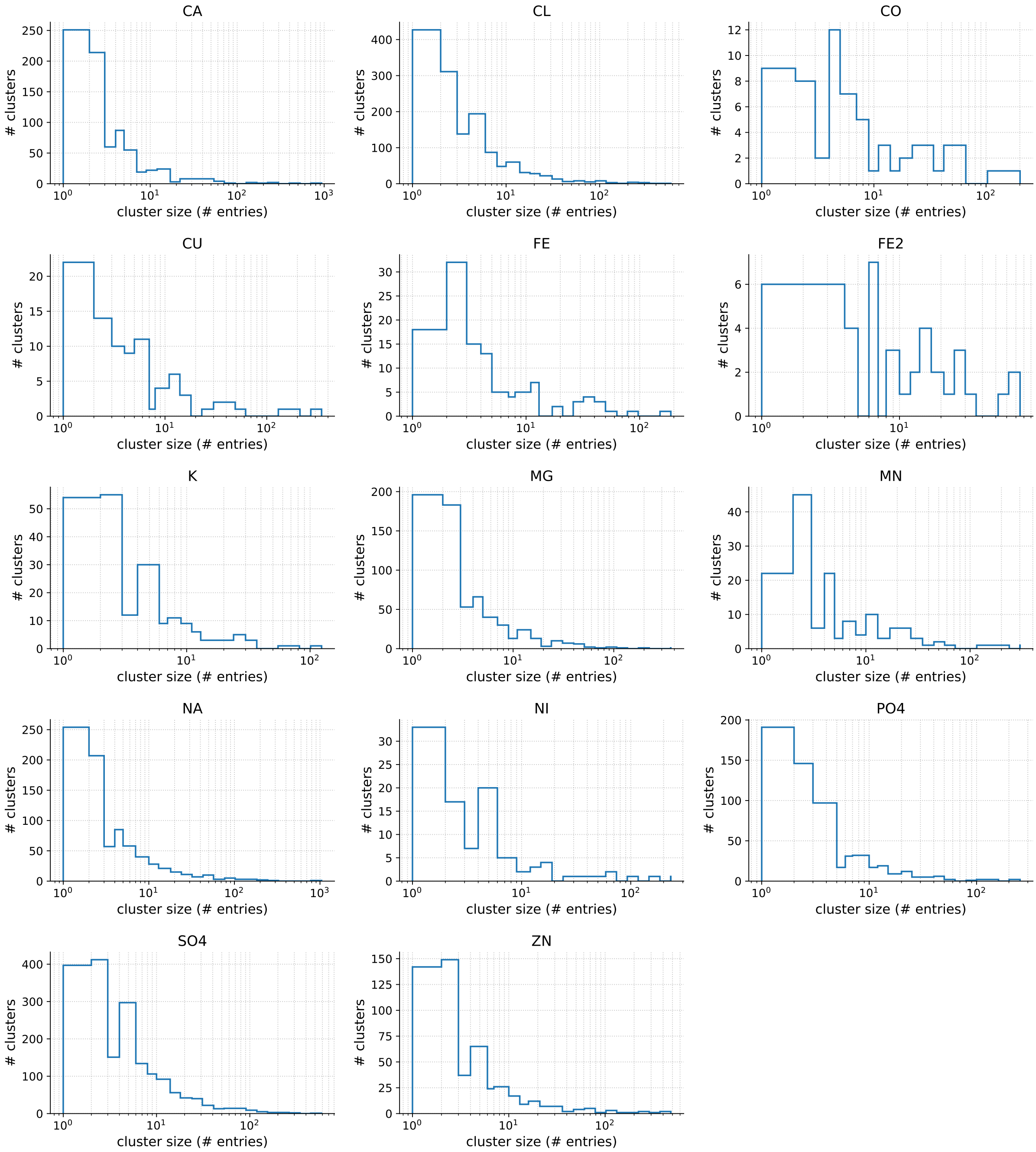

Supplementary Figure 4: **Distribution of cluster sizes for each ion class.** For a given ion, we group all binding site instances into clusters (as defined in the main text) and report the number of entries per cluster. Each panel shows a histogram of cluster sizes (number of entries per cluster) for one ion class; the x-axis is shown on a logarithmic scale.

Supplementary Table 6: Comparison of model performance on the MIonSite validation set. The reported metrics are taken from the original publications (MIonSite [1], M-Ionic [2], and references therein). Entries for Metal3D, AllMetal3D, PT-LM-GNN and marked as "calculated" were obtained by running the authors' released implementations/pretrained models and rescoring with our unified residue-level evaluation protocol (Section "Reference methods used for comparison" of the main text). BiteNet_I corresponds to the model trained in the MIonSite training set, and $\text{BiteNet}_\text{I}^*$ is for the model trained on the BiteNet_I training set.

Ion	Structures	Method	MCC	Accuracy	Sensitivity	Specificity	AP	ROC AUC
Zn^{2+}	69	MetalDetector [3]	0.56	0.98	0.38	1.00		
		S-SITE [4]	0.39	0.96	0.45	0.98		
		TargetS [5]	0.59	0.98	0.42	1.00		
		IonSeq [6]	0.35	0.92	0.70	0.93		
		MIB [7]	0.45	0.97	0.40	0.99		
		COACH [4]	0.42	0.98	0.32	0.99		
		IonCom [6]	0.47	0.95	0.77	0.96		
		MIonSite [1]	0.77	0.99	0.71	1.00		
		LMetalSite [8]	0.84	**0.99**	0.79	1.00		
		LMetalSite calculated	0.79	0.99	0.68	**1.00**	0.82	0.96
		GASS-Metal (1) [9]	0.52	0.99	0.52	0.99		
		GASS-Metal (2) [9]	0.65	0.99	0.65	0.99		
		GASS-Metal (3) [9]	0.76	0.99	0.76	1.00		
		M-Ionic [2]	0.76	0.99	0.77	0.99		
		M-Ionic calculated	0.74	0.99	0.69	1.00	0.56	0.84
		Metal3D [10]	0.74	0.99	**0.84**	0.99	0.83	0.94
		AllMetal3D [11]	0.72	0.99	0.57	**1.00**	0.61	0.81
		PT-LM-GNN [12]	0.57	0.98	0.66	0.98	0.64	0.88
		AlphaFold3 [13]	0.62	0.98	0.42	**1.00**	0.64	0.84
		AlphaFold2 [14]+BiteNet_I	0.71	0.99	0.63	1.00	0.71	0.90
		BiteNet_I	**0.84**	0.99	0.79	1.00	**0.88**	**0.98**
		$\text{BiteNet}_\text{I}^*$	0.82	0.99	0.76	1.00	0.86	0.97
Ca^{2+}	64	MetalDetector	0.02	0.98	0.01	1.00		
		S-SITE	0.16	0.98	0.10	0.99		
		TargetS	0.32	0.98	0.20	1.00		
		IonSeq	NA	0.98	0.00	**1.00**		
		MIB	0.21	0.98	0.18	0.99		
		COACH	0.12	0.96	0.16	0.98		
		IonCom	0.36	0.98	0.28	0.99		
		MIonSite	0.55	0.99	0.42	1.00		
		LMetalSite	0.51	**0.99**	0.36	1.00		
		LMetalSite calculated	0.47	0.99	0.27	1.00	0.45	0.89
		GASS-Metal (1)	0.08	0.98	0.09	0.99		
		GASS-Metal (2)	0.22	0.98	0.23	0.99		
		GASS-Metal (3)	0.28	0.98	0.29	0.99		
		M-Ionic	0.45	0.99	0.44	0.99		
		M-Ionic calculated	0.41	0.98	0.32	0.99	0.19	0.66
		AllMetal3D	0.48	0.99	0.30	1.00	0.30	0.66
		PT-LM-GNN	0.21	0.97	0.23	0.98	0.19	0.71
		AlphaFold3	0.61	0.99	0.43	1.00	0.52	0.79
		AlphaFold2+BiteNet_I	0.45	0.98	0.27	1.00	0.40	0.81
		BiteNet_I	**0.64**	0.99	0.54	1.00	**0.64**	0.91
		$\text{BiteNet}_\text{I}^*$	0.63	0.99	**0.57**	0.99	0.62	**0.91**

Mg^{2+}	93	MetalDetector	0.04	0.98	0.02	1.00		
		S-SITE	0.23	0.96	0.35	0.97		
		TargetS	0.30	0.99	0.15	1.00		
		IonSeq	NA	0.98	0.00	**1.00**		
		MIB	0.27	0.98	0.22	0.99		
		COACH	0.14	0.96	0.21	0.98		
		IonCom	0.28	0.98	0.24	0.99		
		MIonSite	0.36	0.99	0.24	1.00		
		LMetalSite	0.41	0.99	0.25	1.00		
		LMetalSite calculated	0.42	0.99	0.23	1.00	**0.41**	**0.90**
		GASS-Metal (1)	0.12	0.99	0.13	0.99		
		GASS-Metal (2)	0.33	0.99	0.34	0.99		
		GASS-Metal (3)	**0.55**	**0.99**	**0.56**	1.00		
		M-Ionic	0.38	0.99	0.29	1.00		
		M-Ionic calculated	0.40	0.99	0.33	1.00	0.17	0.66
		AllMetal3D	0.14	0.99	0.04	1.00	0.06	0.55
		PT-LM-GNN	0.17	0.97	0.24	0.98	0.17	0.69
		AlphaFold3	0.38	0.99	0.22	1.00	0.27	0.70
		AlphaFold2+BiteNet_I	0.26	0.99	0.12	1.00	0.20	0.78
		BiteNet_I	0.41	0.99	0.31	1.00	0.33	0.78
		$\text{BiteNet}_\text{I}^*$	0.41	0.99	0.28	1.00	0.29	0.81
Mn^{2+}	23	MetalDetector	0.32	0.99	0.21	1.00		
		S-SITE	0.49	0.98	0.69	0.98		
		TargetS	0.41	0.99	0.28	1.00		
		IonSeq	0.08	0.99	0.02	**1.00**		
		MIB	0.51	0.99	0.48	0.99		
		COACH	0.42	0.99	0.27	1.00		
		IonCom	0.53	0.99	0.54	0.99		
		MIonSite	0.56	0.99	0.57	0.99		
		LMetalSite	0.74	0.99	0.68	1.00		
		LMetalSite calculated	0.74	0.99	0.65	**1.00**	0.80	**0.98**
		GASS-Metal (1)	0.19	0.99	0.20	0.99		
		GASS-Metal (2)	0.43	0.99	0.44	1.00		
		GASS-Metal (3)	0.56	0.99	0.55	1.00		
		M-Ionic	0.68	0.99	0.58	1.00		
		M-Ionic calculated	0.68	0.99	0.63	1.00	0.47	0.81
		AllMetal3D	0.58	0.99	0.37	**1.00**	0.37	0.69
		PT-LM-GNN	0.58	0.99	0.56	0.99	0.50	0.90
		AlphaFold3	0.70	0.99	0.64	1.00	0.69	0.88
		AlphaFold2+BiteNet_I	0.60	0.99	0.54	1.00	0.52	0.91
		BiteNet_I	**0.81**	**1.00**	**0.85**	1.00	**0.85**	0.95
		$\text{BiteNet}_\text{I}^*$	0.75	0.99	0.70	1.00	0.76	0.95

		MetalDetector	0.36	0.99	0.29	1.00		
		S-SITE	0.56	0.99	0.91	0.99		
		TargetS	0.30	0.99	0.29	1.00		
		IonSeq	0.35	0.97	0.81	0.97		
		MIB	0.28	0.97	0.53	0.98		
		COACH	0.72	1.00	0.78	1.00		
		IonCom	0.76	1.00	0.77	1.00		
Fe^{3+}	7	MIonSite	0.77	1.00	0.83	1.00		
		LMetalSite	**0.93**	**1.00**	0.95	**1.00**		
		GASS-Metal (1)	0.73	**1.00**	0.73	**1.00**		
		GASS-Metal (2)	0.92	**1.00**	0.92	**1.00**		
		GASS-Metal (3)	0.92	**1.00**	0.92	**1.00**		
		M-Ionic	0.77	1.00	0.80	**1.00**		
		M-Ionic calculated	0.67	0.99	0.75	1.00	0.45	0.87
		AllMetal3D	0.39	0.99	0.15	**1.00**	0.16	0.57
		PT-LM-GNN	0.58	0.99	0.90	0.99	0.66	0.96
		AlphaFold3	0.72	1.00	0.70	1.00	0.70	0.90
		AlphaFold2+BiteNet_I	0.74	1.00	0.85	1.00	0.54	0.97
		BiteNet_I	0.86	1.00	**1.00**	1.00	**0.89**	**1.00**
		$\text{BiteNet}_\text{I}^*$	0.80	1.00	0.95	1.00	0.68	**1.00**
		MetalDetector	0.71	0.99	0.58	1.00		
		S-SITE	0.14	0.97	0.25	0.97		
		IonSeq	0.36	0.99	0.42	0.99		
		MIB	0.50	0.98	0.83	0.98		
		COACH	0.27	0.97	0.50	0.97		
		IonCom	0.52	0.99	0.50	1.00		
Cu^{2+}	3	MIonSite	0.52	0.99	0.48	1.00		
		LMetalSite	**0.90**	1.00	0.82	**1.00**		
		GASS-Metal (1)	0.67	1.00	0.67	**1.00**		
		GASS-Metal (2)	0.67	1.00	0.67	**1.00**		
		GASS-Metal (3)	0.67	1.00	0.67	**1.00**		
		M-Ionic	0.74	**1.00**	0.55	**1.00**		
		M-Ionic calculated	0.68	0.99	0.46	**1.00**	0.47	0.73
		AllMetal3D	0.68	0.99	0.54	1.00	0.48	0.77
		AlphaFold3	0.84	1.00	0.77	1.00	0.82	0.92
		AlphaFold2+BiteNet_I	0.83	1.00	0.83	1.00	0.88	1.00
		BiteNet_I	0.84	1.00	**1.00**	1.00	**0.97**	**1.00**
		$\text{BiteNet}_\text{I}^*$	0.81	0.99	**1.00**	0.99	0.94	1.00
		MetalDetector	0.50	0.99	0.33	1.00		
		S-SITE	0.31	0.95	0.67	0.95		
		IonSeq	0.78	0.99	0.98	0.99		
		MIB	0.83	0.99	**1.00**	0.99		
		COACH	0.44	0.98	0.67	0.98		
		IonCom	0.83	0.99	**1.00**	0.99		
Fe^{2+}	3	MIonSite	0.83	0.99	0.95	1.00		
		LMetalSite	**1.00**	**1.00**	**1.00**	**1.00**		
		GASS-Metal (1)	0.78	1.00	0.78	1.00		
		GASS-Metal (2)	0.89	1.00	0.89	1.00		
		GASS-Metal (3)	0.89	1.00	0.89	1.00		
		M-Ionic	**1.00**	**1.00**	**1.00**	**1.00**		
		M-Ionic calculated	**1.00**	**1.00**	**1.00**	**1.00**	**1.00**	**1.00**
		AllMetal3D	0.82	1.00	0.67	**1.00**	0.67	0.83
		AlphaFold3	**1.00**	**1.00**	**1.00**	**1.00**	**1.00**	**1.00**
		AlphaFold2+BiteNet_I	**1.00**	**1.00**	**1.00**	**1.00**	**1.00**	**1.00**
		BiteNet_I	**1.00**	**1.00**	**1.00**	**1.00**	**1.00**	**1.00**
		$\text{BiteNet}_\text{I}^*$	**1.00**	**1.00**	**1.00**	**1.00**	**1.00**	**1.00**

		MetalDetector	0.22	0.99	0.17	1.00		
		S-SITE	0.11	0.84	0.55	0.85		
		MIB	0.14	0.95	0.33	0.95		
		COACH	0.16	0.91	0.53	0.92		
		MIonSite	0.20	0.92	0.59	0.93		
Co^{2+}	5	LMetalSite	0.47	0.99	0.22	**1.00**		
		GASS-Metal (1)	**0.75**	**1.00**	**0.75**	**1.00**		
		GASS-Metal (2)	**0.75**	**1.00**	**0.75**	**1.00**		
		GASS-Metal (3)	**0.75**	**1.00**	**0.75**	**1.00**		
		M-Ionic	0.00	0.99	0.00	**1.00**		
		M-Ionic calculated	0.00	0.99	0.00	**1.00**	0.01	0.50
		AllMetal3D	0.47	0.99	0.22	**1.00**	0.23	0.60
		AlphaFold3	0.54	0.99	0.44	1.00	0.40	0.75
		AlphaFold2+BiteNet$_I$	0.28	0.99	0.22	1.00	0.18	0.76
		BiteNet$_I$	0.54	0.99	0.50	1.00	0.46	**0.86**
		BiteNet$_I^*$	0.58	0.99	0.50	1.00	**0.49**	0.82
		MetalDetector	NA	0.95	0.00	1.00		
		S-SITE	-0.08	0.84	0.00	0.89		
		IonSeq	-0.08	0.85	0.00	0.89		
Na^{+}	1	COACH	-0.06	0.89	0.00	0.94		
		IonCom	-0.09	0.82	0.00	0.86		
		MIonSite	0.08	0.90	0.14	0.94		
		AllMetal3D	0.83	0.98	0.83	0.99	0.81	0.99
		AlphaFold3	**1.00**	**1.00**	**1.00**	**1.00**	**1.00**	**1.00**
		AlphaFold2+BiteNet$_I$	0.00	0.95	0.00	1.00	0.28	0.93
		BiteNet$_I$	0.72	0.96	**1.00**	0.96	0.98	1.00
		BiteNet$_I^*$	0.52	0.92	0.83	0.93	0.90	0.99
		MetalDetector	-0.01	0.95	0.00	1.00		
		S-SITE	0.22	0.92	0.32	0.95		
		IonSeq	0.25	0.93	0.31	0.96		
K^{+}	3	COACH	0.30	0.94	0.35	0.97		
		IonCom	0.37	0.95	0.35	0.98		
		MIonSite	0.37	0.95	0.37	0.98		
		AllMetal3D	0.59	0.97	0.38	1.00	0.62	0.81
		AlphaFold3	0.63	0.97	0.46	1.00	0.48	0.73
		AlphaFold2+BiteNet$_I$	0.48	0.97	0.24	**1.00**	0.62	0.89
		BiteNet$_I$	**0.82**	**0.99**	**0.70**	1.00	**0.83**	0.96
		BiteNet$_I^*$	0.78	0.98	0.62	**1.00**	0.82	**0.97**
		MetalDetector	-0.01	0.96	0.00	**1.00**		
		S-SITE	0.04	0.89	0.14	0.92		
		MIB	0.19	0.91	**0.36**	0.93		
Cd^{2+}	2	COACH	0.06	0.95	0.07	0.98		
		MIonSite	0.10	0.95	0.09	0.98		
		AllMetal3D	0.00	0.97	0.00	**1.00**	0.03	0.50
		AlphaFold3	**0.39**	**0.97**	0.21	1.00	0.24	0.60
		BiteNet$_I$	0.27	0.95	0.29	0.98	**0.33**	**0.87**
		MetalDetector	0.70	0.98	0.50	1.00		
		S-SITE	-0.05	0.91	0.00	0.94		
		MIB	0.61	0.95	**1.00**	0.94		
Ni^{2+}	1	COACH	-0.04	0.92	0.00	0.95		
		MIonSite	0.34	0.96	0.25	0.99		
		AllMetal3D	**1.00**	**1.00**	**1.00**	**1.00**	**1.00**	**1.00**
		AlphaFold3	**1.00**	**1.00**	**1.00**	**1.00**	**1.00**	**1.00**
		AlphaFold2+BiteNet$_I$	**1.00**	**1.00**	**1.00**	**1.00**	**1.00**	**1.00**
		BiteNet$_I$	0.81	0.98	**1.00**	0.98	0.53	0.98
		BiteNet$_I^*$	0.81	0.98	**1.00**	0.98	0.69	0.99

Supplementary Table 7: Comparison of performance on the IonCom dataset (5-fold cross-validation). The reported metrics are taken from published results (MIonSite [1] and references therein where applicable). Entries for Metal3D, AllMetal3D, LMetalSite, M-Ionic and PT-LM-GNN were obtained by running the authors' released implementations/pretrained models and rescoring with our unified residue-level evaluation protocol (Section "Reference methods used for comparison" of the main text). BiteNet_I corresponds to the models trained on training sets of five folds of IonCom dataset, and $\text{BiteNet}^*_\text{I}$ is for the model trained on the BiteNet_I training set.

Ion	Structures	Method	MCC	Accuracy	Sensitivity	Specificity	AP	ROC AUC
Zn^{2+}	142	S-SITE [4]	0.38	0.98	0.56	0.98		
		TargetS [5]	0.49	0.99	0.35	**1.00**		
		IonSeq [6]	0.50	0.99	0.44	1.00		
		COACH [4]	0.50	0.99	0.57	0.99		
		IonCom [6]	0.59	1.00	0.49	**1.00**		
		MIonSite [1]	0.56	0.99	0.46	**1.00**		
		Metal3D [10]	0.49	0.98	**0.92**	0.98	0.48	0.98
		AllMetal3D [11]	0.54	0.99	0.59	1.00	0.37	0.83
		LMetalSite [8]	**0.82**	**1.00**	0.77	**1.00**	**0.88**	**1.00**
		M-Ionic [2]	0.77	1.00	0.80	1.00	0.60	0.90
		PT-LM-GNN [12]	0.47	0.99	0.74	0.99	0.64	0.87
		AlphaFold3 [13]	0.69	0.99	0.91	0.99	0.54	0.98
		AlphaFold2 [14]+BiteNet_I	0.71	1.00	0.80	1.00	0.80	0.98
		BiteNet_I	0.80±0.07	1.00±0.00	0.78±0.07	**1.00±0.00**	0.84±0.06	0.98±0.02
		$\text{BiteNet}^*_\text{I}$	0.74±0.07	1.00±0.00	0.85±0.08	1.00±0.00	0.85±0.08	0.99±0.01
Ca^{2+}	179	S-SITE	0.20	**1.00**	0.30	0.98		
		TargetS	0.20	0.98	0.26	0.99		
		IonSeq	0.21	0.98	0.23	0.99		
		COACH	0.20	0.97	0.32	0.98		
		IonCom	0.30	0.99	0.18	**1.00**		
		MIonSite	0.23	0.99	0.15	1.00		
		AllMetal3D	0.55	0.99	0.58	0.99	0.36	0.80
		LMetalSite	0.57	0.99	0.43	**1.00**	0.56	0.95
		M-Ionic	0.52	0.99	0.61	0.99	0.29	0.80
		PT-LM-GNN	0.43	0.98	0.58	0.99	0.48	0.85
		AlphaFold3	0.59	0.99	0.70	0.99	0.50	0.95
		AlphaFold2+BiteNet_I	0.54	0.99	0.50	1.00	0.56	0.94
		BiteNet_I	**0.74±0.05**	1.00±0.00	0.71±0.07	**1.00±0.00**	**0.76±0.05**	0.96±0.01
		$\text{BiteNet}^*_\text{I}$	0.67±0.05	0.99±0.00	**0.80±0.08**	0.99±0.00	0.74±0.10	**0.97±0.00**
Mg^{2+}	103	S-SITE	0.21	0.97	0.42	0.97		
		TargetS	0.19	1.00	0.09	1.00		
		IonSeq	0.18	1.00	0.06	**1.00**		
		COACH	0.28	0.98	0.45	0.98		
		IonCom	0.34	1.00	0.25	1.00		
		MIonSite	0.26	1.00	0.13	1.00		
		AllMetal3D	0.21	1.00	0.12	1.00	0.11	0.61
		LMetalSite	**0.50**	**1.00**	0.33	**1.00**	**0.45**	**0.92**
		M-Ionic	0.44	0.99	0.47	1.00	0.20	0.73
		PT-LM-GNN	0.27	0.99	0.47	0.99	0.31	0.74
		AlphaFold3	0.38	0.99	0.40	1.00	0.26	0.86
		AlphaFold2+BiteNet_I	0.27	0.99	0.22	1.00	0.20	0.86
		BiteNet_I	0.44±0.07	0.99±0.00	**0.47±0.08**	1.00±0.00	0.40±0.12	0.89±0.06
		$\text{BiteNet}^*_\text{I}$	0.42±0.07	0.99±0.00	0.46±0.12	1.00±0.00	0.34±0.09	0.92±0.04

Mn^{2+} 379	S-SITE	0.36	0.98	0.47	0.99		
	TargetS	0.49	0.99	0.42	1.00		
	IonSeq	0.46	0.99	0.31	**1.00**		
	COACH	0.47	0.99	0.54	0.99		
	IonCom	0.52	0.99	0.49	1.00		
	MIonSite	0.56	0.99	0.47	1.00		
	AllMetal3D	0.45	0.99	0.33	**1.00**	0.24	0.68
	LMetalSite	**0.75**	**1.00**	0.69	**1.00**	**0.78**	**0.98**
	M-Ionic	0.68	0.99	0.60	**1.00**	0.48	0.80
	PT-LM-GNN	0.67	0.99	0.66	1.00	0.59	0.86
	AlphaFold3	0.68	0.99	**0.82**	0.99	0.58	0.95
	AlphaFold2+BiteNet$_I$	0.56	0.99	0.43	**1.00**	0.53	0.92
	BiteNet$_I$	0.69±0.05	0.99±0.00	0.62±0.09	**1.00±0.00**	0.70±0.04	0.95±0.02
	BiteNet$_I^*$	0.68±0.03	0.99±0.00	0.60±0.05	**1.00±0.00**	0.66±0.01	0.95±0.01
Fe^{3+} 227	S-SITE	0.38	0.98	0.42	0.99		
	TargetS	0.62	0.99	0.54	1.00		
	IonSeq	0.64	0.99	0.52	**1.00**		
	COACH	0.66	0.99	0.62	1.00		
	IonCom	0.70	0.99	0.60	**1.00**		
	MIonSite	0.66	0.99	0.61	1.00		
	AllMetal3D	0.56	0.99	0.50	1.00	0.36	0.77
	M-Ionic	0.75	0.99	0.72	1.00	0.57	0.86
	PT-LM-GNN	0.69	0.99	0.79	0.99	0.72	0.90
	AlphaFold3	0.72	0.99	**0.86**	0.99	0.60	0.96
	AlphaFold2+BiteNet$_I$	0.72	0.99	0.62	**1.00**	0.70	0.95
	BiteNet$_I$	**0.78±0.03**	**0.99±0.00**	0.76±0.03	1.00±0.00	**0.80±0.05**	**0.97±0.02**
	BiteNet$_I^*$	0.77±0.04	**0.99±0.00**	0.71±0.05	**1.00±0.00**	0.77±0.06	0.96±0.02
Cu^{2+} 119	S-SITE	0.46	0.98	0.60	0.99		
	IonSeq	0.59	0.99	0.51	1.00		
	COACH	0.59	0.99	0.61	0.99		
	IonCom	0.68	0.99	0.53	**1.00**		
	MIonSite	0.69	0.99	0.55	**1.00**		
	AllMetal3D	0.58	0.99	0.59	0.99	0.43	0.83
	M-Ionic	0.72	0.99	0.70	1.00	0.53	0.85
	AlphaFold3	0.66	0.99	**0.84**	0.99	0.55	0.97
	AlphaFold2+BiteNet$_I$	0.74	0.99	0.78	1.00	0.73	0.95
	BiteNet$_I$	**0.77±0.02**	**0.99±0.00**	0.78±0.06	1.00±0.00	0.78±0.04	0.97±0.02
	BiteNet$_I^*$	0.71±0.04	0.99±0.00	0.82±0.02	0.99±0.00	**0.79±0.03**	**0.98±0.01**
Fe^{2+} 103	S-SITE	0.38	0.97	0.60	0.98		
	IonSeq	0.58	0.99	0.54	1.00		
	COACH	0.50	0.98	0.67	0.98		
	IonCom	0.58	0.99	0.60	0.99		
	MIonSite	0.58	0.99	0.60	0.99		
	AllMetal3D	0.60	0.99	0.60	1.00	0.41	0.81
	M-Ionic	0.79	1.00	0.70	**1.00**	0.63	0.85
	AlphaFold3	0.72	0.99	0.86	0.99	0.65	0.96
	AlphaFold2+BiteNet$_I$	0.73	0.99	0.66	1.00	0.73	0.98
	BiteNet$_I$	0.83±0.05	1.00±0.00	0.85±0.04	1.00±0.00	0.84±0.06	0.97±0.02
	BiteNet$_I^*$	**0.84±0.03**	**1.00±0.00**	**0.87±0.04**	1.00±0.00	**0.85±0.06**	**0.99±0.01**

Na^{+} 78	S-SITE	0.13	0.98	0.08	1.00		
	IonSeq	0.15	0.74	**0.77**	0.74		
	COACH	0.13	0.97	0.15	0.98		
	IonCom	0.18	0.92	0.43	0.93		
	AllMetal3D	0.31	0.98	0.21	**1.00**	0.17	0.63
	AlphaFold3	**0.54**	**0.98**	0.59	0.99	**0.46**	0.88
	AlphaFold2+BiteNet$_{I}$	0.36	0.98	0.31	0.99	0.32	0.88
	BiteNet$_{I}$	0.49±0.07	0.98±0.00	0.52±0.09	0.99±0.00	0.45±0.08	**0.92±0.03**
	BiteNet$_{I}^{*}$	0.49±0.09	0.98±0.01	0.54±0.15	0.99±0.01	0.43±0.09	**0.92±0.04**
K^{+} 53	S-SITE	0.06	0.97	0.04	0.99		
	IonSeq	0.23	0.97	0.09	**1.00**		
	COACH	0.08	0.94	0.13	0.96		
	IonCom	0.15	0.94	0.21	0.97		
	MIonSite	0.24	0.97	0.12	1.00		
	AllMetal3D	0.36	0.98	0.20	1.00	0.28	0.68
	AlphaFold3	**0.66**	**0.98**	**0.64**	0.99	**0.64**	0.90
	AlphaFold2+BiteNet$_{I}$	0.45	0.98	0.33	1.00	0.44	0.88
	BiteNet$_{I}$	0.53±0.15	0.98±0.01	0.50±0.23	0.99±0.01	0.60±0.16	**0.93±0.04**
CO_3^{2-} 62	S-SITE	0.09	0.98	0.06	1.00		
	IonSeq	0.21	**0.99**	0.11	**1.00**		
	COACH	0.14	0.98	0.09	1.00		
	IonCom	0.21	0.99	0.13	1.00		
	AlphaFold3	**0.38**	0.98	**0.47**	0.99	0.34	**0.90**
	BiteNet$_{I}$	0.37±0.17	0.99±0.00	0.33±0.13	0.99±0.00	**0.36±0.18**	0.88±0.05
NO_2^{-} 22	S-SITE	0.06	0.99	0.04	1.00		
	IonSeq	0.29	0.99	0.18	1.00		
	COACH	0.34	**0.99**	0.21	1.00		
	IonCom	**0.35**	**0.99**	0.17	**1.00**		
	AlphaFold3	0.30	0.99	0.21	1.00	**0.29**	**0.92**
	BiteNet$_{I}$	0.27±0.18	0.99±0.00	**0.25±0.20**	1.00±0.00	0.24±0.07	0.81±0.10
SO_4^{2-} 303	S-SITE	0.15	0.97	0.14	0.99		
	IonSeq	0.19	0.98	0.14	0.99		
	COACH	0.21	0.97	0.19	0.99		
	IonCom	0.23	0.98	0.15	1.00		
	M-Ionic	0.00	0.98	0.00	**1.00**	0.02	0.50
	AlphaFold3	0.43	0.97	0.49	0.99	0.34	0.89
	AlphaFold2+BiteNet$_{I}$	0.32	0.98	0.24	0.99	0.28	0.86
	BiteNet$_{I}$	**0.50±0.03**	0.98±0.00	**0.56±0.05**	0.99±0.00	**0.48±0.04**	**0.94±0.01**
	BiteNet$_{I}^{*}$	0.47±0.05	**0.98±0.00**	0.44±0.06	0.99±0.00	0.45±0.06	0.93±0.01
PO_4^{3-} 339	S-SITE	0.27	0.97	0.28	0.99		
	IonSeq	0.31	0.98	0.24	0.99		
	COACH	0.34	0.98	0.35	0.99		
	IonCom	0.37	0.98	0.32	0.99		
	M-Ionic	0.44	0.98	0.34	**1.00**	0.21	0.67
	AlphaFold3	0.51	0.98	**0.65**	0.98	0.46	**0.94**
	AlphaFold2+BiteNet$_{I}$	0.40	0.98	0.28	**1.00**	0.37	0.88
	BiteNet$_{I}$	**0.59±0.03**	**0.98±0.00**	0.63±0.04	0.99±0.00	**0.56±0.05**	**0.94±0.01**
	BiteNet$_{I}^{*}$	0.52±0.03	**0.98±0.00**	0.45±0.04	1.00±0.00	0.52±0.01	0.93±0.01

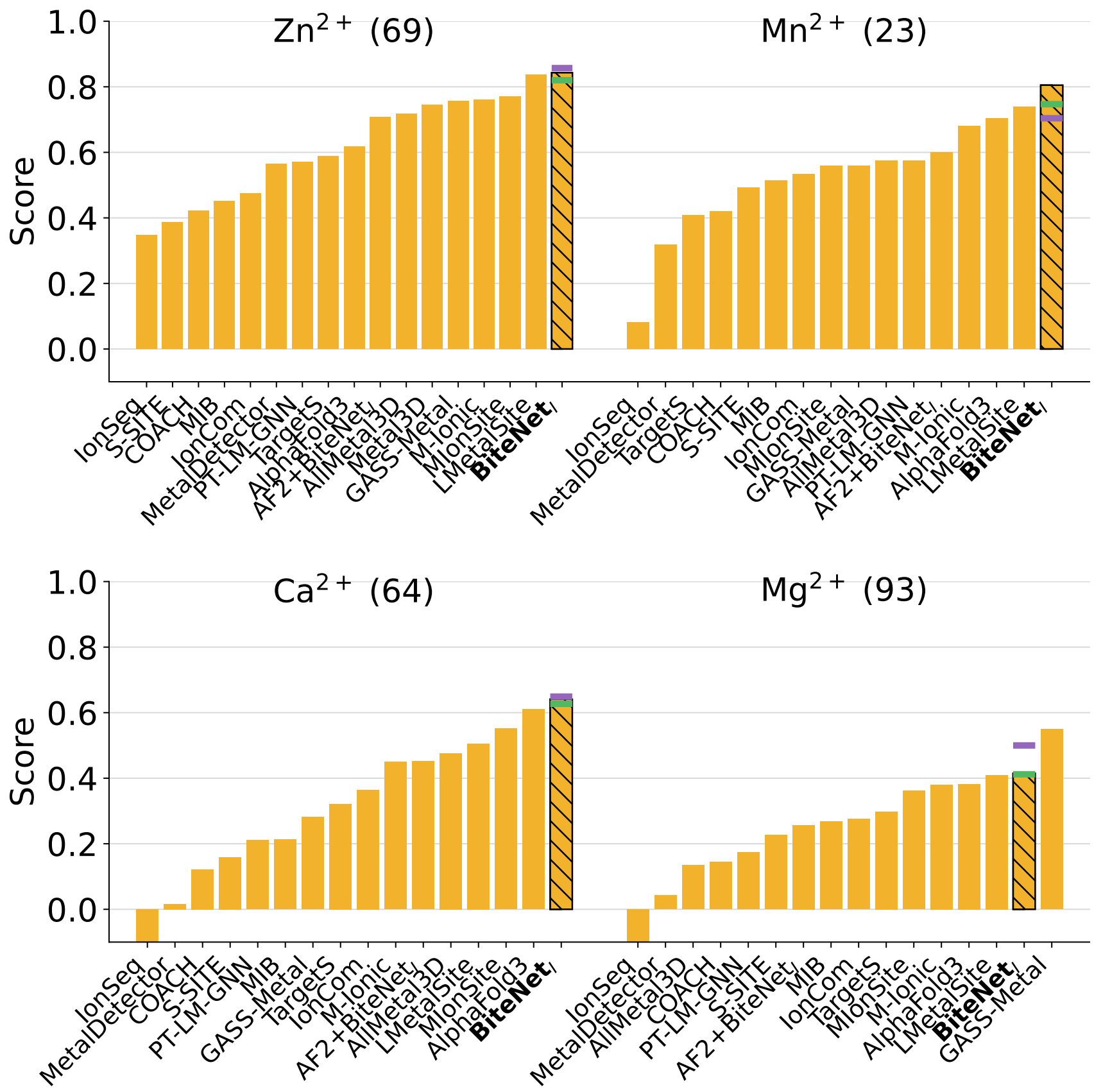

Supplementary Figure 5: **Comparison of the MCC scores of different methods on the MIonSite benchmark for Zn^{2+}, Mn^{2+}, Ca^{2+}, Mn^{2+}.** Values in parentheses in each panel's title indicate the number of benchmark structures for the corresponding ion class (n). Green horizontal lines represent scores on MIonSite validation set of BiteNet$_I$ model, trained on the BiteNet$_I$ train set. Similarly, violet horizontal lines represent weighted MCC score of BiteNet$_I$ on the test set. Note, that here we show scores only for ion types with number of structures ≥ 20 in MIonSite validation set. Supplementary Tables 6 and 7 demonstrate more detailed information on performance metrics.

Supplementary Table 8: Performance of Metal3D and $BiteNet_I$ on the Metal3D Zn^{2+} binding site test set (54 assemblies, 132 Zn^{2+} binding sites after filtering).

Method	TP	FN	FP	Precision	Recall	F1	MAD (mean)	MAD (median)	AP
BiteNetI, min_score=0.0	129	3	1031	0.111	0.977	0.200	0.523	0.299	0.822
BiteNet, min_score=0.1	116	16	191	0.378	0.879	0.528	0.493	0.277	0.797
BiteNet, min_score=0.2	110	22	104	0.514	0.833	0.636	0.550	0.255	0.769
BiteNet, min_score=0.3	106	26	62	0.631	0.803	0.707	0.542	0.250	0.752
BiteNet, min_score=0.4	105	27	34	0.755	0.795	0.775	0.537	0.249	0.747
BiteNet, min_score=0.5	103	29	24	0.811	0.780	0.795	0.536	0.235	0.735
BiteNetI, min_score=0.6	103	29	16	0.866	0.780	0.821	0.556	0.235	0.729
BiteNetI, min_score=0.7	101	31	10	0.910	0.765	0.831	0.546	0.233	0.715
BiteNetI, min_score=0.8	99	33	6	0.943	0.750	0.835	0.544	0.233	0.701
BiteNetI, min_score=0.9	86	46	1	0.989	0.652	0.785	0.394	0.211	0.628
BiometAll	55	77	100	0.355	0.417	0.383	2.200	1.860	0.098
MIB, min_score=1.25	71	61	162	0.305	0.538	0.389	0.919	0.427	0.157
MIB, min_score=1.9	41	91	3	0.932	0.311	0.466	0.573	0.228	0.131
MIB2, min_score=2.5	80	52	132	0.377	0.606	0.465	0.416	0.235	0.142
MIB2, min_score=4.0	41	91	34	0.547	0.311	0.396	0.756	0.285	0.107
Metal3D, min_score=0.0	98	34	213	0.315	0.742	0.442	0.694	0.520	0.633
Metal3D, min_score=0.25	93	39	71	0.567	0.705	0.628	0.635	0.497	0.617
Metal3D, min_score=0.5	90	42	28	0.763	0.682	0.720	0.606	0.480	0.603
Metal3D, min_score=0.75	75	57	8	0.904	0.568	0.698	0.634	0.398	0.512
Metal3D, min_score=0.9	61	71	1	0.984	0.462	0.629	0.630	0.356	0.421

Supplementary Table 9: Performance of Metal3D and $BiteNet_I$ on the non-redundant subset of the Metal3D Zn^{2+} binding site test set (23 assemblies, 51 Zn^{2+} sites after filtering and overlap removal).

Method	TP	FN	FP	Precision	Recall	F1	MAD (mean)	MAD (median)	AP
BiteNet, min_score=0.0	51	0	564	0.083	1.000	0.153	0.511	0.216	0.861
BiteNetI, min_score=0.1	50	1	102	0.329	0.980	0.493	0.537	0.216	0.854
BiteNetI, min_score=0.2	48	3	60	0.444	0.941	0.604	0.699	0.215	0.822
BiteNet, min_score=0.3	47	4	38	0.553	0.922	0.691	0.676	0.214	0.812
BiteNet, min_score=0.4	47	4	20	0.701	0.922	0.797	0.676	0.214	0.812
BiteNetI, min_score=0.5	47	4	13	0.783	0.922	0.847	0.676	0.214	0.812
BiteNetI, min_score=0.6	47	4	9	0.839	0.922	0.879	0.676	0.214	0.812
BiteNetI, min_score=0.7	46	5	5	0.902	0.902	0.902	0.659	0.214	0.795
BiteNetI, min_score=0.8	45	6	3	0.938	0.882	0.909	0.671	0.214	0.777
BiteNetI, min_score=0.9	35	16	1	0.972	0.686	0.805	0.296	0.188	0.666
BioMetAll	26	25	44	0.371	0.510	0.430	2.038	1.921	0.134
MIB, min_score=1.25	27	24	77	0.260	0.529	0.348	0.776	0.334	0.210
MIB, min_score=1.9	14	37	3	0.824	0.275	0.412	0.238	0.122	0.177
MIB2, min_score=2.5	38	13	67	0.362	0.745	0.487	0.517	0.323	0.149
MIB2, min_score=4.0	17	34	24	0.415	0.333	0.370	0.723	0.513	0.093
Metal3D, min_score=0.0	37	14	100	0.270	0.725	0.394	0.623	0.395	0.652
Metal3D, min_score=0.25	37	14	30	0.552	0.725	0.627	0.623	0.395	0.652
Metal3D, min_score=0.5	35	16	14	0.714	0.686	0.700	0.552	0.390	0.629
Metal3D, min_score=0.75	32	19	0	1.000	0.627	0.771	0.509	0.311	0.583
Metal3D, min_score=0.9	30	21	0	1.000	0.588	0.741	0.601	0.311	0.531

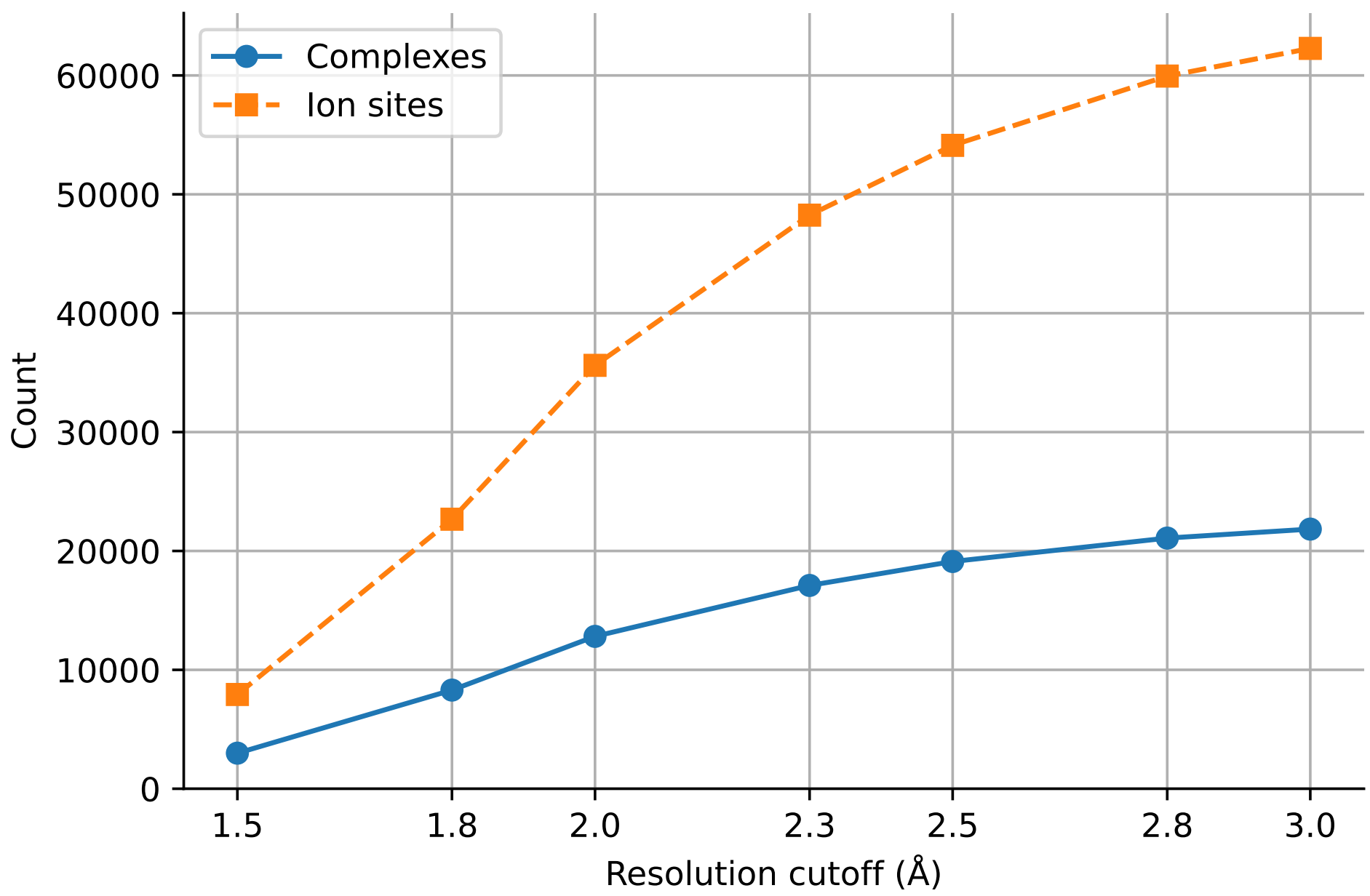

Supplementary Figure 6: **Number of protein–ion complexes and ion binding sites in the curated dataset as a function of the maximum resolution cutoff.** Cumulative counts are shown for 1.5, 1.8, 2.0, 2.3, 2.5, 2.8, and 3.0 Å.

Supplementary Table 10: Number of occurrences of relevant ions of specific type in the dataset. The numbers of structures containing this ion and the number of such ions in structures with ≤ of specified resolution are shown on the left and right, respectively. Ion names correspond to their identifiers in PDB.

	Structures							Ions						
Ion	1.5	1.8	2.0	2.3	2.5	2.8	3.0	1.5	1.8	2.0	2.3	2.5	2.8	3.0
	2992	8298	12816	17101	19114	21083	21839	7928	22670	35613	48248	54117	59946	62274
2FK	0	0	0	1	1	1	1	0	0	0	1	1	1	1
2HP	1	2	4	5	6	7	7	1	2	4	5	6	7	7
3CO	0	1	1	1	1	1	1	0	1	1	1	1	1	1
3NI	2	2	3	3	3	4	4	4	4	6	6	6	7	7
3P8	0	0	0	0	0	1	1	0	0	0	0	0	1	1
ACT	250	559	797	1010	1070	1122	1134	611	1220	1745	2244	2387	2508	2535
AG	0	2	2	5	6	9	9	0	7	7	20	21	34	34
AL	0	0	0	0	1	1	1	0	0	0	0	4	4	4
ALF	0	0	0	2	2	5	8	0	0	0	4	4	7	10
AU	2	4	8	13	14	17	18	3	7	18	28	32	44	46
AU3	0	1	1	1	1	1	1	0	4	4	4	4	4	4
AUC	0	0	1	1	1	1	1	0	0	2	2	2	2	2
AZI	4	15	20	33	37	37	37	19	39	46	72	81	81	81
BA	0	1	7	10	12	15	16	0	2	14	21	25	31	33
BCT	4	13	21	27	30	33	34	6	24	41	56	67	70	72
BEF	1	9	19	27	32	36	36	1	12	28	40	50	56	56
BO4	0	0	0	1	1	1	1	0	0	0	1	1	1	1
BR	6	29	55	72	79	84	86	9	89	145	187	204	227	230
BS3	0	1	1	1	1	1	1	0	1	1	1	1	1	1
CA	448	1303	2027	2710	3017	3286	3403	835	2626	4141	5616	6321	6947	7246
CAC	12	26	36	49	57	61	64	26	64	92	118	141	146	150
CD	12	48	82	106	114	129	135	22	148	261	344	368	411	429
CL	469	1201	1841	2475	2770	3006	3097	939	2568	3948	5302	5893	6409	6646
CO	32	90	131	162	178	192	195	102	230	327	450	492	533	541
CO3	7	25	41	58	69	70	70	15	52	74	97	116	117	117
CON	1	1	1	1	1	1	1	1	1	1	1	1	1	1
CS	1	11	16	24	31	35	36	4	37	52	82	114	133	141
CU	88	235	330	409	437	457	461	221	613	880	1068	1136	1181	1192
CU1	23	50	63	68	70	74	74	35	75	99	105	110	116	116
CUA	0	0	2	3	3	3	3	0	0	3	4	4	4	4
CYN	3	5	5	8	8	8	8	10	17	17	26	26	26	26
EMC	1	4	6	8	10	12	12	3	9	14	18	29	32	32
ER3	0	0	0	0	0	1	1	0	0	0	0	0	2	2
EU	0	0	0	1	1	1	1	0	0	0	3	3	3	3
EU3	1	2	3	3	3	3	3	1	3	4	4	4	4	4
F	2	4	4	5	6	13	18	3	11	11	15	17	38	54
FE	81	203	319	419	466	522	544	143	451	721	989	1084	1193	1241
FE2	36	79	116	155	178	205	215	77	207	306	400	468	545	565
GA	0	2	2	2	2	2	2	0	2	2	2	2	2	2
GD3	0	1	1	1	3	3	3	0	1	1	1	5	5	5
HG	14	39	67	91	102	120	126	31	78	129	207	230	281	290
HOH	0	0	0	1	1	1	1	0	0	0	2	2	2	2
IOD	17	30	46	66	78	91	95	144	184	219	278	320	371	379
IR	0	0	0	1	1	1	1	0	0	0	1	1	1	1
IR3	0	0	1	1	1	3	3	0	0	2	2	2	5	5
IUM	1	2	2	3	3	3	3	4	7	7	9	9	9	9
K	53	194	319	461	533	603	631	104	392	700	1061	1239	1425	1503
LA	0	0	0	0	0	1	1	0	0	0	0	0	2	2
LCP	0	1	1	1	2	2	2	0	1	1	1	2	2	2
LI	6	10	16	19	21	23	23	8	13	20	27	33	38	38
LU	0	0	0	1	2	2	2	0	0	0	1	2	2	2
MAC	0	1	1	1	1	1	1	0	2	2	2	2	2	2
MG	236	704	1095	1485	1669	1876	1953	460	1640	2560	3495	3888	4366	4525
MH2	2	4	4	4	4	4	4	4	8	8	8	8	8	8
MMC	1	2	3	4	4	4	4	1	3	4	10	10	10	10
MN	49	176	334	498	583	684	726	125	574	1058	1515	1789	2071	2193

	1.5	1.8	2.0	2.3	2.5	2.8	3.0	1.5	1.8	2.0	2.3	2.5	2.8	3.0
MN3	2	4	5	6	8	9	10	5	11	13	17	24	28	30
MOO	1	4	8	9	11	12	12	1	8	24	25	35	40	40
NA	368	1150	1641	2093	2278	2461	2514	707	2118	3140	4119	4507	4898	5029
NH4	6	23	35	40	44	48	48	13	58	76	89	98	109	109
NI	49	139	223	280	321	361	378	88	280	430	565	647	734	759
NO2	10	24	31	33	33	33	33	26	69	87	91	91	91	91
NO3	28	85	119	141	157	167	169	57	173	263	311	354	383	388
OH	12	19	28	34	37	40	43	14	29	50	59	64	67	72
OS	0	0	1	1	1	1	1	0	0	6	6	6	6	6
OS4	0	0	0	0	1	1	1	0	0	0	0	2	2	2
PB	2	5	8	11	11	12	14	3	12	18	23	23	24	32
PD	0	2	3	3	4	4	4	0	2	6	6	9	9	9
PER	2	2	5	5	7	7	7	2	2	5	5	7	7	7
PI	3	3	3	5	5	5	5	6	6	6	10	10	10	10
PO3	4	5	5	8	8	9	9	6	7	7	12	12	13	13
PO4	207	562	891	1208	1369	1551	1604	412	1198	1975	2777	3170	3597	3734
PR	1	4	6	8	8	10	10	2	8	11	14	14	24	24
PT	1	7	8	12	17	21	22	1	7	8	14	23	31	32
PT4	0	0	4	4	4	4	4	0	0	17	17	17	17	17
RB	0	2	2	3	6	8	8	0	8	8	11	18	29	29
RU	0	1	1	1	2	2	2	0	1	1	1	3	3	3
SCN	11	25	44	51	54	58	60	26	66	112	125	131	143	145
SE4	2	3	3	3	3	3	3	2	7	7	7	7	7	7
SM	1	2	2	2	3	4	4	1	5	5	5	7	8	8
SO3	2	5	7	10	12	13	13	8	11	15	20	27	30	30
SO4	639	1758	2789	3772	4212	4627	4795	1838	4955	7845	10780	12079	13361	13935
SR	1	3	5	5	6	7	9	1	7	10	10	12	16	20
TB	0	0	0	1	1	2	2	0	0	0	3	3	5	5
TL	0	0	1	1	1	2	3	0	0	4	4	4	6	18
TMA	1	1	2	2	2	2	2	5	5	6	6	6	6	6
V	0	1	2	2	2	2	2	0	1	2	2	2	2	2
VN3	0	1	1	3	3	3	3	0	3	3	6	6	6	6
VO4	1	6	10	21	22	24	26	1	8	17	34	35	37	41
WO4	1	9	17	20	21	21	26	2	11	26	31	32	32	46
Y1	0	0	1	2	2	2	2	0	0	1	2	2	2	2
YB	0	1	3	5	6	7	8	0	2	7	9	17	23	24
YT3	0	0	0	1	3	4	4	0	0	0	1	3	4	4
ZN	407	1170	1885	2518	2837	3199	3347	729	2163	3676	5074	5842	6606	6925

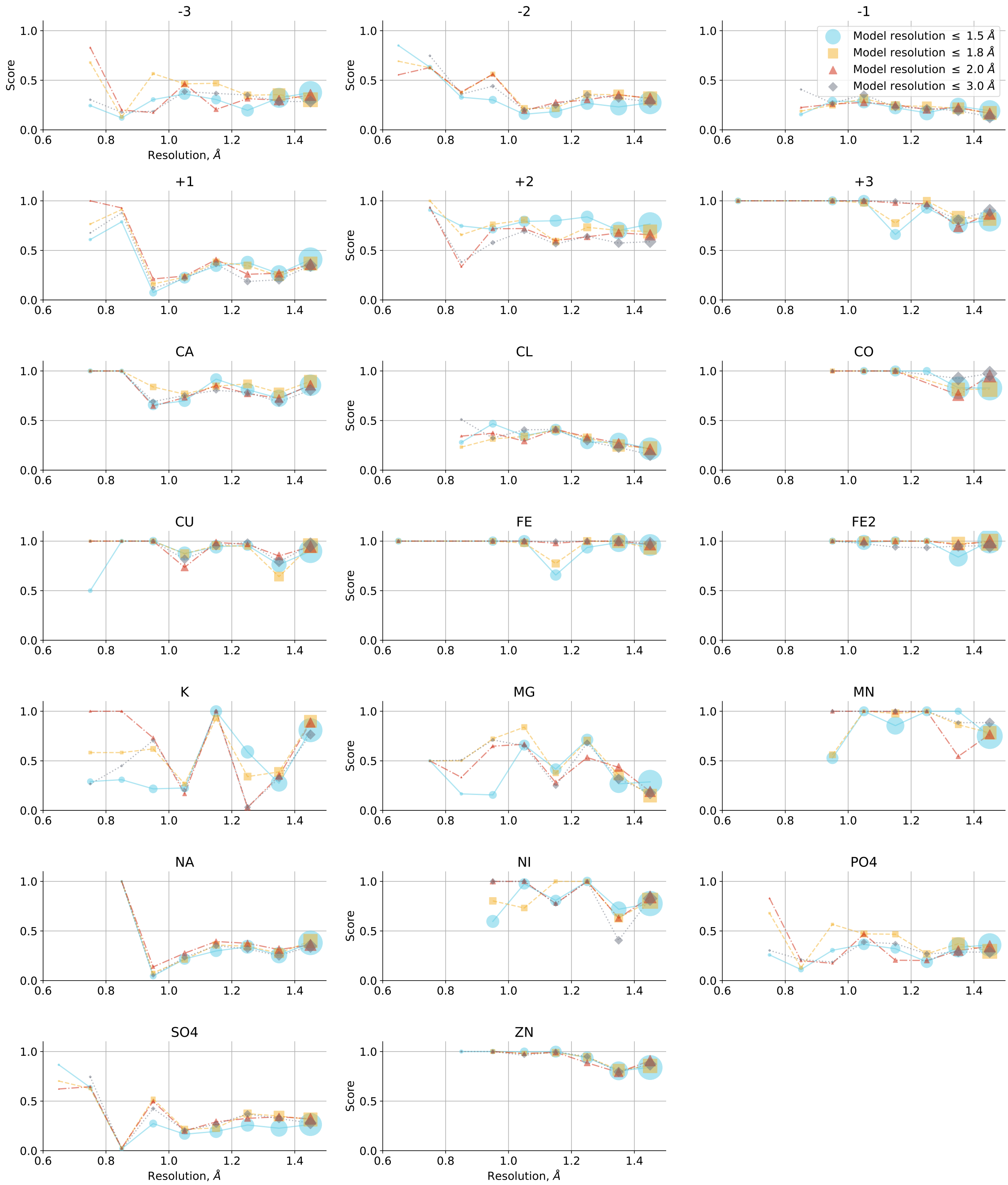

Supplementary Figure 7: **The AP scores of BiteNet$_{\mathrm{I}}$ models for different ion classes as a function of resolution of experimental structures.** The four models were trained using structures with different resolution thresholds: ≤ 1.5 Å (cyan circles, solid line), ≤ 1.8 Å (orange squares, dashed line), ≤ 2.0 Å (red triangles, dash-dotted line), and ≤ 3.0 Å (gray diamonds, dotted line). Each point (x, y) corresponds to the AP score (y) calculated for the indicated ion class on validation structures whose resolution falls within the corresponding bin. Marker sizes are scaled according to the number of validation structures in each bin. The exact number of validation structures used to calculate each point is provided in Source Data Supplementary Fig. 7 in the `num_structures` column.

Supplementary Table 11: Performance of $\mathrm{BiteNet_I}$ on high-resolution cryo-EM structures. We selected PDB entries with experimental method *ELECTRON MICROSCOPY*, reported resolution $\leq$ 3.0 Å, deposited before 1 January 2025, and containing at least one of the ion types considered by $\mathrm{BiteNet_I}$ (1051 structures total). The pre-trained model (trained on crystallographic structures) was applied without any retraining. We report center-based Average Precision ($\mathrm{AP_{center}}$; matching predicted site centers to ground truth), as well as residue-level Average Precision (AP), ROC AUC, and Matthews correlation coefficient (MCC). N_{str} is the number of cryo-EM structures containing the corresponding class/ion (structures can contribute to multiple rows).

Class / Ion	N_{str}	$\mathrm{AP_{center}}$	AP	ROC AUC	MCC
-3	58	0.426	0.339	0.816	0.442
-2	7	0.226	0.177	0.809	0.269
-1	146	0.124	0.111	0.822	0.170
+1	130	0.506	0.468	0.896	0.443
+2	818	0.671	0.624	0.940	0.597
+3	50	0.674	0.591	0.992	0.564
CA	246	0.838	0.804	0.961	0.756
CL	98	0.164	0.109	0.744	0.198
CO	4	0.987	0.848	0.939	0.638
CU	21	0.680	0.530	0.951	0.429
FE	47	0.669	0.610	0.991	0.574
FE2	7	0.898	0.820	0.975	0.731
K	85	0.501	0.501	0.875	0.470
MG	374	0.292	0.238	0.905	0.283
MN	22	0.884	0.676	0.954	0.575
NA	41	0.294	0.257	0.912	0.352
NI	2	0.727	0.300	0.820	0.409
PO4	53	0.422	0.339	0.819	0.436
SO4	3	0.269	0.154	0.699	0.139
ZN	230	0.944	0.771	0.973	0.643

Supplementary Table 12: Comparison of models trained on dataset with structural water present and without it. Mean Average Precision and Average Precision scores for each ion class separately are shown. Model "with water, O.3 channel" represent model trained on dataset with structural water, where oxygens from water are included into O.3 protein channel; and model in "with water, separate channel" additional channel for water oxygen is added. And "water in validation set" denote, whether structural water in structures was considered in validation.

Index	Model	Water in validation set	mAP	-3	-2	-1	+1	+2	+3
1	$\mathrm{BiteNet_I}$ no water	No	0.58	0.38	0.33	0.21	0.37	0.73	0.77
2	With water, O.3 channel	No	0.52	0.32	0.28	0.11	0.29	0.66	0.73
3	With water, O.3 channel	Yes	0.69	0.59	0.54	0.46	0.55	0.80	0.76
4	With water, separate channel	No	0.52	0.30	0.28	0.11	0.29	0.67	0.77
5	With water, separate channel	Yes	0.70	0.59	0.55	0.47	0.55	0.81	0.80

Index	CA	CL	CO	CU	FE	FE2	K	MG	MN	NA	NI	PO4	SO4	ZN
1	0.73	0.26	0.87	0.85	0.87	0.90	0.55	0.51	0.92	0.38	0.77	0.38	0.32	0.89
2	0.66	0.12	0.85	0.83	0.82	0.87	0.50	0.33	0.85	0.28	0.76	0.32	0.28	0.89
3	0.83	0.56	0.86	0.85	0.85	0.81	0.79	0.65	0.95	0.55	0.78	0.59	0.55	0.91
4	0.67	0.13	0.85	0.85	0.83	0.86	0.48	0.33	0.87	0.29	0.78	0.30	0.28	0.89
5	0.82	0.56	0.87	0.87	0.87	0.84	0.76	0.66	0.95	0.56	0.82	0.59	0.55	0.90

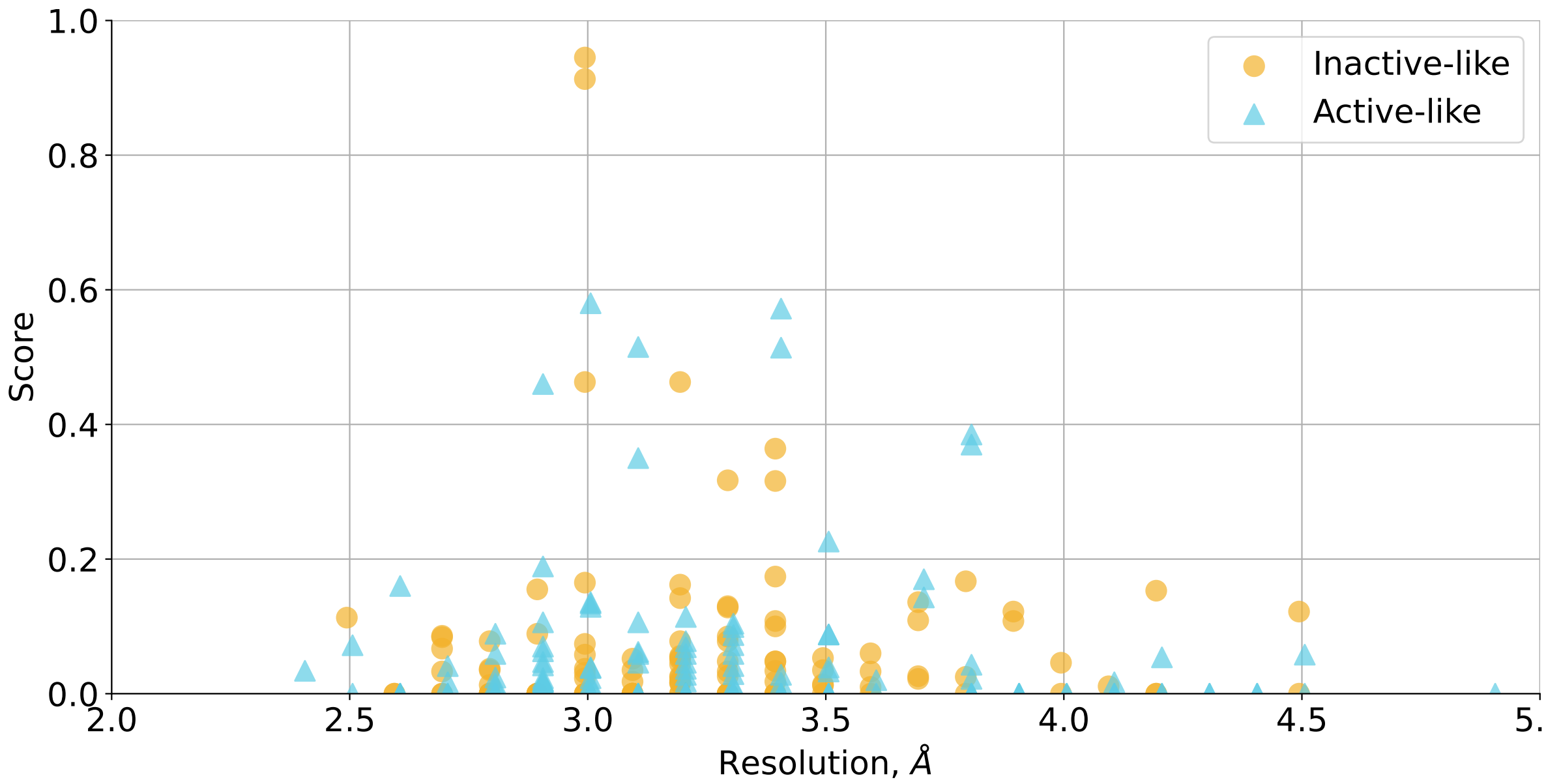

Supplementary Figure 8: **Scatter plot with the prediction scores corresponding to sodium pocket in class A GPCR structures without resolved water molecules.** Predictions for inactive and active structures are shown as orange circles and cyan triangles, respectively.

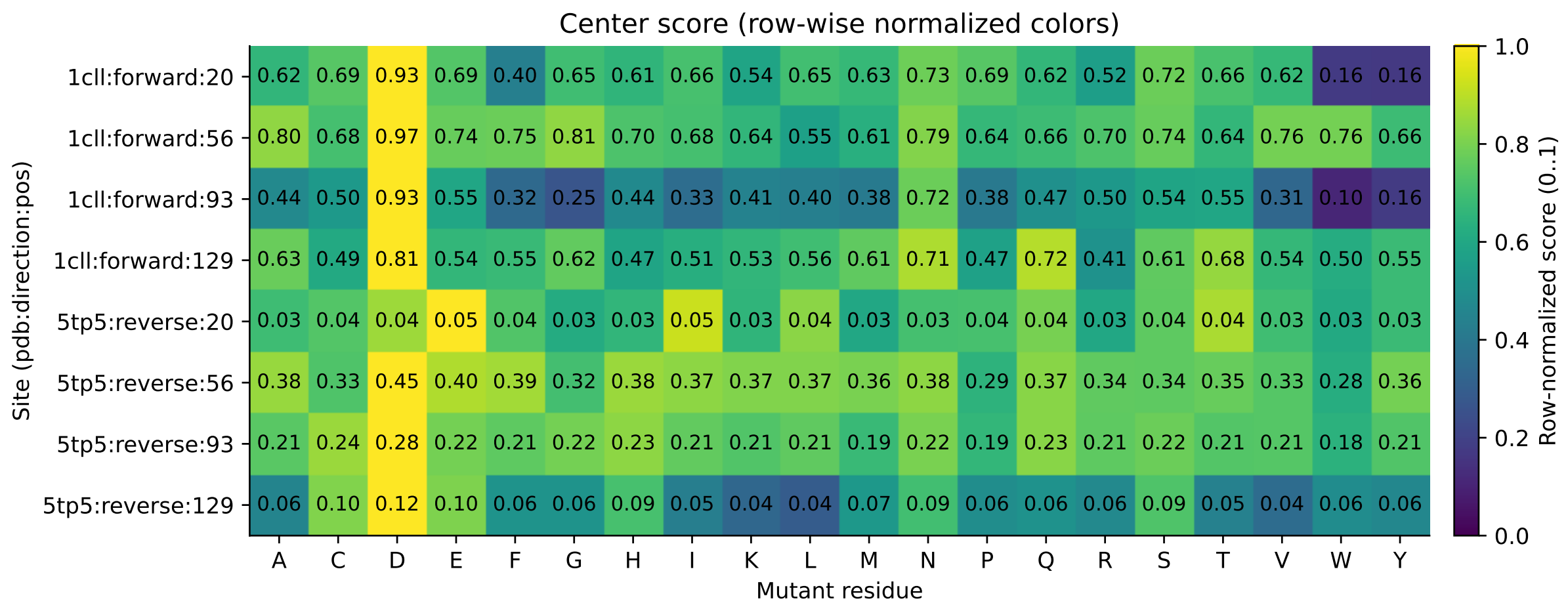

Supplementary Figure 9: **Point mutation analysis of CaM EF-hand Ca^{2+} binding sites.** For each EF-hand site (positions 20/56/93/129) we modeled all 20 amino-acid substitutions in two backgrounds: (i) 1CLL (forward scan D→X; each number is the mean over $n = 20$ modelled structures per substitution, including D→D) and (ii) 5TP5 (reverse scan A→X; each number is the mean over $n = 400$ modelled structures per substitution, corresponding to 20 NMR conformers × 20 MODELLER models, including A→A). MODELLER remodeling was restricted to residues within ±2 positions in sequence and within 6 Å of the mutated residue (`library_schedule=slow`, `md_level=slow_large`). All ions were removed prior to point mutation modeling. For each structure and each site, we assigned the score of the closest predicted Ca^{2+} center within 5 Å of a site center defined from the coordinating atoms in 1CLL. Colors are normalized with respect to each row.

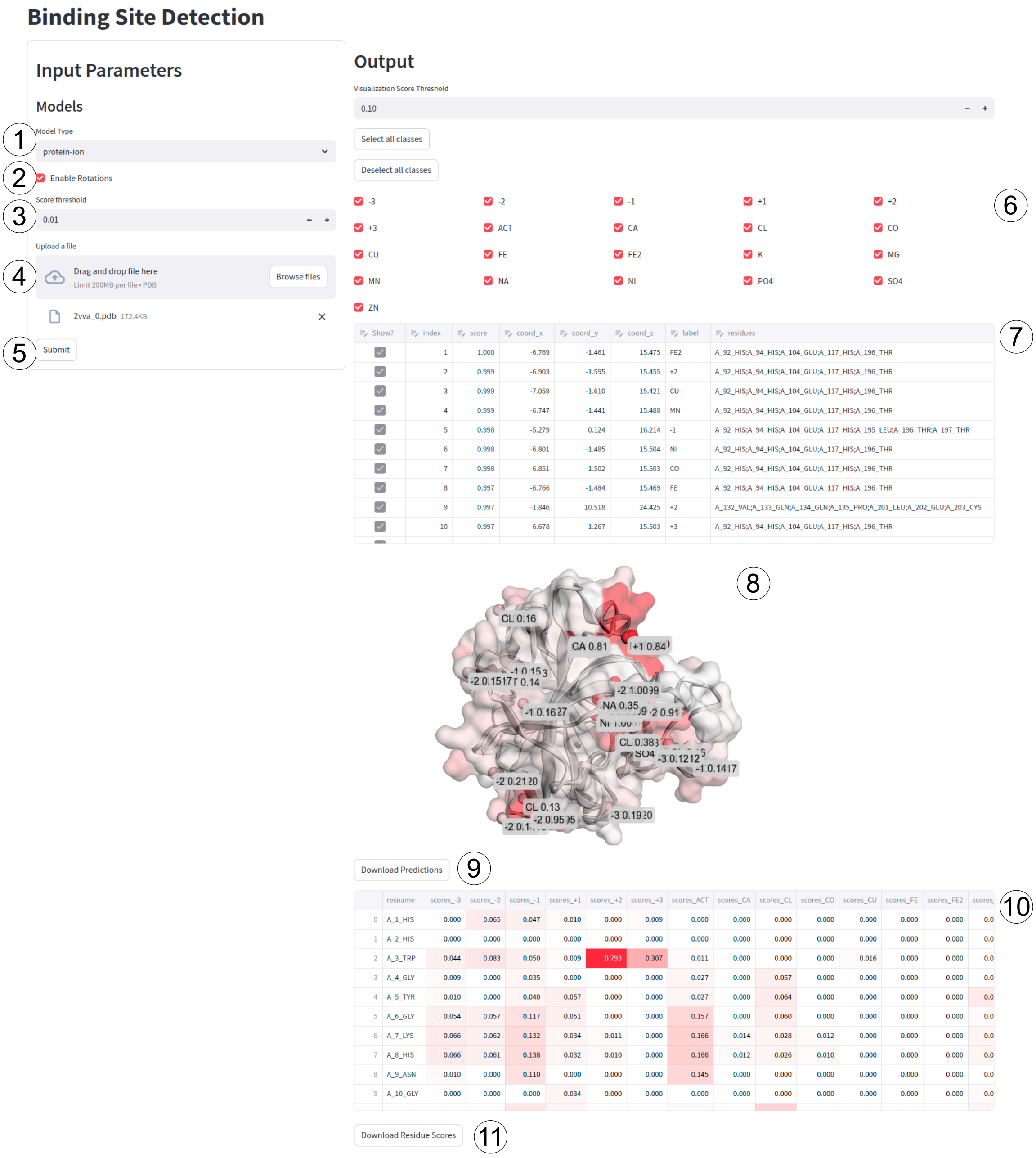

Supplementary Figure 10: **Streamlit web app for BiteNet$_I$.** (1) Selection of the BiteNet$_I$ model; (2) An augmentation option (takes longer times); (3) The score-threshold slider; (4) PDB upload widget; (5) "Submit" button; (6) Selection of the ion classes of interest; (7) The output table with binding site predictions; (8) Interactive py3Dmol viewer with predictions displayed; (9) "Download Predictions" button; (10) The output heat-map that highlights the binding site residues; (11) "Download Residue Scores" button.

Supplementary Table 13: Number of target ions of different types in the initial dataset and after the addition of the aligned from other structures.

Ion	Initial	Added	Final
	35612	40035	75647
2HP	4	8	12
3CO	1	174	175
3NI	6	42	48
ACT	1745	4361	6106
AG	7	52	59
AU	18	8	26
AU3	4	16	20
AZI	46	462	508
BA	14	60	74
BCT	41	223	264
BEF	28	1	29
BR	145	300	445
BS3	1	28	29
CA	4141	1689	5830
CAC	92	29	121
CD	261	302	563
CL	3948	7244	11192
CO	327	910	1237
CO3	74	233	307
CON	1	5	6
CS	52	250	302
CU	880	402	1282
CU1	99	351	450
CUA	3	0	3
CYN	17	32	49
EMC	14	3	17
EU3	4	1	5
F	11	47	58
FE	721	387	1108
FE2	306	268	574
GA	2	7	9
GD3	1	1	2
HG	129	722	851
IOD	219	387	606
IR3	2	1	3
IUM	7	0	7
K	700	560	1260
LCP	1	0	1
LI	20	45	65
MAC	2	0	2
MG	2560	1377	3937
MH2	8	79	87
MMC	4	71	75
MN	1060	747	1807
MN3	13	76	89
MOO	24	11	35
NA	3141	3812	6953
NH4	76	137	213
NI	430	725	1155
NO2	87	352	439
NO3	263	421	684
OH	50	262	312
OS	6	16	22

PB	18	8	26
PD	6	4	10
PER	5	59	64
PI	6	2	8
PO3	7	7	14
PO4	1975	1969	3944
PR	11	96	107
PT	8	37	45
PT4	17	103	120
RB	8	74	82
RU	1	14	15
SCN	112	61	173
SE4	7	72	79
SM	5	1	6
SO3	15	211	226
SO4	7845	7968	15813
SR	10	6	16
TL	4	72	76
TMA	6	4	10
V	2	3	5
VN3	3	0	3
VO4	17	7	24
WO4	26	8	34
Y1	1	0	1
YB	7	23	30
ZN	3674	1529	5203

X-ray versus cryo-EM metal-binding sites in the PDB

To compare the availability of metal-binding sites in X-ray and cryo-electron microscopy (cryo-EM) structures, we analysed all PDB entries deposited before 1 January 2025 that are available in legacy PDB format. For each entry, we recorded the experimental method ("X-RAY DIFFRACTION" or "ELECTRON MICROSCOPY"), the nominal resolution, and the set of HET groups present. We considered the same set of ion types as in the resolution analysis (Supplementary Table 10). For each method and resolution bin (≤ 2.0, 2.0–2.5, 2.5–3.0, 3.0–3.5, > 3.5 Å) we counted how many structures contained at least one of these ions (Supplementary Figure 11). X-ray structures with at least one selected ion outnumber cryo-EM structures with ions by more than an order of magnitude in all resolution ranges. For example, at resolutions ≤ 2.5 Å we observed 79,578 X-ray structures with at least one selected ion versus 511 cryo-EM structures with ions; at resolutions ≤ 3.0 Å these numbers were 92,671 and 2,106, respectively.

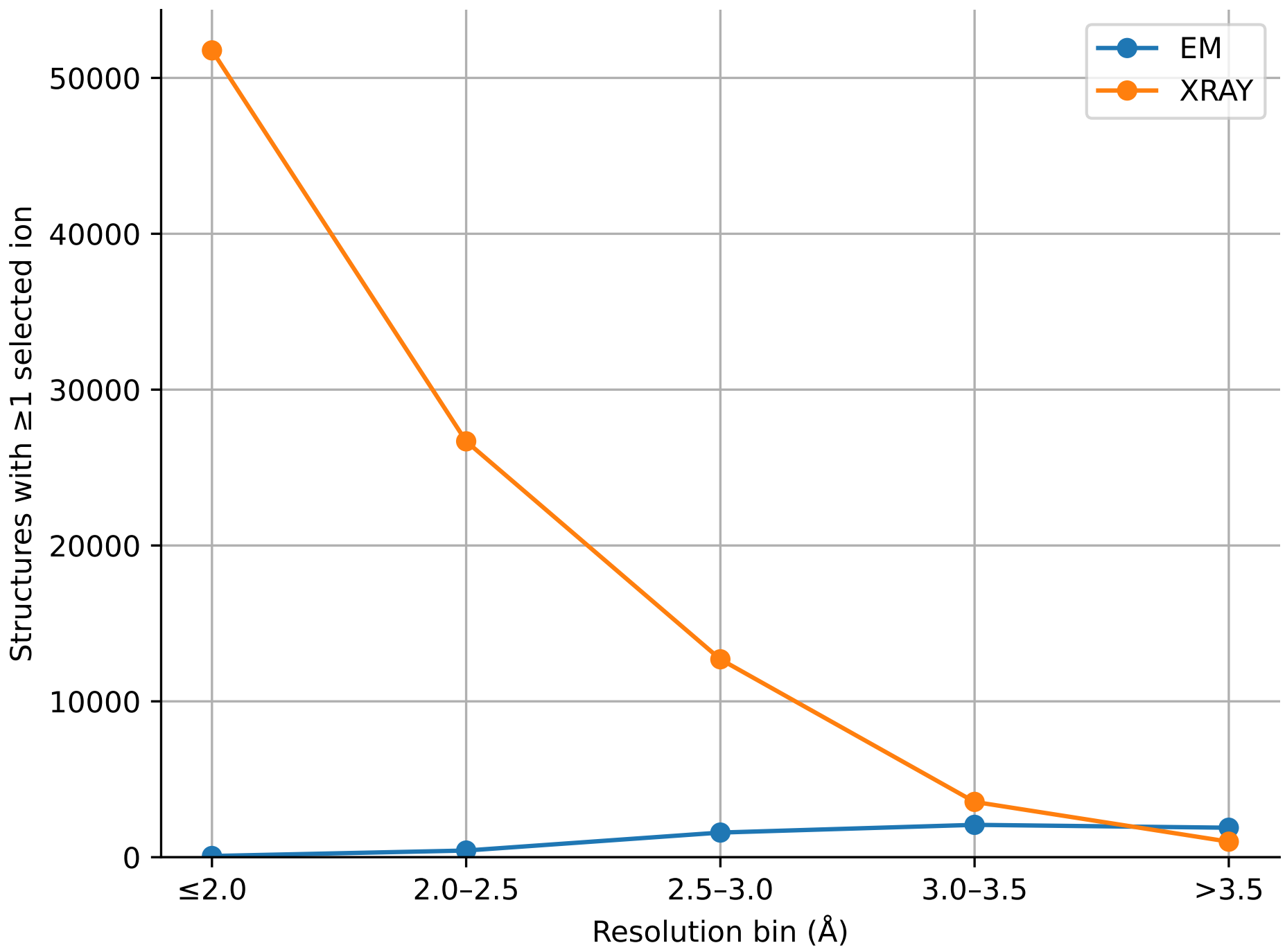

Supplementary Figure 11: **Number of PDB structures with at least one selected ion as a function of resolution and experimental method.** For all PDB entries deposited before 1 January 2025 that are available in legacy PDB format, we counted X-ray (orange) and cryo-EM (blue) structures that contain at least one of the ion types used in this study (see Supplementary Table 10). Resolution bins are ≤ 2.0, 2.0–2.5, 2.5–3.0, 3.0–3.5 and > 3.5 Å.

We also compared the local coordination geometry of Mg^{2+} and Zn^{2+} binding sites in X-ray and cryo-EM structures (Supplementary Figure 12). We focused on Mg^{2+} and Zn^{2+} because they are the two most abundant metal ions in both the X-ray and cryo-EM subsets in our dataset and thus provide representative examples. For each metal site we collected all neighboring heavy atoms (O, N, S) from protein residues, ligands and water molecules within 3 Å of the ion, and computed (i) the coordination number, and (ii) the distance between the ion and the centroid of its coordinating atoms ("off-centering"). In total we identified 40,046 Mg^{2+} and 41,593 Zn^{2+} sites in X-ray structures and 4,376 Mg^{2+} and 1,925 Zn^{2+} sites in cryo-EM structures. For Mg^{2+}, the mean coordination numbers were 4.08 for X-ray and 3.63 for cryo-EM, with similar off-centering (median 0.76 Å for X-ray and 0.73 Å for cryo-EM). Zn^{2+} sites showed more pronounced differences: the mean coordination number decreased from 4.93 (median 5 ligands within 3 Å) in X-ray structures to 3.58 (median 4 ligands) in cryo-EM structures, and the median off-centering increased from 0.65 Å in X-ray to 1.14 Å in cryo-EM. Overall, high-resolution cryo-EM structures can provide metal sites with broadly similar local geometry to crystallographic structures, but metal coordination in deposited cryo-EM models is on average less complete and somewhat more distorted.

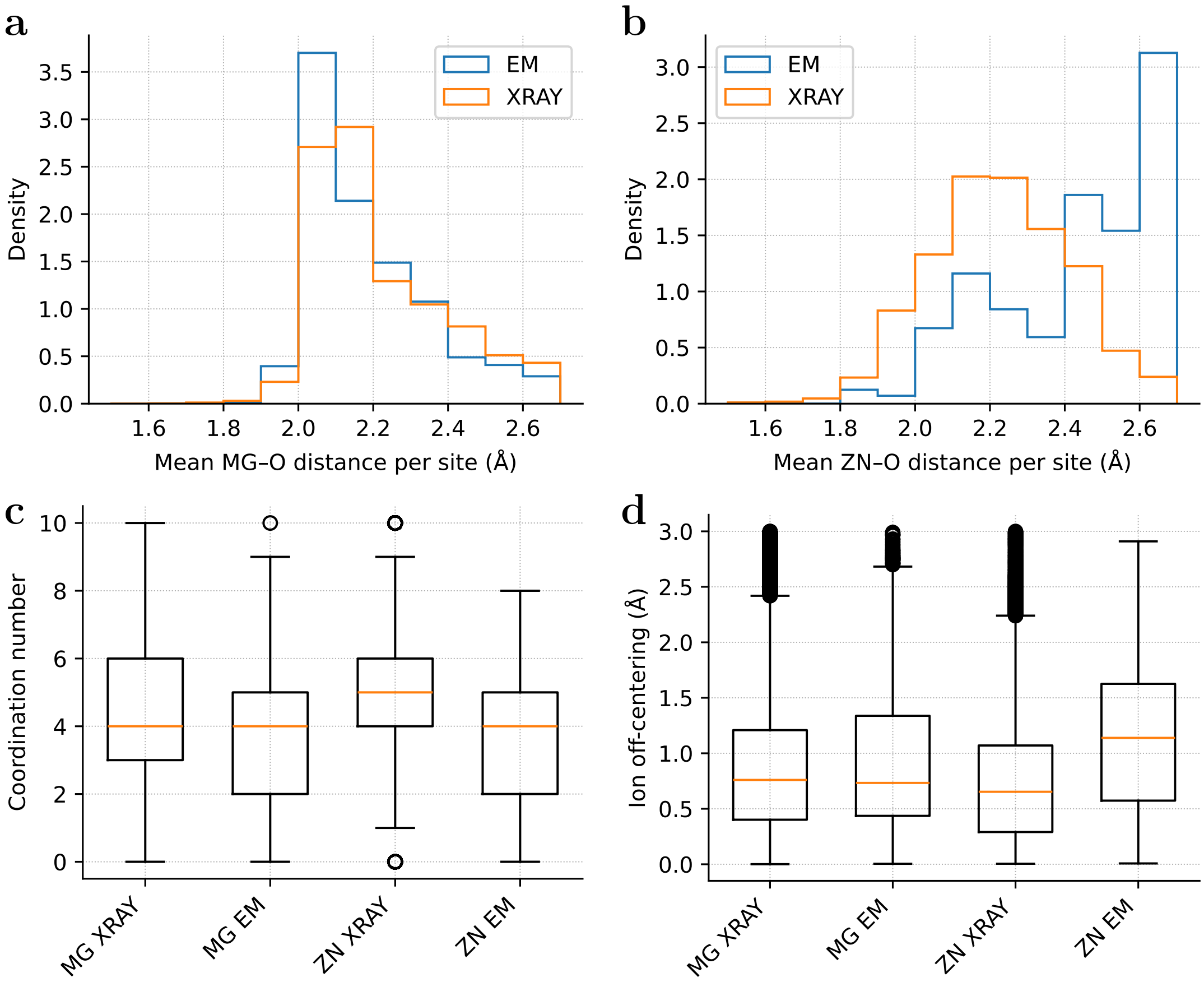

Supplementary Figure 12: **Comparison of local geometry of Mg^{2+} and Zn^{2+} binding sites in X-ray and cryo-EM structures.** For all PDB entries deposited before 1 January 2025 classified as X-ray or cryo-EM and containing Mg^{2+} or Zn^{2+}, we collected all neighboring heavy atoms (O, N, S) from protein residues, ligands and water molecules within 3 Å of the metal ion. Sample sizes are: n = 40,046 Mg^{2+} sites in X-ray structures, n = 4,376 Mg^{2+} sites in cryo-EM structures, n = 41,593 Zn^{2+} sites in X-ray structures, and n = 1,925 Zn^{2+} sites in cryo-EM structures. **a,b** Histograms of the mean metal–O distance per site for Mg^{2+} and Zn^{2+}, respectively, comparing X-ray (orange) and cryo-EM (blue) structures. **c** Coordination number distributions (number of O/N/S neighbors within 3 Å) for Mg^{2+} and Zn^{2+} in X-ray and cryo-EM structures. **d** Distributions of ion off-centering, defined as the distance between the ion and the centroid of its coordinating atoms. Mg^{2+} sites show similar mean distances and only slightly lower coordination numbers in cryo-EM compared to X-ray, whereas Zn^{2+} sites in cryo-EM tend to have fewer coordinating atoms and noticeably larger off-centering than in X-ray structures. Boxplots show the median and interquartile range, with whiskers extending to 1.5 times the interquartile range.

Supplementary Table 14: Number of total structures/chains/ligands in each split.

Split	Structures	Chains	Ligands
Train	2537	4388	21141
Fold 0	1694	3073	11210
Fold 1	2020	4410	9230
Fold 2	1773	4159	8536
Fold 3	1352	3211	9510
Fold 4	1588	3234	8789
Test	1770	3495	6442

Supplementary Table 15: Number of ions of specific ion type in each split.

	Class	Train	Fold 1	Fold 2	Fold 3	Fold 4	Fold 5	Test
0	-3	661	585	585	546	584	577	396
1	-2	5347	2434	1773	1831	1767	1736	1622
2	-1	6937	2601	2560	1815	2295	2019	1305
3	+1	2916	1788	1027	1162	1571	1317	823
4	+2	4905	3451	2907	2945	3143	2970	2170
5	+3	255	351	375	237	150	170	126
6	CA	2146	616	613	615	616	634	583
7	CL	3478	1164	1742	1169	1451	1185	737
8	CO	133	212	162	163	229	164	174
9	CU	44	236	184	182	421	190	23
10	FE	161	149	264	146	146	156	86
11	FE2	114	77	77	114	74	75	43
12	K	91	210	209	210	210	211	119
13	MG	326	597	608	779	604	607	388
14	MN	346	236	343	237	251	234	160
15	NA	2475	757	752	773	773	767	614
16	NI	158	157	154	154	183	233	115
17	PO4	661	578	578	546	575	577	390
18	SO4	5255	2015	1730	1732	1730	1729	1576
19	ZN	1308	656	656	658	657	658	559

Supplementary Table 16: 3D CNN model architecture used in the BiteNet_I method. Conv3D layer is the regular 3D convolutional layer and Conv3Dpool is the 3D convolutional layer with stride equal to 2. All layers except the last one are followed by the batch normalization layer and rectifier linear unit (ReLU) activation function. We apply a sigmoid activation function to the outputs of the last layer to restrict confidence scores and coordinates within cell to lay between 0 and 1. N_classes is the number of the ion classes, which is equal to 21 for the BiteNet_I model.

Index	Input shape	Output shape	Layer	Kernel	Stride	Filters
1	$64 \times 64 \times 64 \times 11$	$64 \times 64 \times 64 \times 64$	Conv3D	3	1	64
2	$64 \times 64 \times 64 \times 64$	$32 \times 32 \times 32 \times 64$	Conv3Dpool	3	2	64
3	$32 \times 32 \times 32 \times 64$	$32 \times 32 \times 32 \times 128$	Conv3D	3	1	128
4	$32 \times 32 \times 32 \times 128$	$32 \times 32 \times 32 \times 128$	Conv3D	3	1	128
5	$32 \times 32 \times 32 \times 128$	$16 \times 16 \times 16 \times 128$	Conv3Dpool	3	2	128
6	$16 \times 16 \times 16 \times 128$	$16 \times 16 \times 16 \times 256$	Conv3D	3	1	256
7	$16 \times 16 \times 16 \times 256$	$16 \times 16 \times 16 \times 512$	Conv3D	3	1	512
8	$16 \times 16 \times 16 \times 512$	$16 \times 16 \times 16 \times (N_\text{classes} \cdot 4)$	Conv3D	1	1	$N_\text{classes} \cdot 4$

Supplementary Table 17: List of ion classes predicted by the BiteNet_I model and PDB residue names of ions included into these classes.

Index	Class	Residue names
1	-3	PO3, PO4, VO4
2	-2	BF2, CO3, FPO, MOO, PER, PI, SE4, SO3, SO4, WO4
3	-1	2FK, 2HP, ACT, ALF, AZI, BCT, BEF, BO4, BR, BSY, CAC, CL, CYN, F, I3M, IOD, LCO, LCP, OH, SCN, SEK, VN3
4	+1	3P8, AG, AU, CS, CU1, EMC, K, LI, MAC, MMC, NA, ND4, NH4, NO2, NO3, PBM, RB, TL, TMA
5	+2	BA, CA, CD, CE, CF, CO, CU, EU, FE2, HG, IUM, MG, MN, MH2, NI, PB, PD, PT, PTN, SR, Y1, YB2, ZN
6	+3	3CO, 3NI, AL, AM, AU3, BS3, CON, CR, DY, ER3, EU3, FE, GA, GD3, IN, IR3, LA, LU, MN3, OS, PR, RH3, RU, SB, SM, TB, V, YB, YT3, ZCM
7	CA	CA
8	CL	CL
9	CO	CO
10	CU	CU
11	FE	FE
12	FE2	FE2
13	K	K
14	MG	MG
15	MN	MN
16	NA	NA
17	NI	NI
18	PO4	PO4
19	SO4	SO4
20	ZN	ZN

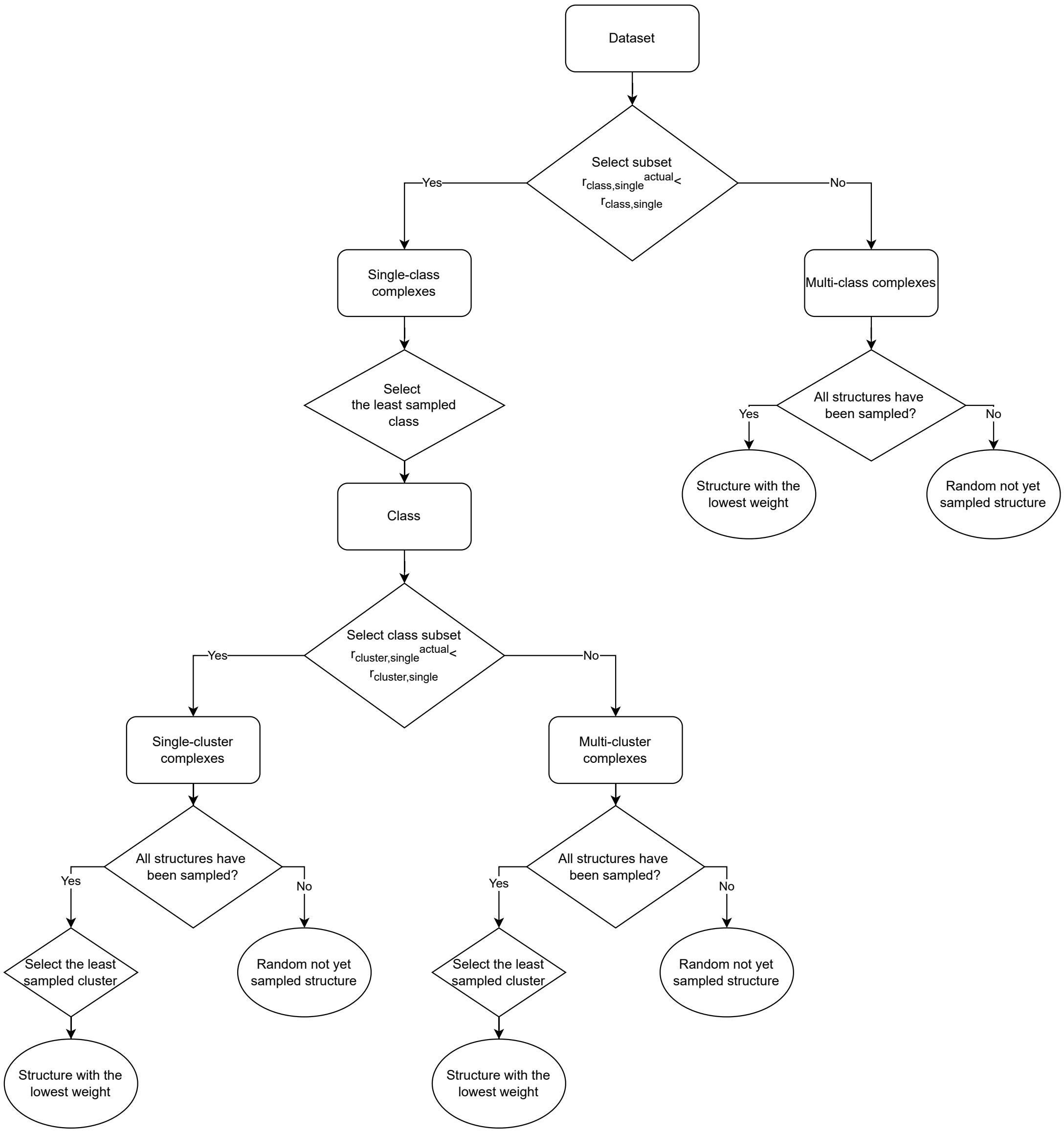

Supplementary Figure 13: **Schematic representation of workflow of complex sampling during training.** At each sampling step the algorithm first decides whether to draw a complex from the subset containing ions of a single class or from the subset containing multiple ion classes, so as to maintain their original proportions in the training set. For the single-class subset, the least-sampled ion class is selected, and its complexes are further split into those belonging to a single binding-site cluster ("single-cluster complexes") and those containing ions from multiple clusters ("multi-cluster complexes"); sampling alternates between these subsets to preserve their relative frequencies. Within the final subset, structures that have not yet been seen are drawn uniformly at random; once every structure has been sampled at least once, the algorithm repeatedly selects the least-sampled binding-site cluster and, within it, the complex with the lowest sampling weight (number of previous draws multiplied by the number of ions in the complex).

Supplementary Table 18: Parameters for functions converting the center-based predictions' scores into the scores of amino acid residues, described in Section Metrics in the main text.
Functions:
'constant': $f(d, r_{\text{norm}}) = 1$;
'linear': $f(d, r_{\text{norm}}) = \max(0, 1 - \frac{d}{r_{norm}})$;
'gaussian': $f(d, r_{\text{norm}}) = \exp(-\frac{d^2}{2r_{norm}^2})$;
'exponent': $f(d, r_{\text{norm}}) = \exp(-\frac{d}{r_{norm}})$;
'inverse': $f(d, r_{\text{norm}}) = \min(1, \frac{r_{norm}}{2d})$.

Train set	Ion class	Distance threshold	Function	r_{norm}	Score threshold
MIonSite train	ZN	5.0	linear	9.0	0.53
	CA	5.0	gaussian	4.0	0.50
	MG	4.0	linear	7.0	0.38
	MN	5.0	inverse	6.0	0.68
	FE	4.0	inverse	7.0	0.81
	CU	5.0	inverse	8.0	0.80
	FE2	4.0	gaussian	8.0	0.82
	CO	4.0	exponent	8.0	0.53
	NA	5.0	inverse	7.0	0.70
	K	5.0	inverse	9.0	0.86
	CD	5.0	linear	7.0	0.37
	NI	4.0	gaussian	10.0	0.88
IonCom Fold 1	CA	5.0	inverse	7.0	0.84
	CO3	5.0	inverse	8.0	0.78
	CU	4.0	linear	7.0	0.46
	FE	5.0	gaussian	7.0	0.79
	FE2	4.0	inverse	7.0	0.87
	K	5.0	inverse	9.0	0.87
	MG	3.0	gaussian	7.0	0.57
	MN	5.0	gaussian	4.0	0.56
	NA	5.0	linear	6.0	0.26
	NO2	5.0	linear	6.0	0.26
	PO4	5.0	inverse	9.0	0.65
	SO4	5.0	inverse	8.0	0.54
	ZN	4.0	gaussian	6.0	0.80
IonCom Fold 2	CA	5.0	gaussian	5.0	0.71
	CO3	5.0	inverse	8.0	0.64
	CU	4.0	inverse	5.0	0.67
	FE	5.0	inverse	7.0	0.78
	FE2	4.0	constant	4.0	0.84
	K	5.0	inverse	8.0	0.71
	MG	4.0	gaussian	9.0	0.53
	MN	4.0	inverse	6.0	0.67
	NA	5.0	linear	7.0	0.33
	NO2	5.0	inverse	8.0	0.84
	PO4	5.0	constant	5.0	0.64
	SO4	5.0	linear	7.0	0.25
	ZN	4.0	exponent	10.0	0.68
IonCom Fold 3	CA	4.0	gaussian	5.0	0.69
	CO3	5.0	inverse	8.0	0.62
	CU	4.0	inverse	6.0	0.75
	FE	5.0	inverse	7.0	0.76
	FE2	5.0	inverse	7.0	0.85
	K	5.0	constant	5.0	0.47
	MG	4.0	inverse	5.0	0.56
	MN	4.0	exponent	7.0	0.52

	NA	5.0	linear	7.0	0.29
	NO2	5.0	inverse	8.0	0.88
	PO4	5.0	gaussian	10.0	0.55
	SO4	5.0	exponent	7.0	0.29
	ZN	4.0	linear	10.0	0.63
IonCom Fold 4	CA	5.0	exponent	6.0	0.49
	CO3	5.0	inverse	8.0	0.71
	CU	4.0	linear	9.0	0.58
	FE	5.0	inverse	7.0	0.82
	FE2	4.0	linear	10.0	0.60
	K	5.0	constant	5.0	0.89
	MG	5.0	inverse	6.0	0.66
	MN	5.0	linear	9.0	0.50
	NA	4.0	constant	4.0	0.56
	NO2	5.0	linear	9.0	0.51
	PO4	5.0	inverse	8.0	0.55
	SO4	5.0	linear	8.0	0.28
	ZN	4.0	linear	9.0	0.62
IonCom Fold 5	CA	4.0	inverse	6.0	0.73
	CO3	5.0	constant	5.0	0.52
	CU	4.0	inverse	6.0	0.75
	FE	5.0	gaussian	5.0	0.64
	FE2	4.0	gaussian	9.0	0.84
	K	5.0	inverse	8.0	0.78
	MG	4.0	inverse	6.0	0.59
	MN	5.0	linear	9.0	0.52
	NA	5.0	linear	7.0	0.31
	NO2	5.0	inverse	7.0	0.76
	PO4	5.0	exponent	9.0	0.37
	SO4	5.0	gaussian	4.0	0.28
	ZN	4.0	inverse	6.0	0.79
BiteNet Train	-3	5.0	inverse	8.0	0.77
	-2	5.0	inverse	8.0	0.72
	-1	5.0	inverse	7.0	0.78
	+1	5.0	inverse	8.0	0.83
	+2	5.0	gaussian	8.0	0.85
	+3	6.0	inverse	6.0	0.77
	CA	5.0	inverse	7.0	0.86
	CL	4.0	inverse	7.0	0.78
	CO	5.0	inverse	7.0	0.89
	CU	5.0	gaussian	7.0	0.83
	FE	6.0	inverse	7.0	0.91
	FE2	5.0	gaussian	9.0	0.90
	K	5.0	inverse	9.0	0.87
	MG	5.0	inverse	6.0	0.79
	MN	5.0	gaussian	5.0	0.73
	NA	5.0	inverse	8.0	0.86
	NI	4.0	gaussian	10.0	0.92
	PO4	5.0	inverse	8.0	0.77
	SO4	5.0	inverse	8.0	0.75
	ZN	5.0	inverse	6.0	0.74

References

[1] Liang Qiao and Dongqing Xie. Mionsite: Ligand-specific prediction of metal ion-binding sites via enhanced adaboost algorithm with protein sequence information. *Analytical biochemistry*, 566:75–88, 2019. doi: 10.1016/j.ab.2018.11.009.

[2] Aditi Shenoy, Yogesh Kalakoti, Durai Sundar, and Arne Elofsson. M-Ionic: Prediction of metal-ion-binding sites from sequence using residue embeddings. *Bioinformatics*, 40(1):btad782, January 2024. ISSN 1367-4811. doi: 10.1093/bioinformatics/btad782.

[3] Andrea Passerini, Marco Lippi, and Paolo Frasconi. Metaldetector v2. 0: predicting the geometry of metal binding sites from protein sequence. *Nucleic acids research*, 39(suppl_2):W288–W292, 2011. doi: 10.1093/nar/gkr365.

[4] Jianyi Yang, Ambrish Roy, and Yang Zhang. Protein–ligand binding site recognition using complementary binding-specific substructure comparison and sequence profile alignment. *Bioinformatics*, 29(20):2588–2595, 2013. doi: 10.1093/bioinformatics/btt447.

[5] Dong-Jun Yu, Jun Hu, Jing Yang, Hong-Bin Shen, Jinhui Tang, and Jing-Yu Yang. Designing template-free predictor for targeting protein-ligand binding sites with classifier ensemble and spatial clustering. *IEEE/ACM transactions on computational biology and bioinformatics*, 10(4):994–1008, 2013. doi: 10.1109/TCBB.2013.104.

[6] Xiuzhen Hu, Qiwen Dong, Jianyi Yang, and Yang Zhang. Recognizing metal and acid radical ion-binding sites by integrating ab initio modeling with template-based transferals. *Bioinformatics*, 32 (21):3260–3269, 2016. doi: 10.1093/bioinformatics/btw396.

[7] Yu-Feng Lin, Chih-Wen Cheng, Chung-Shiuan Shih, Jenn-Kang Hwang, Chin-Sheng Yu, and Chih-Hao Lu. Mib: metal ion-binding site prediction and docking server. *Journal of chemical information and modeling*, 56(12):2287–2291, 2016. doi: 10.1021/acs.jcim.6b00407.

[8] Qianmu Yuan, Sheng Chen, Yu Wang, Huiying Zhao, and Yuedong Yang. Alignment-free metal ion-binding site prediction from protein sequence through pretrained language model and multi-task learning. *Briefings in Bioinformatics*, 23(6):bbac444, November 2022. ISSN 1477-4054. doi: 10.1093/bib/bbac444.

[9] Vinícius A Paiva, Murillo V Mendonça, Sabrina A Silveira, David B Ascher, Douglas E V Pires, and Sandro C Izidoro. GASS-Metal: Identifying metal-binding sites on protein structures using genetic algorithms. *Briefings in Bioinformatics*, 23(5):bbac178, September 2022. ISSN 1477-4054. doi: 10.1093/bib/bbac178.

[10] Simon L. Dürr, Andrea Levy, and Ursula Rothlisberger. Metal3D: A general deep learning framework for accurate metal ion location prediction in proteins. *Nature Communications*, 14(1):2713, May 2023. ISSN 2041-1723. doi: 10.1038/s41467-023-37870-6.

[11] Simon L. Dürr and Ursula Rothlisberger. AllMetal3D: Joint prediction of localization, identity and coordination geometry of common metal ions in proteins, February 2025.

[12] Hamza Gamouh, Marian Novotný, and David Hoksza. Hybrid protein–ligand binding residue prediction with protein language models: Does the structure matter? *Bioinformatics*, 41(8):btaf431, August 2025. ISSN 1367-4811. doi: 10.1093/bioinformatics/btaf431.

[13] Josh Abramson, Jonas Adler, Jack Dunger, Richard Evans, Tim Green, Alexander Pritzel, Olaf Ronneberger, Lindsay Willmore, Andrew J. Ballard, Joshua Bambrick, Sebastian W. Bodenstein, David A. Evans, Chia-Chun Hung, Michael O'Neill, David Reiman, Kathryn Tunyasuvunakool, Zachary Wu, Akvilė Žemgulytė, Eirini Arvaniti, Charles Beattie, Ottavia Bertolli, Alex Bridgland, Alexey Cherepanov, Miles Congreve, Alexander I. Cowen-Rivers, Andrew Cowie, Michael Figurnov, Fabian B. Fuchs, Hannah Gladman, Rishub Jain, Yousuf A. Khan, Caroline M. R. Low, Kuba Perlin, Anna Potapenko, Pascal Savy, Sukhdeep Singh, Adrian Stecula, Ashok Thillaisundaram, Catherine Tong, Sergei Yakneen, Ellen D. Zhong, Michal Zielinski, Augustin Žídek, Victor Bapst, Pushmeet Kohli, Max Jaderberg, Demis Hassabis, and John M. Jumper. Accurate structure prediction of biomolecular interactions with AlphaFold 3. *Nature*, 630(8016):493–500, June 2024. ISSN 1476-4687. doi: 10.1038/s41586-024-07487-w.

[14] John Jumper, Richard Evans, Alexander Pritzel, Tim Green, Michael Figurnov, Olaf Ronneberger, Kathryn Tunyasuvunakool, Russ Bates, Augustin Žídek, Anna Potapenko, et al. Highly accurate protein structure prediction with alphafold. *Nature*, 596(7873):583–589, 2021. doi: 10.1038/s41586-021-03819-2.
