# Supplementary material for: Multivalent ion binding site identification with structure-based deep learning
Source: Commun Biol. 2026 Jul 27;9:1039. doi: 10.1038/s42003-026-10659-1 (PMC13434133; doi:10.1038/s42003-026-10659-1)
Supplement: Supplementary file 3 [file 42003_2026_10659_MOESM3_ESM.pdf]

Description of Additional Supplementary Files

File name: Supplementary Data 1

Description: The source data underlying the quantitative graphs in the main figures, including weighted average precision scores in Figure 1b, IonCom benchmark MCC values in Fig. 2, resolution-dependent AP scores in Figue 4a, GPCR sodium-pocket prediction scores in Figure 4b, resolution-dependent AP scores for each ion class in Supplementary Figure 7.
